# Tinosporae Radix attenuates acute pharyngitis by regulating glycerophospholipid metabolism and inflammatory responses through PI3K-Akt signaling pathway

**DOI:** 10.3389/fphar.2024.1491321

**Published:** 2024-11-06

**Authors:** Lijie Lu, Chengfeng Huang, Yongfeng Zhou, Huajuan Jiang, Cuiping Chen, Jinyu Du, Tao Zhou, Feiyan Wen, Jin Pei, Qinghua Wu

**Affiliations:** ^1^ State Key Laboratory of Southwestern Chinese Medicine Resources, Chengdu University of Traditional Chinese Medicine, Chengdu, China; ^2^ College of Pharmacy, Chengdu University of Traditional Chinese Medicine, Chengdu, China; ^3^ Department of Pharmacy, The First Affiliated Hospital of Anhui University of Chinese Medicine, Hefei, China

**Keywords:** Tinosporae Radix, acute pharyngitis, PI3K-Akt signaling pathway, glycerophospholipid metabolism, inflammatory responses

## Abstract

**Introduction:**

With the onset of the COVID-19 pandemic, the incidence and prevalence of acute pharyngitis (AP) have increased significantly. Tinosporae Radix (TR) is a vital medication utilized in the treatment of pharyngeal and laryngeal ailments, especially AP. The study endeavors to explore unclear molecular mechanisms of TR in addressing AP.

**Methods:**

Network pharmacology and metabolomics analyses of effect of TR on AP were conducted, and apossible pathway was validated both *in vivo* using the acute pharyngitis rat model and *in vitro* using the LPS-induced RAW264.7 cells model, through techniques such as histopathological examinations, immunohistochemical technology, ELISA, RT-qPCR, and Western blotting to systematically explore the possible mechanisms underlying the inhibition of AP by TR.

**Results and discussion:**

Network pharmacology analysis identified several key targets, including PIK3CA, IL6, AKT1, TNF, and PTGS2, alongside pivotal signaling pathways such as IL-17, TNF, Hepatitis B, nuclear factor kappa B (NF-κB), Influenza A, and the PI3K-Akt pathway. Most of them are closely associated with inflammation. Then, wide-target metabolomics analysis showed that TR downregulated substances within the glycerophospholipid metabolic pathway, and modulated the PI3K-Akt pathway. The integrated findings from network pharmacology and metabolomics underscored the pivotal role of the PI3K-Akt signaling pathway and the attenuation of inflammatory responses. Finally, *in vitro* and *in vivo* experiments have shown that TR can inhibit inflammatory factors such as IL-6, TNF - α, and COX-2, downregulate targets such as PI3K and AKT on the PI3K-Akt signaling pathway, and thereby alleviate the inflammatory response of AP. Our study demonstrated that TR exerts an anti-AP effect through suppression of release of inflammatory factors and modulation of glycerophospholipid metabolism via suppressing the PI3K-Akt signaling pathway.

## Graphical Abstract

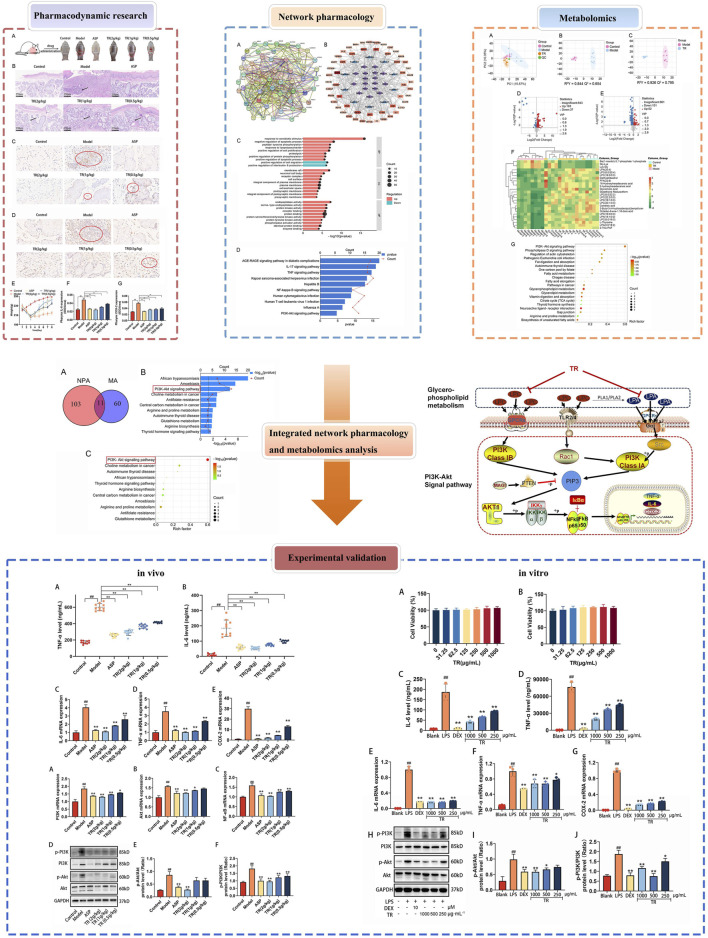

## Highlights

• Integrated network pharmacology, metabolomics, and experimental validation have identified the pivotal pathway through which TR operates in the treatment of acute pharyngitis: the PI3K-Akt signaling pathway.
• By inhibiting the PI3K-Akt pathway, Tinosporae Radix suppresses the glycerophospholipid metabolism, thereby counteracting acute pharyngitis.
• Tinosporae Radix mitigates the inflammatory response associated with acute pharyngitis by inhibiting the release of inflammatory factors, such as IL-6 and TNF-α, as well as their mRNA expression, through modulation of the PI3K-Akt pathway.

## 1 Introduction

Acute pharyngitis (AP) represents a prevalent clinical condition within the realm of epidemiology, imposing significant medical and societal burdens (Krüger et al., 2021; Nishikimi et al., 2022). Its incidence has notably surged amidst the backdrop of the COVID-19 pandemic (Weiser et al., 2018; Davis et al., 2023). Acute pharyngitis manifests as a sudden inflammation of the pharyngeal mucosa and submucosal tissues, with its etiology encompassing both infectious and non-infectious factors. Infectious culprits include viruses, bacteria, *mycoplasma*, and *chlamydia* infections, while non-infectious triggers comprise substances like dust, smoke, irritant gases, and mechanical irritation (Bisno, 2001; Vincent et al., 2004). Clinical manifestations commonly include dryness, itching, and burning sensations in the pharynx, along with dry cough, a sensation of a foreign body in the throat, pharyngeal pain, and hoarseness. Acute pharyngitis exerts profound impacts on human health, daily functioning, and occupational productivity, characterized by its recalcitrance, seasonal variability, recurrent nature, and diverse causative agents. In addressing acute pharyngitis, the Infectious Diseases Society of America recommends the utilization of aspirin, nonsteroidal anti-inflammatory agents (NSAIAs), glucocorticoids, or penicillin (Krüger et al., 2021). However, reliance solely on Western medicine for pharyngitis treatment can pose challenges. These may include a limited treatment spectrum, susceptibility to recurrence, poor tolerance, adverse toxic effects, and the potential for drug resistance (Pellegrino et al., 2023). Such issues can significantly impact patient adherence and the overall effectiveness of pharmacological interventions. Hence, there is an urgent need to discover and develop safe and efficacious drugs or strategies for treating acute pharyngitis, while concurrently elucidating the underlying molecular mechanisms involved in its prevention.

Chinese herbs represent a promising avenue for tackling a multitude of ailments, owing to their cost-effectiveness, wide accessibility, and relatively lower incidence of adverse effects (Lu et al., 2022). This has prompted extensive exploration into alternative therapeutic modalities leveraging Chinese herbs for various conditions. Notably, there has been considerable interest in investigating the potential of Chinese herbs for managing acute pharyngitis (Zhou et al., 2018; Miao et al., 2019; Xu et al., 2020). Tinosporae Radix (TR), known as “Jin Guo Lan” (金果榄) in Chinese, is derived from the dried root of Tinospora sagittata (Oliv.) Gagnep. Species within the genus Tinospora have traditionally served as therapeutic remedies. In India, T. cordifolia, known for its immune-regulatory properties (Wajpeyi and Ashish, 2023), is documented for treating gastrointestinal diseases (Arunachalam et al., 2021) and metabolic disorders, including diabetes and kidney issues, which are currently receiving increased research attention (Patial et al., 2021). Additionally, it is prescribed for intermittent fevers, infectious conditions, urinary disorders, skin diseases, and eye disorders (Panchabhai et al., 2008; Chi et al., 2016). Chinese Tibetan medicine, Dai Medicine and Thailand’s Lanna Medicine document that T. sinensis can be clinically used to treat muscle stiffness, arthritis, palpitations, high fever, and diabetes, and to improve physical frailty (Chi et al., 2016; Haque et al., 2017). The “Bencao Gangmu Shiyi” emphasizes the exceptional efficacy of T. sagittata (TR) in treating all diseases of the pharynx and throat (Zhao, 1765). It is frequently employed to alleviate inflammation and stomach pain among various ethnic minorities including the Miao, Zhuang, and Yao, etc (Haque et al., 2017). In addition, TR is commonly used in Dai medicine to treat rheumatism, Mulao nationality use its stem to heal trauma and rheumatism, and Wa people use its leaves to relieve redness in the eyes (Nicpbp N I F T C O P a B P, 1984). Apart from traditional medicine, contemporary pharmacological research and clinical studies also highlight the notable therapeutic effects of TR on the pharynx. Traditional Chinese medicine formulations featuring TR as the primary medication, such as Jinguolan decoction and Dikudan capsules, exhibit noticeable inhibitory effects on various inflammation models (Wang et al., 2009). Furthermore, they demonstrate significant efficacy in addressing acute and chronic pharyngitis as well as acute tonsillitis (Zhang et al., 2008; Xu et al., 2020). Studies indicate that TR’s anti-inflammatory properties stem from its alkaloids (palmatine and columbabine,etc), nitrogen-containing compounds, and diterpenoids (columbin) (Huang et al., 2012b). The mechanism involves inhibiting the production of NO (Huang et al., 2012a; Zhang et al., 2016) or the expression of iNOS, COX-2, and NF-κB (Ibrahim et al., 2012). For instance, palmatine and columbabine hinder NF-κB activation in RAW264.7 macrophages stimulated by TNF-α (Liu et al., 2010). Columbin, on the other hand, suppresses COX-2 activation by binding to active sites Tyr385 and Arg120 on the COX-2 enzyme (Ibrahim et al., 2012). Until now, the anti-inflammatory mechanism of TR has been extensively studied, while its anti-AP action mechanism remains largely unexplored.

In the current landscape, deciphering the pharmacological mechanisms of Traditional Chinese Medicine (TCM) remains a formidable challenge. However, with the advent of the big data era and artificial intelligence, the burgeoning field of information science offers unprecedented opportunities to transcend the constraints of conventional medical research methods (Zhang et al., 2023b). Systems pharmacology, which encompasses network pharmacology, molecular biology technologies, metabolomics, and transcriptomics, represents a potent interdisciplinary approach that integrates experimental assays with computational analyses (Mehta et al., 2022). Indeed, network pharmacology has emerged as a cornerstone in the study of TCM, facilitating the elucidation of molecular mechanisms governing their efficacy (Zhao et al., 2023). Grounded in systems biology principles, network pharmacology provides a robust methodology for assessing TCM’s pharmacological effects at the molecular level. It enables the comprehensive exploration of critical multi-channel regulation within signaling pathways and facilitates the identification of therapeutic modulation within metabolic networks (Jiao et al., 2022; Nogales et al., 2022; Zhang et al., 2024). Metabolomics, on the other hand, is commonly utilized to gain a comprehensive understanding of the alterations in endogenous metabolites within complex biological systems (Marchev et al., 2021). Its profiling endeavors to pinpoint potential biomarkers that shed light on the pathophysiology of human diseases (Johnson et al., 2016). Given Traditional Chinese Medicine’s distinctive attributes of multi-component and multi-target properties, metabolomics emerges as an attractive tool poised to play a pivotal role in unraveling the potential pharmacological mechanisms of TCM (Cuperlovic-Culf and Culf, 2016; Tian et al., 2022). In the present era, there is an urgent need to unravel the pharmacological mechanisms underlying the efficacy of Tinosporae Radix in treating acute pharyngitis through the integration of a comprehensive model encompassing network pharmacology, metabolomics, and biological methods.

In this study, we employed a combination of network pharmacology technology and metabolomics to delve into the anti-acute pharyngitis mechanism of Tinosporae Radix (Yu et al., 2024). Subsequently, through both *in vitro* and *in vivo* experiments, we validated this mechanism at the molecular level. Our findings highlight the robust anti-AP effects of TR in a 15% ammonia-induced rat model (Zhou et al., 2020), including the alleviation of pharyngitis symptoms and the suppression of inflammatory responses. Significantly, we elucidated that these therapeutic effects may be attributed to the inhibition of the PI3K-Akt signaling pathway and the regulation of glycerophospholipid metabolism (Xiao et al., 2022). Notably, this study represents the first comprehensive investigation and validation of TR using both *in vitro* and *in vivo* models, along with various omics and biological methods. Our findings lay a solid foundation for further exploration into the anti-AP mechanism of TR.

## 2 Materials and methods

### 2.1 Materials

Tinosporae Radix (TR) were gathered in October 2021 from Yunnan Province, Wenshan, China, and meticulously identified by Professor Jin Pei from the School of Pharmacy, Chengdu University of Traditional Chinese Medicine, Chengdu, China. The certificate specimens (CDUTCM-202110170001) have been meticulously preserved in the National Germplasm Resource Bank of Chinese Medicine at Chengdu University of Traditional Chinese Medicine.

Lipopolysaccharides (LPS) was procured from Sigma (St. Louis, MO, United States). ELISA Kits for rat IL-6 (Cat No. H007-1-2) and TNF-α (Cat No. H052-1-2) were sourced from Nanjing Jiancheng Bioengineering Institute (Nanjing, China), while ELISA Kits for mouse IL-6 (Cat No. EMC004QT.96) and TNF-α (Cat No. EMC102aQT.96) were obtained from Neobioscience (Shenzhen, China). Antibodies targeting GAPDH, PI3K/p-PI3K, Akt/p-Akt, and goat anti-rabbit IgG-HRP were procured from Affinity Biosciences (Inc., Cincinnati, OH, United States). The RIPA lysis buffer and a Bradford protein assay kit were obtained from Biyuntian Biotechnology (Shanghai, China). The cell counting kit-8 (CCK-8) was provided by Dojindo, and PBS was purchased from Zhongshan Jinqiao Biotechnology (Co., Ltd., Beijing, China).

### 2.2 Network pharmacology study

In the preliminary research conducted by our team, we utilized the UPLC-Q-TOF-MS method to analyze the chemical composition of TR (Lu et al., 2023). Our analysis revealed that TR predominantly consists of 17 active compounds, with the ADME parameters of these compounds detailed in Supplementary Table S1. To identify the targets of these active constituents, we conducted searches on the Swiss TargetPrediction database (http://www.swisstargetprediction.ch/) and the Traditional Chinese Medicines for Systems Pharmacology Database and Analysis Platform (TCMSP, http://tcmspw.com/tcmsp.php). Additionally, we utilized platforms such as GeneCards (https://www.genecards.org/), OMIM (https://www.omim.org/), and DisGeNet (https://www.disgenet.org) to acquire information on relevant anti-AP proteins for candidate target screening, with “acute pharyngitis” as the keyword. Subsequently, we utilized the STRING database (https://string-db.org/) to analyze the protein-protein interactions (PPI) of the candidate targets. Interactions with reliability scores (score >0.4) were selected after eliminating duplicates. For further insight, we employed the DAVID Bioinformatics Database 6.8 (https://david.ncifcrf.gov/) to conduct Gene Ontology (GO) enrichment analysis and Kyoto Encyclopedia of Genes and Genomes (KEGG) pathway analysis based on the identified core targets. To visually represent the relationships among compounds, targets, and pathways, we constructed a compound–target–pathway network using Cytoscape 3.9.0.

### 2.3 Animals and drug administration

#### 2.3.1 Animals

Sixty 7-week-old male SD rats were supplied by Byrness Weil Biotech Ltd. (SCXK Hunan, 2019–0004) and housed in a standard air-conditioned environment (25°C ± 2°C, 40%–50% R.H.). All animal experiments and care procedures were conducted following the guidelines approved by the Institutional Animal and Use Committee of Chengdu University of Traditional Chinese Medicine [Approval number: SYXK (CHUAN) 2014-128].

#### 2.3.2 Preparation of TR extract

We prepared TR extract as follows: accurately weighing 80 g of TR powder that had passed through the third sieve, we added 640 mL of 70% methanol followed by 320 mL of the same solvent. After sonication for 30 min each, the mixture was centrifuged, and the filtrate was collected. The solvent was evaporated in a 95°C water bath. Subsequently, 400 mL of reverse osmosis water was added, followed by sonication to obtain a high-dose solution. Finally, medium and low-dose solutions were prepared by diluting the high-dose solution twice and four times, respectively.

#### 2.3.3 Drug administration

A total of 60 rats were randomly assigned to six groups: the control group, model group, aspirin (ASP) group (positive control), TR low-dose group, TR medium-dose group, and TR high-dose group. The acute pharyngitis model was induced by spraying 15% ammonia water on the pharynx of rats from day 1 to day 3, with 3 pumps sprayed each time, leading to hyperemia and swelling of the pharyngeal mucosa, resulting in inflammation. The control group was sprayed with an equal volume of saline. From day 4 to day 8, rats in the ASP group (positive control) received treatment with an aspirin solution orally, at a dosage of 200 mg/kg/day. The rats in the low-dose, medium-dose, and high-dose TR groups received TR orally, at doses of 0.5 g/kg/day, 1 g/kg/day, or 2 g/kg/day, respectively. In contrast, the rats in the control group and model group were administered with the same volume of 0.3% sodium carboxymethyl cellulose (CMC-Na) orally on a daily basis. It's worth noting that both ASP and TR were dissolved in 0.3% CMC-Na at appropriate concentrations. The dosage of the positive control drug, ASP, was set as per previous reports (Wang et al., 2024), while the doses of TR were determined based on the dosage regulations outlined in the Chinese Pharmacopoeia, and then adjusted according to body surface area conversion.

### 2.4 Pharmacodynamic evaluation of TR in AP rats

#### 2.4.1 Histopathological analysis

The pharyngeal tissue of rats was surgically excised and subsequently fixed in 4% paraformaldehyde for 24 h. After fixation, the tissue samples underwent dehydration, embedding in paraffin, and sectioning. Morphological analysis was conducted utilizing hematoxylin-eosin (H-E) staining, and images of the stained sections were acquired using an optical microscope.

#### 2.4.2 Immunohistochemical analysis

The pharyngeal tissues of rats were fixed, embedded, sectioned, deparaffinized, and hydrated, followed by incubation with anti-IL6 or anti-COX-2 antibodies. Finally, after treatment with secondary antibodies, the immunostaining was observed under a fluorescence microscope.

### 2.5 Metabonomic analysis

2 μL of processed serum samples from the Control, Model, and TR high-dose groups were injected for analysis using ultra-high-performance liquid chromatography tandem mass spectrometry (UPLC-ESI-MS/MS). The chromatographic analysis was conducted on the ExionLC AD UPLC system (Agilent Corporation, Santa Clara, CA, United States), and the serum samples were analyzed on a Waters ACQUITY UPLC HSS T3 C18 column (100 mm × 2.1 mm, 1.8 μm). The column temperature was maintained at 40°C, and the elution process was carried out at a flow rate of 0.4 mL·min^−1^. The mobile phase used consisted of two components: 0.1% formic acid (A) and acetonitrile containing 0.1% formic acid (B). For mass spectrometry analysis, a triple quadrupole-linear ion trap mass spectrometer (QTRAP) from AB Sciex Pte. Ltd. was utilized. The instrument was equipped with an electrospray ionization (ESI) source capable of operating in both positive and negative ionization modes. Specific parameters for the mass spectrometer were set as follows: drying gas temperature, 500°C; nebulizer gas pressure, 55 psi; ion spray voltage, 5,500 V (positive), −4,500 V (negative); ion source gas I, gas II, and curtain gas were set at 55, 60, and 25 psi, respectively; the collision gas was high. The data were collected based on the mass-to-charge ratio (m/z) of the parent ion. To ensure accurate mass determination and optimal instrument performance, instrument tuning and mass calibration were conducted using 10 and 100 μmol·L^−1^ polypropylene glycol solutions in QQQ and LIT modes, respectively. Metabolite identification was achieved by searching for the exact molecular masses of both parent and daughter ions against a custom-built targeted standard product database known as MWDB (Metware Database). This database integrates information from reputable sources such as the Human Metabolome Database (HMDB, http://www.hmdb.ca/) and METLIN (http://metlin.scripps.edu/). Principal component analysis (PCA) and orthogonal partial least squares discriminant analysis (OPLS-DA) were performed on the metabolites of all groups. The threshold for variable importance in projection (VIP) was set at VIP ≥1, and the statistical significance of metabolic changes between different groups was set at a *p*-value <0.05. Following identification, metabolites were annotated using the KEGG Compound database (http://www.kegg.jp/kegg/compound/), and subjected to metabolite set enrichment analysis.

### 2.6 Validation *in vivo*

#### 2.6.1 Determination of cytokines by ELISA

Serum concentrations of interleukin-6 (IL-6) and tumor necrosis factor-alpha (TNF-α) were quantified following the manufacturer’s instructions.

#### 2.6.2 Quantitative real-time polymerase chain reaction

Extract total RNA and reverse transcribe RNA from rat pharyngeal tissue according to the instructions of the kit. For quantitative polymerase chain reaction (qPCR), a reaction system was established, with GAPDH serving as the internal reference gene. The qPCR reaction conditions were as follows: initial denaturation at 95°C for 30 s, followed by denaturation at 95°C for 5 s, annealing at 62.9°C for 30 s. The reaction consisted of 40 cycles. The results were quantified using the 2^−ΔΔCt^ method to assess the expression levels of the target genes. The primers used for amplifying the target genes were sourced from Chengdu Tsingke Biotech Co., Ltd. in China (refer to Table 1).

**TABLE 1:** The sequences of primers.

Primer name	Primer sequence
GAPDH	F:5′-GACTCTACCCACGGCAAGTT-3′
	R:5′-GGTGATGGGTTTCCCGTTGA-3′
COX-2	F:5′-ATGACTTCCCTGGGTTTGGT-3′
	R:5′-GTCCCCCATTGTGGTATCTG-3′
IL-6	F:5′-CAGACCAGTATATACCACTTC-3′
	R:5′-ATATCCAGTTTGGAAGCATCC-3′
TNF-α	F:5′-GCCTCTTCTCATTCCTGCTT-3′
	R:5′-TGGGAACTTCTCATCCCTTTG-3′
PI3K	F:5′-CTCCGAGCACTTTGTACCCG-3′
	R:5′-CTGGGCCACTTCATCTCTGG-3′
Akt	F:5′-ATGGACTCAAACGGCAGGAG-3′
	R:5′-TCCTTGGCAACGATGACCTC-3′
NF-κB	F:5′-CAGTGTATCGGTGGTCAGTGT-3′
	R:5′-GGATGATGTTGGCAGCAATGG-3′

#### 2.6.3 Western blotting

After preparing denatured protein samples of rat pharyngeal tissue, we performed electrophoresis, membrane transfer, and blocking. The samples were then incubated with primary antibodies targeting phosphorylated AKT (p-AKT), total AKT, phosphorylated phosphatidylinositol 3-kinase (p-PI3K), total phosphatidylinositol 3-kinase (PI3K), and GAPDH. Finally, following incubation with the secondary antibody, signals were generated using the SuperLumia ECL HRP substrate kit.

### 2.7 Cell culture

The leukemic monocyte macrophage cell line (RAW 264.7 macrophage) was obtained from the American Type Culture Collection (Manassas, VA, United States). These cells were cultured in Dulbecco’s Modified Eagle Medium (DMEM) supplemented with 10% heat-inactivated fetal bovine serum (FBS) and antibiotics (100 U/mL penicillin and 100 U/mL streptomycin) at a temperature of 37°C in a humidified atmosphere containing 5% CO_2_. Routine maintenance of the cells involved sub-culturing them at a ratio of 1:3 every 2 days to ensure optimal growth and viability.

### 2.8 Validation *in vitro*

#### 2.8.1 Cell viability assay

In this experiment, cell viability was assessed using a CCK-8 kit. RAW 264.7 macrophages were seeded into the wells of a 96-well plate at a density of 3000 cells per well and incubated for 24 h to allow for attachment and initial growth. Following this incubation period, the cells were treated with various concentrations (0, 31.25, 62.5, 125, 250, 500, and 1,000 μg/mL) of TR for either 24 or 72 h. After that, 10 μL of CCK-8 solution was added to each well, and the plate was further incubated at 37°C for 1.5 h. Following the incubation with the CCK-8 solution, the absorbance was measured at 450 nm using a spectrophotometer (specifically, a Leica Microsystems spectrophotometer from Germany).

#### 2.8.2 Determination of cytokines by ELISA

Cells were initially seeded in 96-well plates at a density of 2.5 × 10^4^ cells per well and cultured in DMEM supplemented with 10% FBS for 24 h to allow for adherence and stabilization. Following this incubation period, the experimental setup consisted of different treatment groups: The blank group, which served as the control, was treated with DMEM complete culture medium. The TR groups were subjected to various concentrations of TR extract along with lipopolysaccharide (LPS) at a concentration of 100 ng/mL. The positive control group, abbreviated as the DEX group, received treatment with dexamethasone sodium phosphate injection along with LPS at a concentration of 100 ng/mL. The model group, abbreviated as the LPS group, was treated solely with LPS at a concentration of 100 ng/mL. ELISA kits were used to measure the concentrations of IL-6 and TNF-α in each well. To ensure statistical reliability, the experiment was repeated three times.

#### 2.8.3 Quantitative real-time polymerase chain reaction

RAW264.7 macrophages were seeded into a 6-well plate and interventions were performed as described in item 2.8.2 once cell adhesion was achieved. Following the treatment period, RAW264.7 macrophages were harvested from the wells and transferred into tubes. Total RNA extraction and reverse transcription of RNA was carried out according to the manufacturer’s instructions. In the qPCR system, GAPDH was employed as the internal reference gene. The reaction conditions were as follow: 95°C, 30 s; 95°C, 5 s; 58.5°C, 30 s (but gene IL-6’s annealing temperature is 64.1°C). The number of reaction cycles was set as 40. The quantification of gene expression levels was conducted using the 2^−△△Ct^ method. The primers of target genes were obtained from Chengdu Tsingke Biotech Co., Ltd. in China (Table 2).

**TABLE 2:** The sequences of primers.

Primer name	Primer sequence
GAPDH	F:5′-GGCCTTCCGTGTTCCTACC-3′
	R:5′-TGCCTGCTTCACCACCTTC-3′
COX-2	F:5′-TGAGTACCGCAAACGCTTCTC-3′
	R:5′-TGGACGAGGTTTTTCCACCAG-3′
IL-6	F:5′-TGGAGTCACAGAAGGAGTGGCTAAG-3′
	R:5′-TCTGACCACAGTGAGGAATGTCCAC-3′
TNF-α	F:5′-AGGCAACCTGACCACTCTCC-3′
	R:5′-CACCACCATCAAGGACTCAA-3′

#### 2.8.4 Western blotting

Cell culture and treatment procedures followed protocol item 2.8.3. Subsequently, RAW 264.7 macrophages were harvested and the following procedures remained consistent with those detailed in item 2.6.3.

### 2.9 Statistical analysis

Each experiment was independently conducted at least three times, and the data were reported as averages ±standard deviation. Data analysis was conducted utilizing SPSS 19.0 statistical software (Chicago, IL, United States). For parametric data, intergroup comparisons were performed using one-way analysis of variance (ANOVA), while non-parametric data were assessed using the Kruskal–Wallis H test. Multiple comparisons were carried out using the least significant difference (LSD) method and Dunnett’s T3 test. Statistical significance was considered at *p* < 0.05.

## 3 Results

### 3.1 Network pharmacology analysis

**FIGURE 1:**
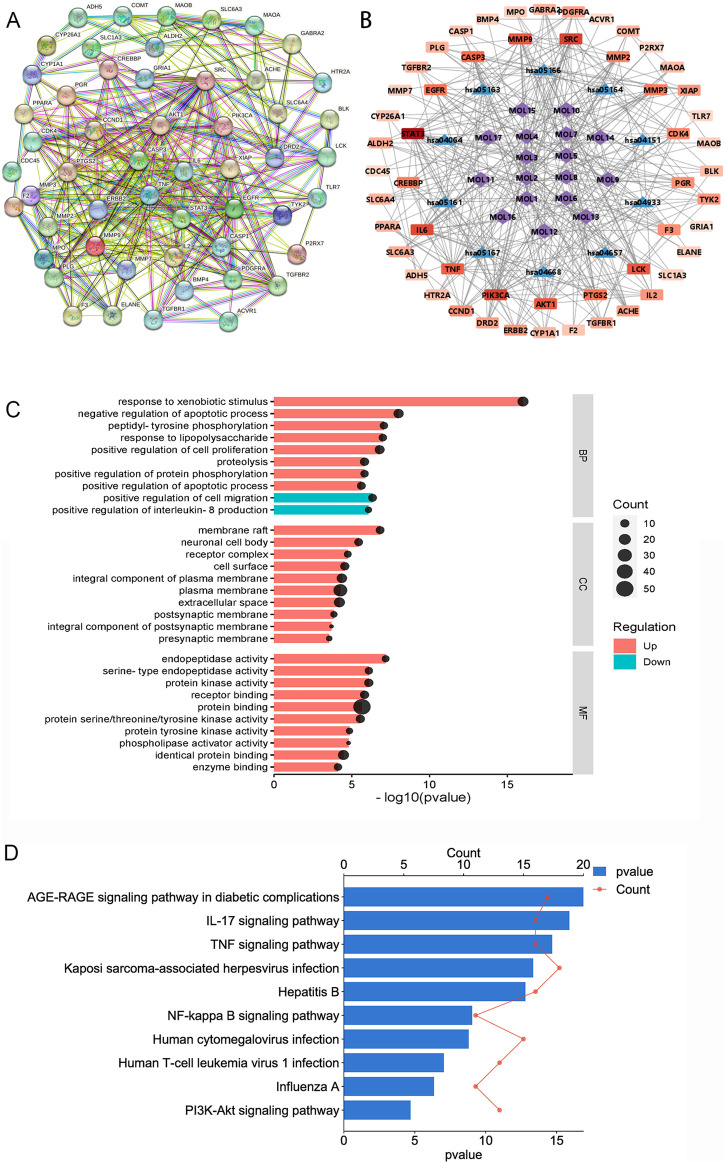
Network pharmacological analysis of the potential mechanisms of action of TR in acute pharyngitis. **(A)** PPI analysis; **(B)** GO enrichment analysis; **(C)** KEGG pathway analysis; **(D)** compound-target-pathway network. (MOL1, tetrahydropalmatine; MOL2, columbamine; MOL3, menisperine; MOL4, magnoflorine; MOL5, fibleucin; MOL6, palmatine; MOL7, jatrorrhizine; MOL8, reticuline; MOL9, neoechinulin A; MOL10, columbin; MOL11, ecdysterone; MOL12, tinoside; MOL13, 2-deoxy-20-hydroxyecdysone-3-O-glucopyranoside; MOL14, stearic acid; MOL15, palmitic acid; MOL16, 2-deoxy-20-hydroxyecdysone; MOL17, tinophylloloside. hsa04933, AGE-RAGE signaling pathway in diabetic complications; hsa04657, IL-17 signaling pathway; hsa04668, TNF signaling pathway; hsa05167, Kaposi sarcoma-associated herpesvirus infection; hsa05161, Hepatitis B; hsa04064, NF-kappa B signaling pathway; hsa05163, Human cytomegalovirus infection; hsa05166, Human T-cell leukemia virus 1 infection; hsa05164, Influenza A; hsa04151, PI3K-Akt signaling pathway).

First, we identified 545 proteins associated with 17 compounds present in TR. Subsequently, a Venn analysis was performed on a database containing 583 proteins related to acute pharyngitis and potential targets of active ingredients found in TR. This analysis revealed that 55 proteins overlapped (refer to Supplementary Table S1B). Next, we utilized the STRING database with default parameters to construct a functionally related protein-protein interaction (PPI) network (refer to Figure 1A; Supplementary Table S1C), wherein two disconnected protein targets were excluded. Following this, Gene Ontology (GO) enrichment analysis (depicted in Figure 1C; Supplementary Table S1D) indicated that TR has the potential to enhance the response to exogenous stimuli and lipopolysaccharides. It also exhibited the ability to negatively regulate IL-8 production and to modulate various cellular processes, including proliferation, apoptosis, and migration. Furthermore, TR was found to influence protein phosphorylation and hydrolysis, primarily through receptor binding and protein-protein interactions. These effects were observed across various cellular components, including the neuronal cell body, synapses, plasma membranes, and protein kinases. The KEGG pathway analysis, illustrated in Figure 1D and elaborated in Supplementary Table S1E, emphasized the participation of numerous proteins in pivotal signaling pathways. These pathways encompass AGE-RAGE, IL-17, TNF, Hepatitis B, NF-κB, Influenza A, and PI3K–Akt signaling pathways, with the majority being intricately associated with inflammation. For a more intuitive representation of the relationships among compounds, targets, and pathways, an compound–target–pathway network (illustrated in Figure 1B and documented in Supplementary Table S1F) was created, revealing the association of 17 compounds with 53 targets across 10 pathways. Notably, core protein targets such as STAT3, PIK3CA, SRC, IL6, AKT1, MMP9, TNF, CASP3, EGFR, and PTGS2/COX-2 exhibited higher degrees of Degree, Closeness Centrality, and Betweenness Centrality within the network. To sum up, TR appears to primarily function in improving acute pharyngitis by responding to various stimuli, including external factors and lipopolysaccharides, to regulate the release of inflammatory factors. Moreover, it plays a role in diverse cellular processes such as protein synthesis, signal transduction, and immunometabolic regulation.

### 3.2 TR improves acute pharyngitis in rats

**FIGURE 2:**
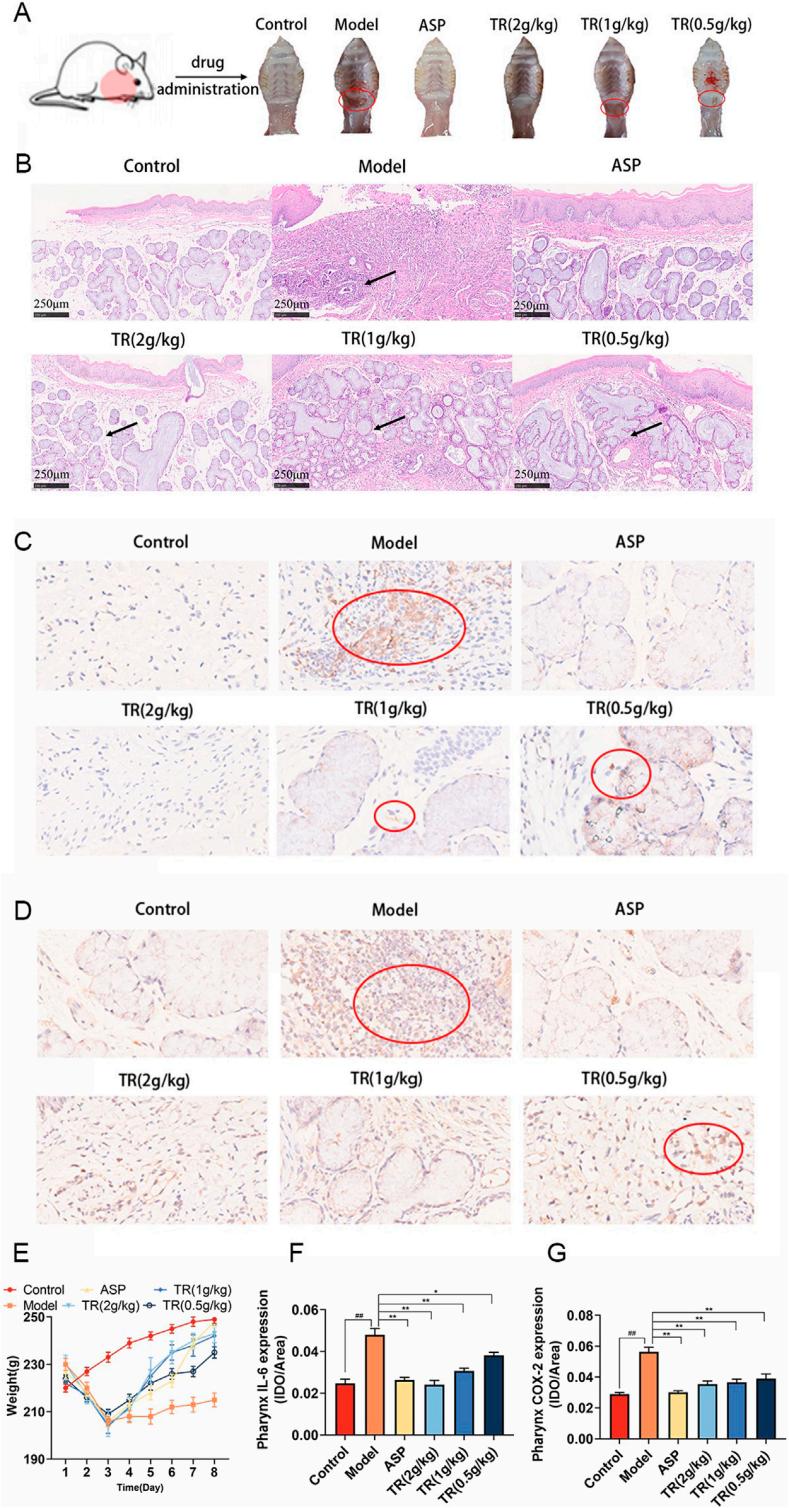
Therapeutic effects of TR on the AP rat model. **(A)** The epigenetic status of each group’s pharynx; **(B)** histological evaluation was performed by HE staining (original magnification ×10); **(C)** immunohistochemical staining (magnification, ×40) of IL-6 in the pharynx sections; **(D)** immunohistochemical staining (magnification, ×40) of COX-2 in the pharynx sections; **(E)** The weight changes of rats during modeling and dosing (n = 10); **(F)** expression level of IL-6 in the pharynx (n = 3); **(G)** expression level of COX-2 in the pharynx (n = 3). The data was expressed as the mean ± SD. ##*p* < 0.01 versus the Control group, *p < 0.05, **p < 0.01 versus the Model group.

To assess TR’s effectiveness against acute pharyngitis, we employed an ammonia-induced acute pharyngitis rat model, a well-established method widely recognized for evaluating the efficacy and mechanism of action of anti-AP drugs (Chen et al., 2020). This model involves spraying 15% ammonia onto the rats’ pharynx, inducing symptoms akin to those observed in clinical AP cases (Wang et al., 2024). The ammonia solution stimulates an increase in white blood cells, neutrophils, lymphocytes, and monocytes, augments mucus secretion, and leads to congestion and swelling of the pharyngeal mucosa. These manifestations closely mimic the clinical symptoms of AP. In general, rats exhibiting symptoms such as distraction, significantly reduced activity, diminished diet and weight, pronounced coughing, dry and lackluster fur, increased saliva secretion, and red, ulcerated oral mucosa are considered models of acute pharyngitis. Following drug administration, the severity of pharyngitis symptoms varied among the groups. Notably, the rats in the model group exhibited more severe redness, swelling, and ulceration of the pharynx compared to normal rats. However, oral treatment with ASP and TR on a daily basis alleviated the extent of redness, swelling, and ulceration of the pharynx in acute pharyngitis rats, as depicted in Figure 2A. The therapeutic efficacy of TR on AP rats was further substantiated through pathological staining of pharyngeal tissues (Figure 2B). In the control group, clear pharyngeal tissue boundaries and stratification were observed, characterized by nonkeratinized multilayer flat epithelium in the mucosal epithelium and a normal submucosal glandular structure, with a notable absence of inflammatory cells in the lamina propria. However, AP rats exhibited blurred pharyngeal boundaries and stratification, accompanied by keratinized or detached mucosal epithelium, and disruption of the morphological structure of the glands, along with significant infiltration of inflammatory cells in the lamina propria. Treatment with TR effectively ameliorated the blurred organizational boundaries, preserved glandular structures, and reduced inflammatory cell infiltration in acute pharyngitis rats, mirroring the effects observed with ASP therapy. In comparison to the control group, AP rats did not exhibit a significant increase in body weight, whereas rats in the TR group displayed a noticeable and consistent upward trend in body weight (Figure 2E). Furthermore, immunohistochemical analysis of IL-6 and COX-2 revealed an overexpression of positively stained cells in the model group, whereas the TR treatment group exhibited a significant reduction in positive cells (Figures 2C, D, F, G). These findings collectively indicate that TR effectively ameliorated pharyngitis symptoms in AP rats.

### 3.3 TR regulates metabolic profiles of AP rats and bioinformatics analysis

**FIGURE 3:**
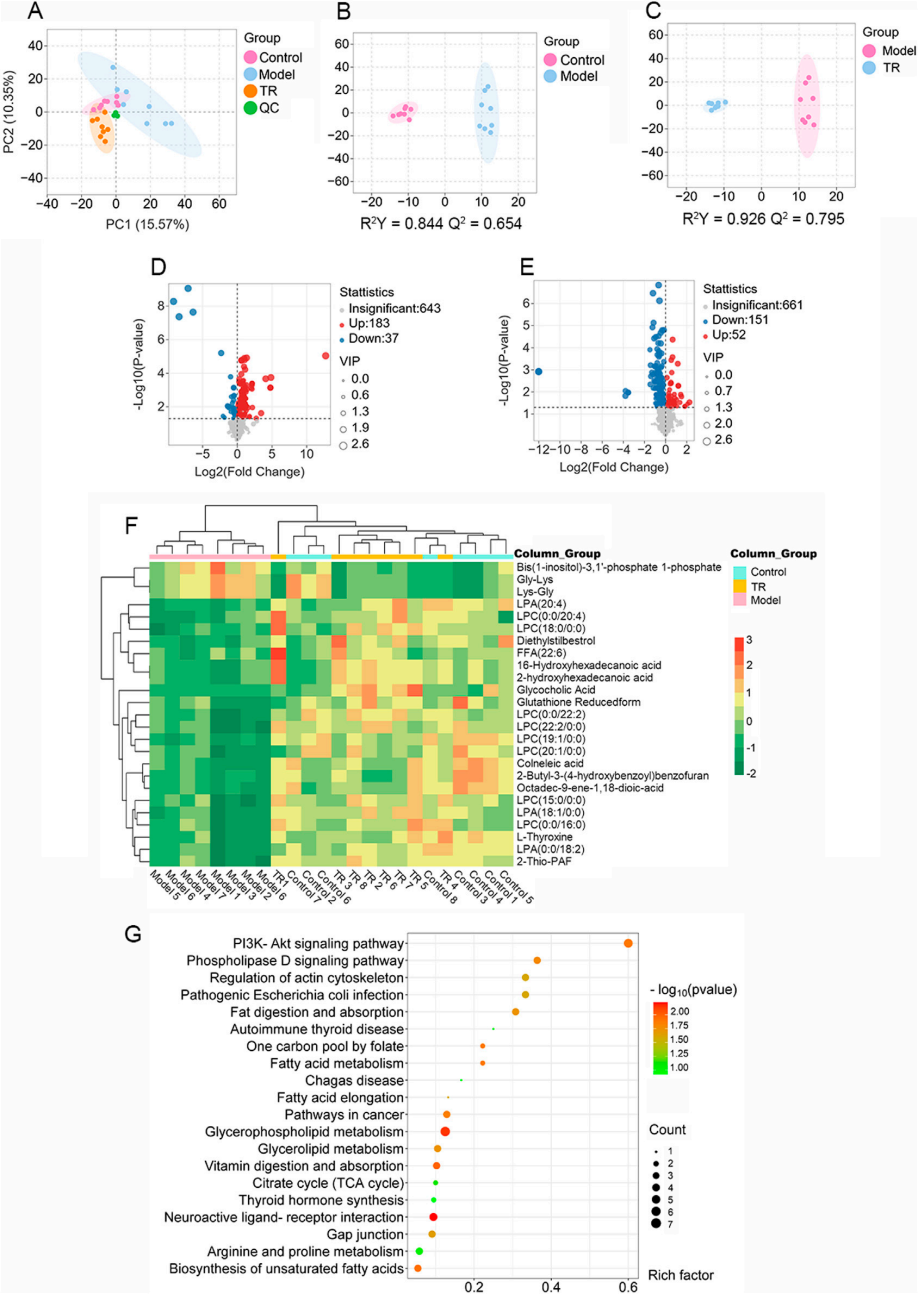
Serum metabolomics analysis of rats with acute pharyngitis. **(A–D)** PCA and OPLS-DA analysis for discriminating the metabolic signatures. **(A)** PCA analysis for three groups. **(B)** OPLS-DA analysis for the control group and model group. **(C)** OPLS-DA analysis for the TR group and model group. **(D)** differential metabolite volcano plot for Normal versus Model groups. **(E)** differential metabolite volcano plot for TR versus Model groups. **(F)** heatmap of the 25 most discriminating metabolites. **(G)** Metabolic pathway analysis of potential biomarkers.

To investigate the potential pharmacological activity of TR against acute pharyngitis, serum metabolomics analysis was conducted across control, model, and TR groups. The total ion chromatogram (TIC) of serum samples is illustrated in Supplementary Figure S1. Additionally, validation through peak shift, retention time, and peak area of quality control (QC) samples demonstrated the robust stability and reproducibility of the established method. In total, 864 metabolites were identified after integrating all metabolites detected in both positive and negative ion modes. PCA and OPLS-DA were employed to pinpoint the distinctive metabolites contributing to the differentiation among the three groups: control, model, and TR groups. As depicted in Figures 3A–C, the score plot exhibited a distinct separation trend between the control group and the model group, suggesting significant variations in the serum metabolic profiles of AP rats compared to normal ones. In comparison to the model group, the TR group displayed a distinct trajectory. The OPLS-DA models established exhibited high cross-validation parameter values (control group versus model group: R^2^Y = 0.844, Q^2^ = 0.654; TR group versus model group: R^2^Y = 0.926, Q^2^ = 0.795), signifying satisfactory interpretation of variance and predictive capability of the model. These results suggest that the established model could effectively identify potential biomarkers for further experimentation.

For precise identification, potential differential metabolites were chosen based on the principle that VIP ≥1.0, and *p*-value <0.05 between the control and model groups. Analysis of the volcano plot of differential metabolites (Figures 3D, E) and data comparison revealed that the majority of metabolites in the control and TR groups were downregulated compared to the model group, suggesting a convergence between the TR and control groups. As a result, a total of 126 endogenous metabolites with notable alterations were identified (refer to Supplementary Table S2A). Of these, 101 exhibited downregulation in the model group, including LPC 16:0, LPC 20:4, LPA 18:1, and LPA 20:4. Conversely, 25 metabolites showed upregulation, among which were Glycine-Lysine and L-Lysine-L-Glycine, etc. A subset of 25 metabolites, representing these alterations, was visualized in the heatmap (Figure 3F; Supplementary Table S2B). Lysophosphatidylcholine (LPC) and lysophosphatidic acid (LPA) are both lysophospholipids. The numbers following LPC/LPA denote the number of carbon atoms and double bonds in the fatty acid chain. For example, LPC 16:0 refers to lysophosphatidylcholine with a saturated fatty acid chain containing 16 carbon atoms. LPC and LPA play critical roles in glycerophospholipid metabolism by serving as intermediates, signaling molecules, and regulators of enzyme activity (Ren et al., 2022; Yanagida and Shimizu, 2023). Dysregulation of LPC and LPA metabolism has been implicated in various diseases, including cancer, inflammation, cardiovascular disorders, and neurological conditions (Plastira et al., 2016; Liu et al., 2023; Zhang et al., 2023a). To elucidate the mechanism behind TR’s efficacy in treating acute pharyngitis, we utilized MetaboAnalyst to construct metabolic pathways by integrating potential differential metabolites. From this analysis, we identified a total of 71 pathways. Notably, the top 20 pathways, as illustrated in Figure 3G, showcased significant -log (*p*-value) scores and Rich factors for the PI3K-Akt signaling pathway. This observation suggests a pivotal role for this pathway in reflecting alterations in potential metabolites. Remarkably, these findings align with those derived from network pharmacological analysis, further reinforcing the significance of the PI3K-Akt signaling pathway in the therapeutic mechanism of TR against acute pharyngitis. Moreover, the pathway analysis highlighted glycerophospholipid metabolism as a potentially enriched pathway, closely intertwined with the PI3K-Akt signaling pathway. This connection primarily arises from their pivotal roles in governing cellular signaling, membrane dynamics, and metabolic processes (Naguib, 2016; Cao et al., 2021).

### 3.4 Common mechanistic analysis integrating network pharmacology and metabolomics

**FIGURE 4:**
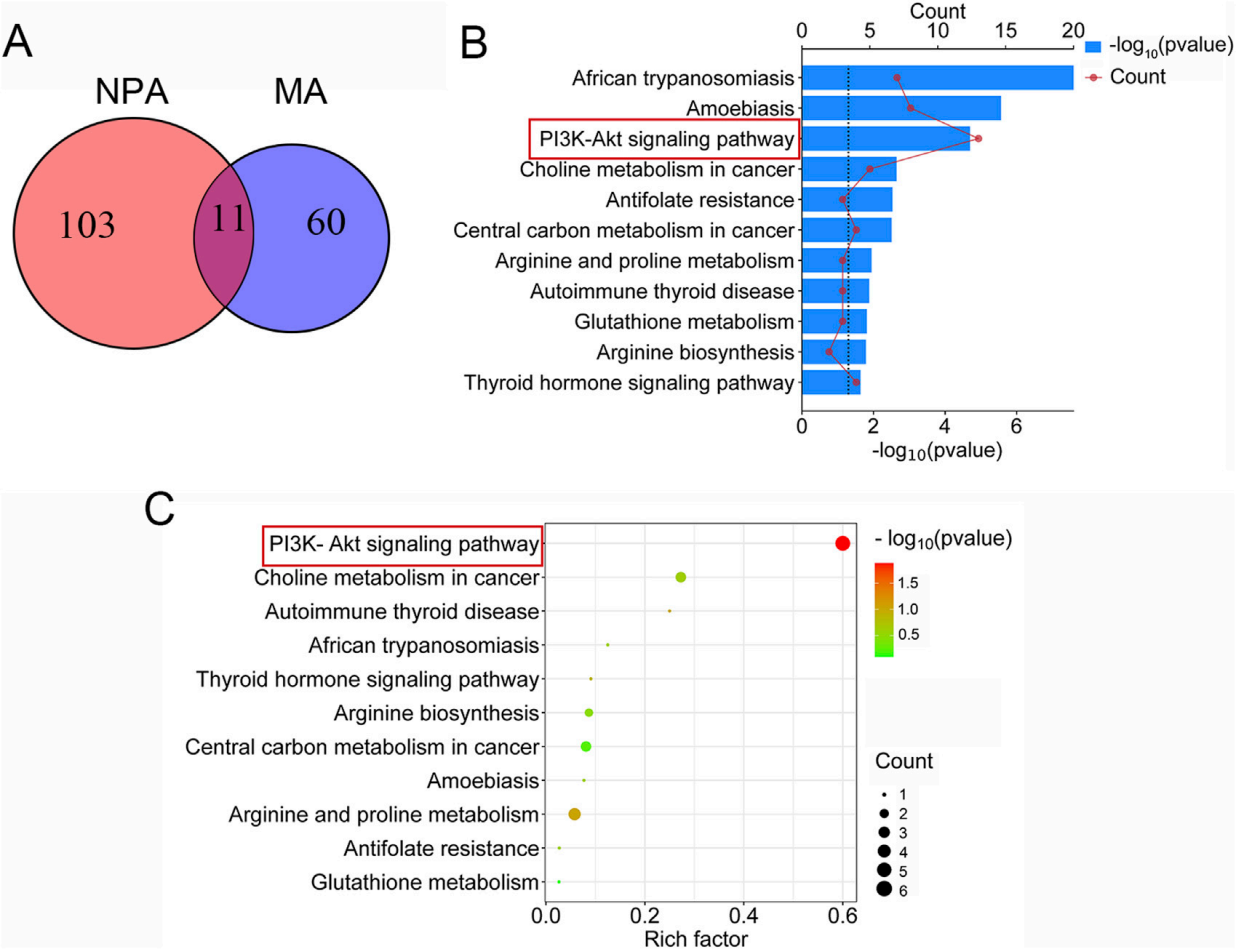
Common mechanistic analysis integrating network pharmacology analysis (NPA) and metabolomics analysis (MA). **(A)** A total of 114 pathways were enriched in KEGG analysis of network pharmacology, 71 metabolic pathways were selected in metabolomics, and 11 signaling pathways coincided. **(B)** The rank of 11 intersection pathways in network pharmacology. **(C)** The rank of 11 intersection pathways in metabolomics.

In the KEGG enrichment analysis of network pharmacology, a total of 114 signaling pathways associated with acute pharyngitis were identified. Concurrently, metabolomics analysis revealed enrichment in 71 metabolic pathways. Through the comparison using a Venn diagram, it was observed that 11 pathways intersected between the two groups (Figure 4A). Subsequently, these intersecting pathways were evaluated using pathway enrichment indices from both network pharmacology and metabolomics. Remarkably, the PI3K-Akt pathway emerged as highly enriched according to the ranking of indices from both methods, suggesting its potential role as a target pathway for anti-acute pharyngitis (AP) action of TR (Figures 4B, C). In addition, both network pharmacology and metabolomics indicate that TR regulates inflammatory factors in response to external stimuli/LPS stimuli, thereby combating the inflammatory response associated with acute pharyngitis. As a result, additional *in vitro* and *in vivo* experiments were designed to delve into the implications of the PI3K-Akt pathway and inflammatory factors.

### 3.5 Effect of TR on inflammatory factors in acute pharyngitis rats

**FIGURE 5:**
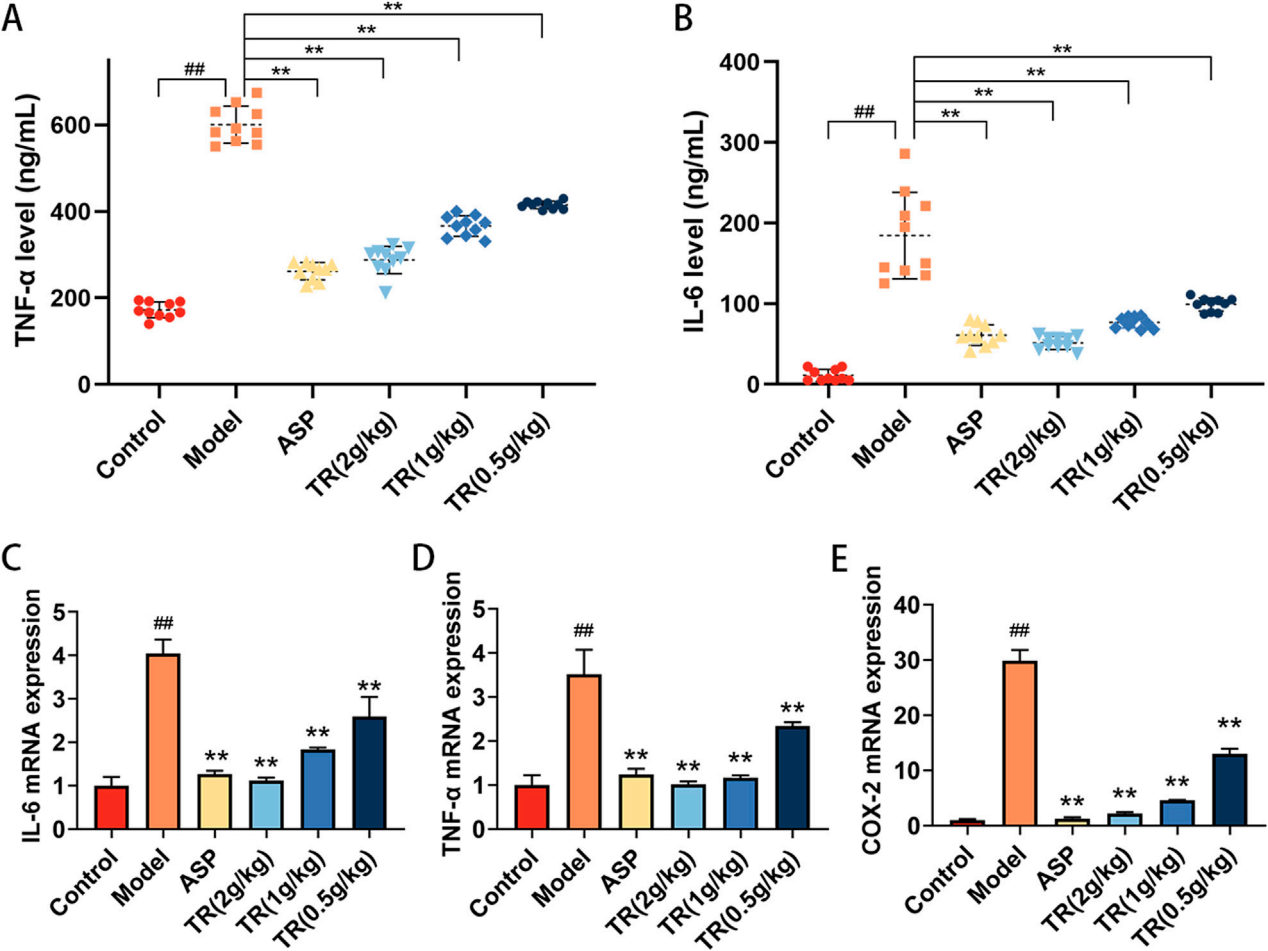
TR regulated inflammatory cytokines in AP rats. **(A)** Serum levels of TNF-α were detected by ELISA (n = 10). **(B)** Serum levels of IL-6 (n = 10). **(C)** The mRNA levels of IL-6 in the pharynx were detected by RT-PCR (n = 3). **(D)** The mRNA levels of TNF-α in the pharynx (n = 3). **(E)** The mRNA levels of COX-2 in the pharynx (n = 3). Data was presented as mean ± SD. Statistic difference is indicated as ##*p* < 0.01 versus the Control group, *p < 0.05, **p < 0.01 versus the Model group.

In order to assess the impact of TR on the inflammatory response in AP rats, inflammatory cytokines closely associated with acute pharyngitis, such as TNF-α and IL-6, were measured. The serum levels of TNF-α and IL-6 were found to be elevated in AP rats compared to the control group (Figures 5A, B). However, treatment with TR and ASP led to a reduction in the levels of these inflammatory factors. Subsequently, the mRNA expressions of IL-6, TNF-α, and COX-2 were evaluated in the pharyngeal tissue of acute pharyngitis rats. Similarly, a notable decrease was observed in the mRNA levels of IL-6, TNF-α, and COX-2 in both the ASP and TR treatment groups (Figures 5C–E). These findings suggest that TR exhibits anti-inflammatory effects in AP rats.

### 3.6 TR regulated PI3K-Akt signaling pathway in AP rats

**FIGURE 6:**
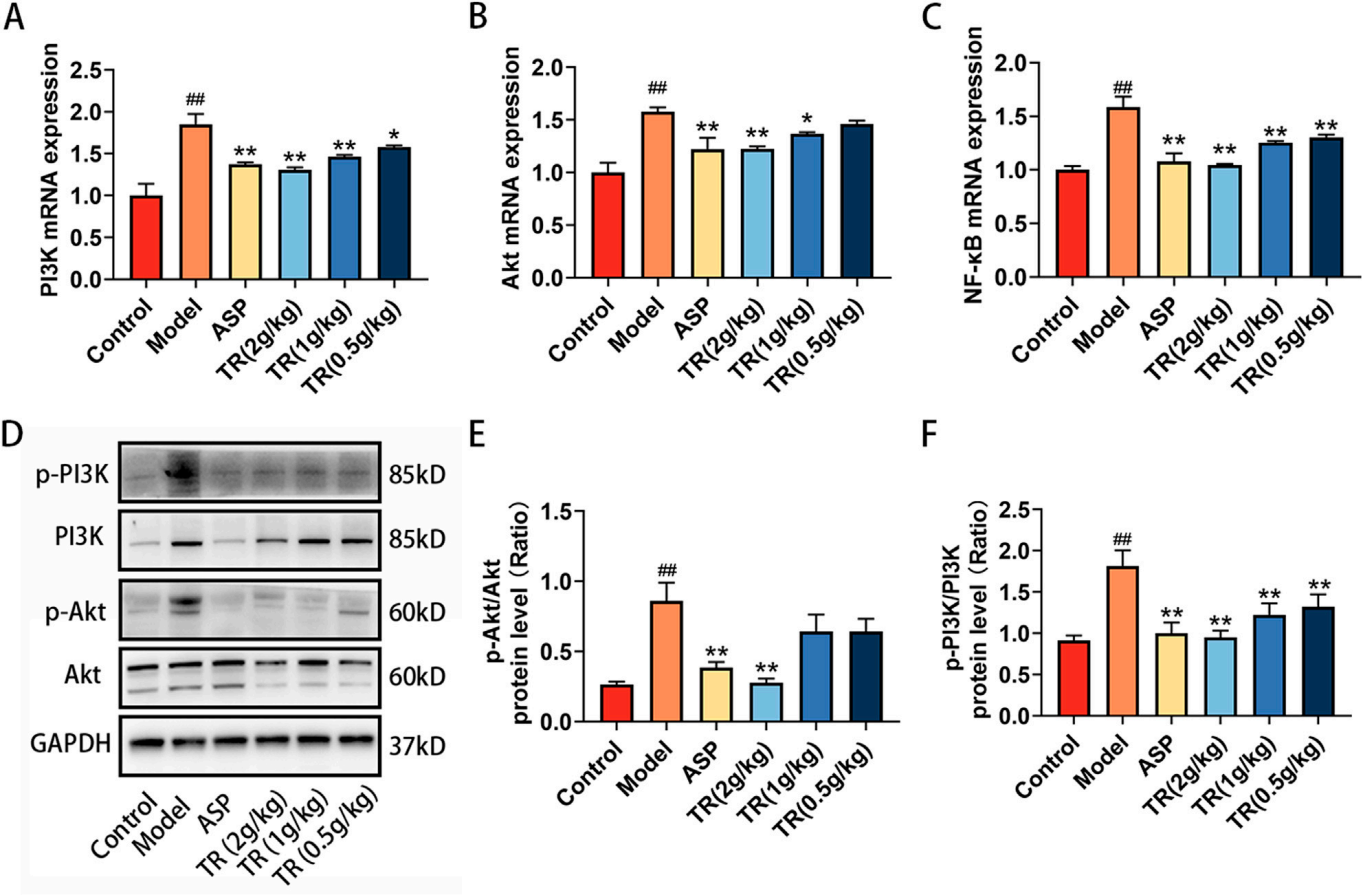
TR regulating PI3K-Akt signaling pathways in AP rats. **(A)** The mRNA levels of PI3K in pharynx were detected by RT-PCR (n = 3). **(B)** The mRNA levels of Akt in pharynx (n = 3) **(C)** The mRNA levels of NF-κB in pharynx (n = 3) **(D–F)** Phosphorylated PI3K and phosphorylated Akt protein levels in pharynx were detected by Western blotting. The data was expressed as the mean ± SD. ##*p* < 0.01 versus the Control group, *p < 0.05, **p < 0.01 versus the Model group.

The alterations in inflammatory factor expression suggested that TR effectively suppressed the inflammatory response. As indicated by the results from network pharmacology and metabolomics, the PI3K-Akt signaling pathway emerged as a potential target pathway for TR in alleviating AP. To delve into TR’s impact on this pathway, the expressions of PI3K, Akt, NF-κB, p-PI3K, and p-Akt were assessed via RT-PCR and WB analyses. As illustrated in Figures 6A–C, TR notably decreased the mRNA expressions of PI3K, Akt, and NF-κB in the pharyngeal tissue of acute pharyngitis rats. Subsequently, protein expression changes were assessed in the pharyngeal tissue of AP rats. Compared to the model group, TR significantly reduced the protein expressions of phosphorylated PI3K and phosphorylated Akt in acute pharyngitis rats (Figures 6D–F), indicating TR’s role in attenuating the inflammatory response by inhibiting the PI3K-Akt pathway.

### 3.7 Effects of TR on RAW264.7 cell viability

**FIGURE 7:**
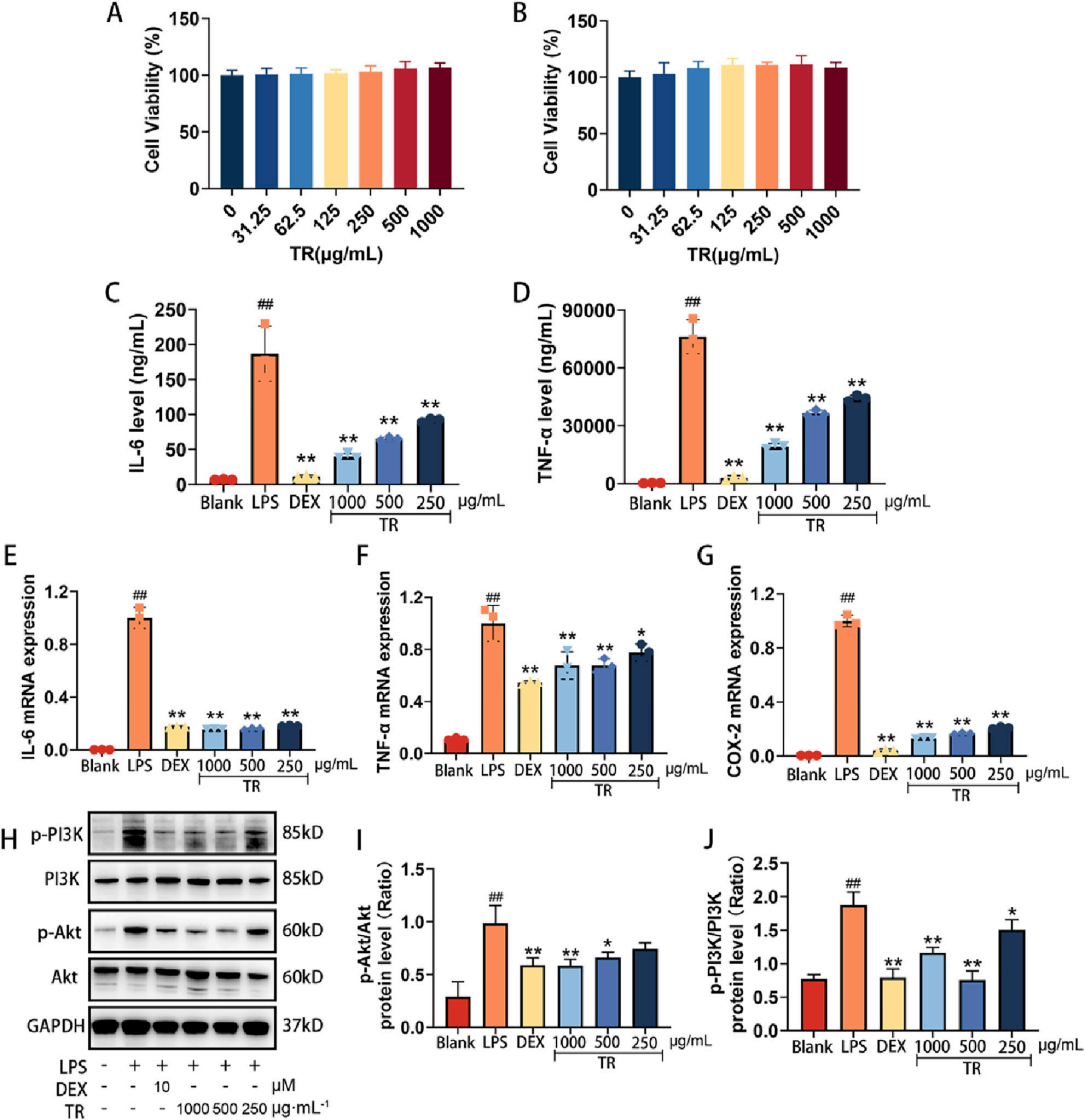
Effects of the TR extract on inflammatory factors and PI3K-Akt signaling pathway in LPS-induced RAW264.7 macrophages. **(A)** Effect of TR on LPS-induced macrophage cell activity for 24 h. **(B)** Effect of TR on LPS-induced macrophage cell activity for 72 h. **(C, D)** Effects of TR on the secretion of IL-6 and TNF-α in RAW264.7 cells induced by LPS (n = 3). **(E–G)** The mRNA levels of IL-6, TNF-α, and COX-2 (n = 3). **(H–J)** The phosphorylation of PI3K and Akt was detected via Western blotting. The data was expressed as the mean ± SD of three independent experiments. ##*p* < 0.01 versus the Blank group, *p < 0.05, **p < 0.01 versus the LPS group.

Our investigation revealed that TR primarily mitigated acute pharyngitis by modulating the inflammatory response *in vivo*. Consequently, for *in vitro* validation, we opted for the LPS-induced RAW264.7 inflammation model. As depicted in Figures 7A, B, following a 24-h and 72-h incubation of RAW 264.7 macrophages with varying concentrations of TR, no significant alteration in cell viability was observed within the range of 0–1,000 μg/mL. Notably, certain doses of the TR extract even enhanced macrophage viability. Hence, we selected concentrations of 250, 500, and 1,000 μg/mL of TR extraction solution for subsequent experiments.

### 3.8 Effect of TR on inflammatory factors in RAW264.7 cell

To initially assess the anti-inflammatory potential of the TR extract *in vitro*, we examined the release of IL-6 and TNF-α, along with the expression of IL-6, TNF-α, and COX-2 in LPS-induced RAW264.7 cells. In comparison to untreated cells, LPS significantly elevated the release of IL-6 and TNF-α (Figures 7C, D) in macrophages and induced the expression of IL-6, TNF-α, and COX-2 (Figures 7E–G). However, the levels of TNF-α and IL-6 in the cell culture supernatant markedly decreased (*p* < 0.05) in the TR groups compared to the LPS group. Furthermore, the mRNA expression of TNF-α, IL-6, and COX-2 exhibited a remarkable reduction (*p* < 0.01) in the TR groups compared to the LPS group, with the most significant decrease observed in COX-2 mRNA expression.

### 3.9 TR regulated PI3K-Akt signaling pathway in LPS-induced RAW264.7 cell

The PI3K-Akt signaling pathway plays a pivotal role in regulating various cellular processes such as proliferation, differentiation, and apoptosis. It is closely implicated in inflammation, tumorigenesis, and disorders of the reproductive system. To further elucidate the anti-acute pharyngitis (AP) mechanism of TR, we investigated the impact of the TR extract on key proteins within the PI3K-Akt signaling pathway. Following a 24-h stimulation with LPS, macrophages exhibited increased phosphorylation of PI3K and Akt. However, the TR extract notably attenuated the phosphorylation of PI3K and Akt (Figures 7H–J). These findings suggest that the PI3K-Akt signaling cascade might represent a potential pathway targeted by the TR extract in its anti-AP effects.

## 4 Discussion

Acute pharyngitis is characterized by its high incidence rate and multifaceted pathogenesis (Krüger et al., 2021). Currently, there are no specific drugs tailored for the treatment of acute pharyngitis (Krüger et al., 2021). Antibiotics are predominantly utilized to forestall complications and secondary infections in cases of bacterial pharyngitis (Meskina and Stashko, 2020). However, the burgeoning resistance and potential toxic side effects of antibiotics, particularly in pediatric patients, significantly impede their clinical efficacy (Bamford et al., 2024). Notably, Xiaoer Yanbian granule, a composite formulation containing Tinosporae Radix, has demonstrated efficacy in treating acute pharyngitis and tonsillitis in children (Zhang et al., 2019a; Zhang et al., 2022; Shen, 2023; Niu, 2024). Hence, this study delves into elucidating the protective effects of TR against acute pharyngitis.

To comprehensively elucidate the pharmacological mechanisms underlying TR’s protective effects against acute pharyngitis (AP), we employed a combination of network pharmacology methodologies and metabolomics techniques for investigation and analysis. Subsequently, we validated these findings through both *in vitro* and *in vivo* experiments. Our initial exploration revealed that TR treatment for acute pharyngitis engages targets such as PIK3CA, IL6, AKT1, TNF, PTGS2/COX-2, among others. As per literature, IL-6 belongs to a class of cytokines generated by activated monocyte-macrophages and lymphocytes. It plays a crucial role in various stages of the inflammatory response, primarily by stimulating the proliferation and differentiation of immune cells (Del Giudice and Gangestad, 2018; Kang et al., 2020). Consequently, it actively participates in the body’s immune response, thereby augmenting the inflammatory cascade (Zhu et al., 2018). TNF-α is intricately involved in multiple signaling pathways of inflammation and exerts regulatory control over the synthesis and release of various cytokines (Saha and Smith, 2018). Pertinent research has identified PTGS2 as a pivotal inducible enzyme catalyzing prostaglandin synthesis during inflammation (Yang et al., 2023). Its expression is upregulated, and it actively participates in inflammatory responses following inflammatory stimulation and tissue damage. Additionally, PI3K plays a significant role in the migratory process of inflammatory cells, while Akt, upon activation, modulates NF-κB through a complex mechanism (Fang et al., 2022). Subsequently, activated NF-κB orchestrates the transcription of numerous proteins crucially involved in inflammatory responses (Gustin et al., 2004). Many traditional Chinese medicine formulations exert their therapeutic effects in treating pharyngitis by suppressing the release of inflammatory factors, including IL-6, IL-1β, PGE2, and TNF-α, etc (Kong et al., 2018; Liu et al., 2019; Wu et al., 2021). For instance, resveratrol has been demonstrated to prevent inflammation induced by acute pharyngitis through the inhibition of NF-κB. Similarly, the YHQ formula has been shown to alleviate pharyngitis-related symptoms by suppressing COX-2 and phosphorylation of p38 MAPK, Erk, and NF-κB (p65) (Xu et al., 2020). This study employed both bioinformatics analysis and experimental validation to demonstrate that TR augments its response to exogenous and LPS stimuli by regulating the expression and release of inflammatory factors in the context of AP. Through KEGG analysis, it was revealed that TR’s anti-AP pathway primarily encompasses signaling pathways such as IL-17, TNF, Hepatitis B, and PI3K-Akt signaling pathway, among others. Notably, the PI3K-Akt signaling pathway plays a pivotal role in orchestrating cellular responses to extracellular stimuli and is closely associated with inflammation, tumorigenesis, and disorders of the reproductive system (Acosta-Martinez and Cabail, 2022; He et al., 2022). Inhibiting the activation of the PI3K-Akt signaling pathway and the release of upstream and downstream modulators holds promise for mitigating inflammatory pain and neuropathic pain (Xu et al., 2011). According to reports, the PI3K-Akt signaling pathway emerges as one of the potential mechanisms underlying the efficacy of Kaihoujian Throat Spray (children’s type) against acute pharyngitis and tonsillitis, as determined through proteomic analysis and ELISA (Pang et al., 2023). Furthermore, Hosta plantaginea (Lam.) Aschers flowers have been shown to mitigate pharyngeal injury in rats by suppressing inflammation, achieved through the inhibition of various pathways including PI3K-Akt, MAPKs, JAK-STAT, and NF-κB (Wang et al., 2024). Similarly, the present study corroborates these findings by demonstrating that TR alleviates the inflammatory response in rats with acute pharyngitis by targeting the PI3K-Akt signaling pathway, thereby exerting a therapeutic effect on AP.

**FIGURE 8:**
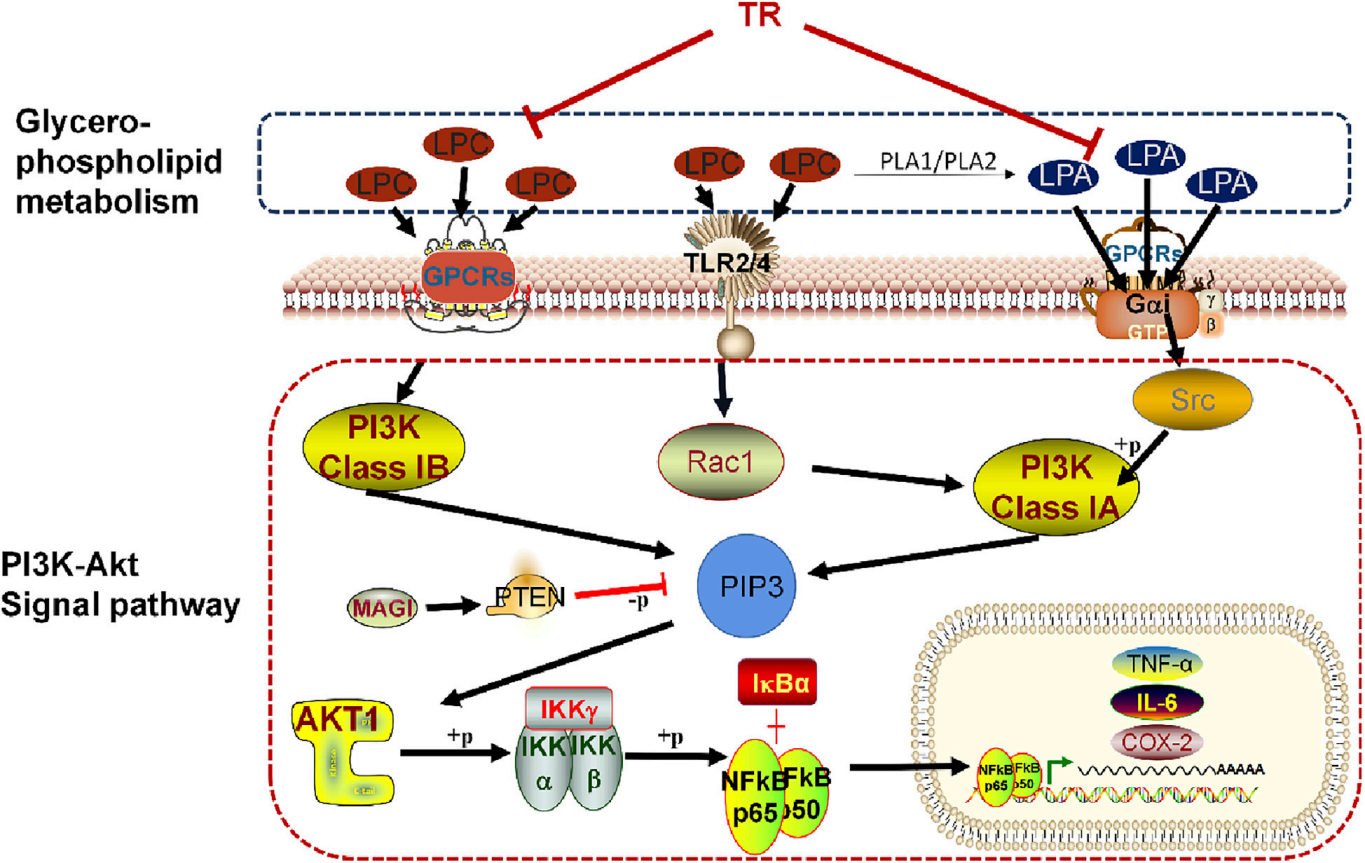
Mechanism of TR in combating acute pharyngitis.

Furthermore, our investigation revealed that TR may modulate lysophosphatidylcholine (LPC) and lysophosphatidic acid (LPA) within the glycerophospholipid metabolism pathway by regulating the PI3K-Akt pathway. This regulatory mechanism contributes to ameliorating the inflammatory response and ultimately addressing acute pharyngitis. A detailed depiction of this specific mechanism is presented in Figure 8. LPC has been implicated in various inflammatory processes, including the migration of lymphocytes and macrophages, heightened production of pro-inflammatory cytokines, induction of oxidative stress, and facilitation of apoptosis. These effects contribute to the exacerbation of inflammation and the progression of associated diseases (Liu et al., 2020). Research findings suggest that LPC may detrimentally affect ileum mucosal cells, exacerbating colonic inflammation, and causing epithelial barrier damage in Fut2△IEC mice (Tang et al., 2021). The mechanism through which LPC induces inflammatory factors often involves the PI3K/Akt signaling pathways (Chang et al., 2017). LPC may indirectly influence PI3K activation through receptor-mediated signaling or by modulating downstream components of the pathway. However, LPA can even directly activate PI3K. Upon binding to its receptors, LPA triggers the activation of PI3K, which in turn phosphorylates phosphatidylinositol 4,5-bisphosphate (PIP2) to generate phosphatidylinositol 3,4,5-trisphosphate (PIP3). LPA1 receptors initiate signaling cascades involving MAPK, PLC, Akt, and Rho pathways, culminating in a spectrum of physiological responses such as cell proliferation, survival, migration, and Ca^2+^ mobilization (Ishii et al., 2004). Additionally, LPA2 receptors, upon activation, drive cell survival, migration, and altered gene expression through pathways including Ras, Rac, PI3K, and MAPK (Contos et al., 2000). The PI3K/Akt pathway, crucial for orchestrating morphological changes, cell migration, survival, and Adenylyl cyclase inhibition, is activated by Gαi/o, the receptor of LPA (Kranenburg and Moolenaar, 2001). Importantly, findings from Lisha Joshi’s research suggest that LPA5 receptors exacerbate neuroinflammation by transmitting pro-inflammatory signals during endotoxemia, primarily through microglial activation induced by LPA (Joshi et al., 2022). Overall, LPC and LPA can influence the PI3K-Akt pathway through receptor-mediated signaling, contributing to the regulation of cellular homeostasis and pathophysiological processes, ultimately alleviating acute pharyngitis (Lei et al., 2019; Pei et al., 2022).

We propose that TCM treatment, specifically TR, may offer benefits in managing acute pharyngitis. While our study yielded significant findings, we acknowledge its limitations. We clarified that TR potentially alleviates acute pharyngitis by modulating the PI3K-Akt pathway and reducing levels of lysophospholipids like LPC and LPA. Additionally, it inhibits key targets such as IL-6, TNF-α, and COX-2 to mitigate the inflammatory response. However, given TCM’s multi-pathway and multi-component nature, we cannot exclude other potential mechanisms of action. Therefore, a more comprehensive investigation into TR’s mechanism is warranted. By employing a combination of network pharmacology and metabolomic analysis, we discovered that TR might exert effects on additional biological functions including synapse, receptor, and signal transduction modulation (Zhang et al., 2019b). Furthermore, it appears to influence neuroactive ligand-receptor interactions and autoimmune pathways (Pisetsky, 2023). Extensive research indicates that medications can modulate neuronal or synaptic function (Yan et al., 2022) via the PI3K/Akt signaling pathway, thereby influencing the nervous system and potentially treating neurological disorders like depression and Alzheimer’s disease (Yang et al., 2020; Li et al., 2021). Furthermore, recent studies indicate a close relationship between the PI3K-Akt signaling pathway and human immune responses (Na et al., 2020). While traditionally recognized for its role in regulating adaptive immune cell activation (Pan et al., 2022), it’s now increasingly evident that the PI3K-Akt pathway also plays significant and distinct roles in innate immune cells (Weichhart and Säemann, 2008). Hence, future investigations could delve into signal transduction functions and autoimmune responses to provide a more comprehensive understanding of TR’s mechanism in combating acute pharyngitis. In addition, the material basis for the anti-pharyngitis properties of TR remains to be studied. Current research indicates that two classes of compounds in TR, namely, diterpene lactones and alkaloids (Liu et al., 2010; Song et al., 2023), exhibit good anti-inflammatory efficacy. However, it is still unclear whether these two classes of compounds can improve pharyngitis, or if other compounds play a role in combating the condition. These questions require further exploration. At the same time, research on its toxicological properties is limited, and safety assessments are insufficient, which severely hinders its clinical application. Therefore, it is essential to strengthen toxicological studies.

In summary, our study illustrates that TR effectively alleviates the physiological, pathological, and biochemical alterations associated with acute pharyngitis, showcasing notable anti-AP effects. Leveraging two primary methodologies—network pharmacology and metabolomics—we elucidated TR’s mechanism of action, revealing its impact on the PI3K-Akt signaling pathway and glycerophospholipid metabolism. Through both *in vitro* and *in vivo* experiments, we establish the pivotal role of the PI3K-Akt pathway in mitigating inflammatory responses during TR treatment of acute pharyngitis. These findings lay a solid foundation for the potential development of TR as a novel clinical intervention for AP.

## Data Availability

The datasets presented in this study can be found in online repositories. The names of the repository/repositories and accession number(s) can be found in the article/Supplementary Material.

## Abbreviations

ASP, aspirin; ANOVA, one-way analysis of variance; AP, acute pharyngitis; CMC-Na, sodium carboxymethyl cellulose; DEX, dexamethasone; FBS, fetal bovine serum; LPA, lysophosphatidic acid; LPC, lysophosphatidylcholine; LSD, least significant difference method; NF-κB, nuclear factor kappa B; NSAIAs, nonsteroidal anti-inflammatory agents; OPLS-DA, orthogonal partial least squares discriminant analysis; PCA, principal component analysis; PPI, protein-protein interaction; QTRAP, triple quadrupole-linear ion trap mass spectrometer; RT-qPCR, quantitative real-time polymerase chain reaction; TIC, total ion chromatogram; TR, Tinosporae Radix.
