# Supplementary material for: Tinosporae Radix attenuates acute pharyngitis by regulating glycerophospholipid metabolism and inflammatory responses through PI3K-Akt signaling pathway
Source: Front Pharmacol. 2024 Nov 6;15:1491321. doi: 10.3389/fphar.2024.1491321 (PMC11576305; doi:10.3389/fphar.2024.1491321)
Supplement: Supplementary file 1 [file Table1.docx]

**The Content of Supplementary Tables**

[Table S1A The ADME parameters of 17 compounds](#_Toc160976934)

[Table S1B Venn analysis on targets associated with 17 compounds and proteins related to acute pharyngitis](#_Toc160976935)

[Table S1C PPI network](#_Toc160976936)

[Table S1D GO enrichment analysis](#_Toc160976937)

[Table S1E KEGG pathway analysis](#_Toc160976938)

[Table S1F Compound-target-pathway network analysis](#_Toc160976939)

[Table S2A All differential metabolites](#_Toc160976940)

[Table S2B The information of 25 key metabolites](#_Toc160976941)

[Table S2C KEGG pathway analysis](#_Toc160976942)

[Table S2D The enrichment level of Metabolic pathways](#_Toc160976943)

[Table S3A Intersection pathways of network pharmacology analysis and metabolomics analysis](#_Toc160976944)

[Table S3B The rank of 11 intersection pathways in network pharmacology](#_Toc160976945)

[Table S3C The rank of 11 intersection pathways in metabolomics](#_Toc160976946)

# Table S1A The ADME parameters of 17 compounds

| **Molecule number** | **name** | **Formula** | **MW** | **Rotatable bonds** | **H-bond acceptors** | **H-bond donors** | **Consensus Log P** | **GI absorp**  **-tion** | **Lipinski violations** | **Ghose violations** | **Veber violations** | **Egan violations** | **Muegge violations** |
| --- | --- | --- | --- | --- | --- | --- | --- | --- | --- | --- | --- | --- | --- |
| MOL 1 | (±) Tetrahydro  palmatine | C21H25NO4 | 355.43 | 4 | 5 | 0 | 3.08 | High | 0 | 0 | 0 | 0 | 0 |
| MOL 2 | columbamine | C20H20NO4 | 338.38 | 3 | 4 | 1 | 2.33 | High | 0 | 0 | 0 | 0 | 0 |
| MOL 3 | menisperine | C21H26NO4 | 356.44 | 3 | 4 | 1 | 1.43 | High | 0 | 0 | 0 | 0 | 0 |
| MOL 4 | magnoflorine | C20H24NO4 | 342.41 | 2 | 4 | 2 | 1.14 | High | 0 | 0 | 0 | 0 | 0 |
| MOL 5 | fibleucin | C20H20O6 | 356.37 | 1 | 6 | 1 | 2.1 | High | 0 | 0 | 0 | 0 | 0 |
| MOL 6 | palmatine | C21H22NO4 | 352.4 | 4 | 4 | 0 | 2.64 | High | 0 | 0 | 0 | 0 | 0 |
| MOL 7 | jatrorrhizine | C20H20NO4 | 338.38 | 3 | 4 | 1 | 2.31 | High | 0 | 0 | 0 | 0 | 0 |
| MOL 8 | reticuline | C19H23NO4 | 329.39 | 4 | 5 | 2 | 2.64 | High | 0 | 0 | 0 | 0 | 0 |
| MOL 9 | neoechinulin A | C19H21N3O2 | 323.39 | 3 | 2 | 3 | 2.63 | High | 0 | 0 | 0 | 0 | 0 |
| MOL 10 | columbin | C20H22O6 | 358.39 | 1 | 6 | 1 | 2.12 | High | 0 | 0 | 0 | 0 | 0 |
| MOL 11 | ecdysterone | C27H44O7 | 480.63 | 5 | 7 | 4 | 1.74 | High | 0 | 1 | 0 | 0 | 0 |
| MOL 12 | tinoside | C25H30O10 | 490.53 | 4 | 8 | 4 | 0.15 | High | 0 | 0 | 0 | 1 | 0 |
| MOL 13 | 1. deoxy-20β-   hydroxyecdysone-3-O-glucopyranoside | C26H42O5 | 436.78 | 8 | 8 | 3 | 0.98 | High | 0 | 0 | 0 | 0 | 1 |
| MOL 14 | Stearic acid | C18H36O2 | 284.48 | 16 | 2 | 1 | 5.93 | High | 1 | 0 | 0 | 0 | 1 |
| MOL 15 | Palmitic acid | C16H32O2 | 256.42 | 9 | 2 | 1 | 5.2 | High | 0 | 0 | 1 | 0 | 1 |
| MOL 16 | 2-Deoxy-20-  hydroxyecdysone | C27H44O6 | 464.63 | 5 | 6 | 5 | 2.58 | High | 0 | 1 | 0 | 0 | 0 |
| MOL 17 | tinophylloloside | C26H34O9 | 490.57 | 6 | 9 | 4 | 0.99 | High | 0 | 0 | 0 | 0 | 0 |

Notes: The Druglikeness rule includes five rules that examine drug class properties, including the Lipinski rule (the Five Principles of Drug Class), the Ghost rule, the Veber rule, the Egan rule, and the Muage rule. It is believed that drugs that do not violate three of the above five rules (i.e., the number is 0) and have high GI absorption in the gastrointestinal tract have drug class properties.

# Table S1B Venn analysis on targets associated with 17 compounds and proteins related to acute pharyngitis

| **targets associated with**  **17 compounds** | **targets related to**  **acute pharyngitis** | **55 common targets**  **between drug and disease** |
| --- | --- | --- |
| OPRK1 | MIR452 | GABRA2 |
| OPRM1 | MATR3 | MMP9 |
| ADORA2A | TBX1 | MMP2 |
| JAK1 | HLA-DRB1 | PGR |
| LRRK2 | COL11A1 | TYK2 |
| GABRB3 | GIPC1 | LCK |
| GABRG2 | LACTB | ACHE |
| GABRA5 | NOTCH2NLC | PTGS2 |
| GABRA2 | LRP12 | DRD2 |
| MAP2K1 | CRP | HTR2A |
| MET | IL1B | SLC6A3 |
| MMP9 | ADH1C | SLC6A4 |
| MMP1 | TNF | STAT3 |
| MMP2 | PTGS2 | EGFR |
| MMP8 | DGCR6 | CASP3 |
| OPRD1 | CXCL8 | SRC |
| HDAC6 | MEFV | MMP3 |
| HDAC1 | ADH5 | F3 |
| TTL | TLR2 | ELANE |
| POLA1 | IL6 | TGFBR1 |
| NR3C1 | MYOM2 | AKT1 |
| JAK2 | PABPN1 | PIK3CA |
| ALOX5 | FCGR1A | TNF |
| ROCK2 | CSF2 | IL6 |
| ROCK1 | CD4 | ALDH2 |
| PGR | CD8A | TGFBR2 |
| CYP19A1 | ELANE | BMP4 |
| CCNC | TANGO2 | ACVR1 |
| CDK8 | LGALS3 | P2RX7 |
| KIT | CD1A | CDK4 |
| CCR1 | TLR1 | GRIA1 |
| KDR | TP53 | SLC1A3 |
| PDE10A | BMP4 | CYP1A1 |
| PIM1 | CCR6 | KDM5B  (disconnected with other protein targets) |
| JAK3 | HLA-B | ERBB2 |
| TYK2 | SPON2 | CCND1 |
| SERPINA6 | CARD8 | PPARA |
| MDM2 | PIK3AP1 | CDC45 |
| PSEN2 | SPAG7 | CYP26A1 |
| PTPRF | NLRP3 | PLG |
| ERN1 | ZBTB7A | MAOA |
| RET | MVK | XIAP |
| LCK | ADH1B | MAOB |
| MAPK1 | SLC34A1 | IL2 |
| MKNK2 | DGCR8 | F2 |
| ALK | DGCR2 | PDGFRA |
| EPHB4 | ESS2 | ADH5 |
| IMPDH1 | GSTM1 | CREBBP |
| IMPDH2 | GPT | MMP7 |
| ACHE | CTNS | CASP1 |
| PTGS2 | SH2D1A | MPO |
| DRD2 | FGF8 | COMT |
| CHRNA4 | ALS2 | ASAH1  (disconnected with other protein targets) |
| DRD3 | XIAP | TLR7 |
| DRD1 | TNFRSF1A | BLK |
| HTR1A | NFKB2 |  |
| HTR7 | NFKB1 |  |
| HTR6 | F12 |  |
| HTR2B | MSX1 |  |
| PTPRCAP | SPEG |  |
| HTR2A | IFNG |  |
| ADRA1D | NKX2-6 |  |
| TH | CDH1 |  |
| DRD4 | CXCR4 |  |
| HTR5A | EDN1 |  |
| ADRA1A | GRIN1 |  |
| HTR1D | CD19 |  |
| ADRB1 | BCL2L2-PABPN1 |  |
| SLC6A3 | PTPN22 |  |
| DRD5 | TP63 |  |
| HTR2C | MIR21 |  |
| KCNH2 | IL4 |  |
| ADRA1B | PLG |  |
| TSPO | ATP1A3 |  |
| SLC6A4 | SERPING1 |  |
| HRH2 | ALB |  |
| ADRA2A | LAMA2 |  |
| ADRA2C | IRF6 |  |
| ADRA2B | COMT |  |
| HTR1B | TAF1 |  |
| PTGES | P4HA2 |  |
| CDK5R1 | SLC29A3 |  |
| DYRK1A | ALG12 |  |
| MAPK14 | GSTP1 |  |
| RBP4 | GRIN2B |  |
| TRPC6 | ALDH2 |  |
| TRPC3 | GSC2 |  |
| ALOX15 | CACNA1A |  |
| IRAK4 | NECTIN1 |  |
| ADRB2 | DOCK8 |  |
| STAT3 | DLG1 |  |
| MAPKAPK2 | GRHL3 |  |
| CDK1 | DGCR6L |  |
| PPARG | BCL2 |  |
| CCKBR | CYP1A1 |  |
| PPP5C | GSTM3 |  |
| CCNB3 | CR2 |  |
| GSK3B | TLR3 |  |
| NR3C2 | HLA-DQB1 |  |
| EGFR | HLA-A |  |
| MGLL | DGCR |  |
| ALOX12 | CALCA |  |
| AKR1B1 | PTEN |  |
| CASP3 | VEGFA |  |
| AURKA | IL10 |  |
| SIGMAR1 | MIR29C |  |
| SLC9A1 | SLC1A3 |  |
| PREP | ATP1A2 |  |
| HSD17B3 | KCNQ2 |  |
| RPS6KB1 | ASAH1 |  |
| FAP | ADNP |  |
| RORC | ARHGAP29 |  |
| CDK2 | DLX4 |  |
| AR | ISL1 |  |
| GABRA1 | PRKCD |  |
| EPHX2 | RAB11A |  |
| MIF | DYNC1H1 |  |
| HPGD | HTT |  |
| FLT3 | XPNPEP2 |  |
| MAPK3 | TSEN15 |  |
| CSF1R | SETD1B |  |
| PRKCG | TSEN34 |  |
| LIPG | TSEN54 |  |
| CCNE1 CDK2 | IL2 |  |
| HSD17B2 | KRT13 |  |
| CCNE1 | CLPB |  |
| DBF4 | GFI1 |  |
| ADAMTS5 | MYD88 |  |
| PDGFRA PDGFRB | SNCA |  |
| SRC | GSTT1 |  |
| MMP3 | CD70 |  |
| CYP17A1 | TAOK1 |  |
| FGFR1 | SCN2A |  |
| EIF2AK2 | HTR2A |  |
| F3 | GABRA2 |  |
| RPS6KA3 | SF3B2 |  |
| PDPK1 | TAS2R16 |  |
| PTGS1 | FAS |  |
| CHRM1 | MGMT |  |
| PRSS1 | HLA-DPB1 |  |
| NCOA2 | MIR132 |  |
| NCOA1 | CFHR5 |  |
| CHRM3 | HLA-DQA1 |  |
| MMP13 | ERBB4 |  |
| CLK4 | CAMK2A |  |
| CLK1 | PPP3CA |  |
| CLK2 | CAMK2B |  |
| DYRK1B | CSNK2B |  |
| CNR1 | SLC2A3 |  |
| CA12 | YY1 |  |
| PARP2 | ADCY6 |  |
| ELANE | TCF4 |  |
| SIRT2 | CACNA1I |  |
| PDE4A | CLTC |  |
| TGFBR1 | EEF1A2 |  |
| VCAM1 | SYNGAP1 |  |
| TAAR1 | DLL1 |  |
| FAAH | KDM5B |  |
| AKT1 | CACNG2 |  |
| CLK3 | CDH15 |  |
| PLK1 | CUX1 |  |
| DYRK2 | KIF1A |  |
| PLK2 | SEPSECS |  |
| FLT1 | SET |  |
| AURKB | UBB |  |
| ABL1 | ASPA |  |
| CTSK | BRSK2 |  |
| PIK3CD | CNTNAP1 |  |
| PIK3CB | KIRREL3 |  |
| PIK3CG | NGLY1 |  |
| CAPN1 | CIC |  |
| PIK3CA | EPB41L1 |  |
| TRPV1 | ITSN1 |  |
| PDE3A | KCNQ5 |  |
| RXRB | MYT1L |  |
| FNTA FNTB | EXOSC9 |  |
| SHBG | FLCN |  |
| TNF | NBEA |  |
| HSD11B1 | SCUBE3 |  |
| NOS2 | TRPM3 |  |
| PRKCH | TSEN2 |  |
| CDC25A | ZMYND11 |  |
| NPC1L1 | CHAMP1 |  |
| BACE1 | HIVEP2 |  |
| BCHE | PRICKLE2 |  |
| SRD5A2 | DEAF1 |  |
| RORA | RAI1 |  |
| ITGAL | ASH1L |  |
| ADORA3 | MED12L |  |
| ESR1 | MBD5 |  |
| ESR2 | ZNF699 |  |
| CES2 | MTRFR |  |
| HSD11B2 | CDKN2A |  |
| POLB | MS4A1 |  |
| AKR1B10 | CD81 |  |
| IDO1 | TNFRSF13B |  |
| PTPN11 | ICOS |  |
| FDFT1 | TNFRSF13C |  |
| PTPN1 | TNFSF12 |  |
| PTPN2 | IRF2BP2 |  |
| CES1 | MID1 |  |
| MAPK8 | SCN8A |  |
| MAPK9 | DMPK |  |
| SRD5A1 | PI4KA |  |
| NR1I2 | ECM1 |  |
| G6PD | GNAI3 |  |
| FABP1 | PLCB4 |  |
| SLC22A6 | TYK2 |  |
| CYP51A1 | NPM1 |  |
| SF3B3 | IKZF1 |  |
| PGK1 | HP |  |
| IL6 | ATM |  |
| GLUL | CD44 |  |
| SYK | PIK3CA |  |
| MTOR | CP |  |
| CYP2D6 | H2AC18 |  |
| SAE1 | RIPPLY3 |  |
| CDC42 | DRD2 |  |
| RAC1 | TNFRSF10B |  |
| CDK2 | PRKD1 |  |
| CDC25B | GATM |  |
| HSD17B1 | EHHADH |  |
| MKNK1 | ING1 |  |
| PFKFB3 | LGI3 |  |
| HTR3A | ING3 |  |
| PNMT | NDUFAF6 |  |
| WEE1 | RILPL1 |  |
| ABCG2 | MIR98 |  |
| TERT | EA3 |  |
| CHRNA7 | EA7 |  |
| MCL1 | EA8 |  |
| DAGLA | IL7 |  |
| SCN4A | CXCL9 |  |
| ALDH2 | IFIH1 |  |
| PRKAB1 | BDNF |  |
| TGFBR2 | TLR5 |  |
| BMPR1A | EDA |  |
| ACVRL1 | ADGRF5 |  |
| BMP4 | MIR146A |  |
| BMPR2 | LEP |  |
| BMPR1B | HIRA |  |
| ACVR1 | TCIRG1 |  |
| TBXAS1 | SRP54 |  |
| P2RX7 | MIR185 |  |
| CDK4 | CTLA4 |  |
| TYMS | HIF1A |  |
| ALDH3A1 | CTNS-AS1 |  |
| CD38 | RAG2 |  |
| TTK | FASLG |  |
| PRF1 | CCND1 |  |
| CASP7 | MAOA |  |
| ZAP70 | MAOB |  |
| TRPM8 | COL5A1 |  |
| PLAA | COL5A2 |  |
| CCNC CDK8 | NFIX |  |
| PAK4 | SOD1 |  |
| AGPAT2 | IL1A |  |
| BRAF | ADGRG1 |  |
| CETP | UFD1 |  |
| PRKCE | CD40LG |  |
| HSP90AA1 | PRODH |  |
| F7 | ICAM1 |  |
| PLK4 | LOXL3 |  |
| CCNE1 | STAT1 |  |
| CDK3 | ENO2 |  |
| RAF1 | MBL2 |  |
| OGT | MED15 |  |
| GRIA1 | CD27 |  |
| TBXA2R | ATXN2 |  |
| NR1D1 | MRPL40 |  |
| CPT1A | SPECC1L |  |
| HDAC10 | CXCL12 |  |
| PARP10 | ACE2 |  |
| CSNK1D | ACP1 |  |
| PLK3 | AASS |  |
| CHEK2 | CTTN |  |
| SLC1A3 | SDHB |  |
| CA2 | BMP2 |  |
| PRKDC | HOXA3 |  |
| HCK | MYOT |  |
| PI4KB | EBAG9 |  |
| LIMK1 | HLA-C |  |
| PTK6 | ATP2B3 |  |
| LIMK2 | COL2A1 |  |
| NEK2 | CRKL |  |
| SAE1 UBA2 | HOXA1 |  |
| GRM5 | COL11A2 |  |
| CYP1A1 | DGCR5 |  |
| NQO1 | FGF10 |  |
| NQO2 | SPP1 |  |
| CYP1B1 | SOX3 |  |
| NTRK1 | SYP |  |
| MARK1 | RIGI |  |
| KDM5B | IFNA1 |  |
| ERBB2 | RASSF1 |  |
| GRM1 | FADD |  |
| DHFR | EGFR |  |
| NAAA | CSF3 |  |
| PPIA | PDGFRA |  |
| LYN | GRIA1 |  |
| TEK | TUBA1A |  |
| SCD | SLC6A1 |  |
| CYP11B2 | FTL |  |
| CCND1 | REV3L |  |
| GCK | EDA2R |  |
| MAPK10 | ERLIN2 |  |
| MCHR1 | PLXND1 |  |
| HCRTR2 | SRPX2 |  |
| CFD | VPS53 |  |
| IKBKB | ARF3 |  |
| FPR2 | TTC7A |  |
| RPS6KA2 | AP4E1 |  |
| NAMPT | SPATA22 |  |
| MST1R | TEX49 |  |
| FLT4 | SPECC1L-ADORA2A |  |
| ITK | DM1-AS |  |
| PTGER2 | LOC107075317 |  |
| PDGFRB | LOC109461479 |  |
| FABP4 | CELIAC2 |  |
| PPARA | LOC109461477 |  |
| FABP3 | MBS1 |  |
| FABP5 | CELIAC10 |  |
| PPARD | CELIAC11 |  |
| FABP2 | CELIAC12 |  |
| FFAR1 | CELIAC13 |  |
| VDR | CELIAC5 |  |
| NR1H4 | CELIAC6 |  |
| PHF8 | CELIAC7 |  |
| UGT2B7 | CELIAC8 |  |
| GABBR1 | CELIAC9 |  |
| KDM2A | PTLS |  |
| KDM5C | CDK4 |  |
| GPBAR1 | ACE |  |
| FNTA | IL5 |  |
| HAO1 | SNAI1 |  |
| PTGFR | COL1A1 |  |
| GSTK1 | SLC25A1 |  |
| LTA4H | TBX5 |  |
| CA1 | CDC45 |  |
| NR0B2 | COL9A2 |  |
| CDC45 | LZTR1 |  |
| PTPRC | MATN3 |  |
| CYP26A1 | RANBP1 |  |
| RXRA | COL9A1 |  |
| RARG | COL9A3 |  |
| RARB | FMOD |  |
| RARA | CLTCL1 |  |
| CYP26B1 | COL27A1 |  |
| RXRG | PLEKHA7 |  |
| FFAR4 | COL20A1 |  |
| CACNA2D1 | ZNF74 |  |
| SLC16A1 | GNB1L |  |
| PTGER4 | SMAP2 |  |
| GLRA1 | HIC2 |  |
| ABCC1 | PCMTD1 |  |
| ABCB1 | SEPTIN5 |  |
| PLA2G4A | EEIG1 |  |
| PLG | MIR196A1 |  |
| RORB | MIR9-1 |  |
| PTGDR2 | SLAMF1 |  |
| HMGCR | DES |  |
| GCG | POF1B |  |
| PDE4B | MUC1 |  |
| DHCR7 | IFNB1 |  |
| MAOA | SELE |  |
| CHRNA4 | NAT2 |  |
| DPP4 | NKX2-5 |  |
| CHRM4 | TLR7 |  |
| CHRNA3 | STAT3 |  |
| RBBP9 | MIR140 |  |
| ADRB3 | DEFB4A |  |
| MTNR1B | EPHX1 |  |
| KCNN1 | DVL1 |  |
| KCNN3 | VAPB |  |
| KCNN2 | FIG4 |  |
| SLC6A2 | FUS |  |
| SLC18A2 | PRPH |  |
| KDM1A | SETX |  |
| MTNR1A | CAMK2N2 |  |
| SLC47A1 | IGF1 |  |
| PRKCQ | CD36 |  |
| CHRNB4 | VCAN |  |
| DPP7 | PITX2 |  |
| PARP1 | TF |  |
| CHEK1 | ICOSLG |  |
| CHRNB1 | EDNRA |  |
| JUN | INS |  |
| DPP8 | TLR4 |  |
| HRH1 | IL18 |  |
| XIAP | HOXB1 |  |
| HRH3 | FCGR3B |  |
| DPP9 | TAC1 |  |
| PRMT6 | CYP2E1 |  |
| PRMT8 | NOD2 |  |
| PRMT1 | TCOF1 |  |
| HCRTR1 | TMPRSS2 |  |
| CA7 | ERVW-1 |  |
| NR4A1 | NRP1 |  |
| MAOB | CHAF1A |  |
| HRH4 | USP22 |  |
| KIF11 | CD274 |  |
| AXL | MTHFR |  |
| TYRO3 | G6PC3 |  |
| MERTK | HOXA2 |  |
| CDK9 | DMAP1 |  |
| CYP1A2 | YPEL1 |  |
| BIRC2 | CD46 |  |
| PDE1A | MMP9 |  |
| HTR1E | MMP11 |  |
| HTR1F | TGFB1 |  |
| CHRM5 | HSPG2 |  |
| CHRM2 | SLC6A3 |  |
| BCL2L1 | IFNAR1 |  |
| IL2 | CSF3R |  |
| PPM1B | DMD |  |
| PPP1CC | PDPN |  |
| PPP2CA | CYP3A4 |  |
| PPP2R5A | MIR143 |  |
| GBA | MIR145 |  |
| F2 | MIR149 |  |
| GLRA2 | MIR204 |  |
| ATP1A1 | MIR27A |  |
| BDKRB2 | MIR30E |  |
| PDGFRA | MIR106B |  |
| PDE2A | MIR10B |  |
| PDE3B | MIR141 |  |
| BAZ2B | MIR142 |  |
| BAZ2A | MIR150 |  |
| ADORA1 | MIR99A |  |
| GRM4 | MIR125A |  |
| CYP11B1 | MIR139 |  |
| TLR9 | MIR155 |  |
| MPI | MIR221 |  |
| EP300 | MIR148A |  |
| BTK | MIR17 |  |
| SLC10A2 | MIR193A |  |
| BRD9 | MIR195 |  |
| SLC5A1 | MIR199A1 |  |
| PDE4D | MIR205 |  |
| PTGIR | MIR30A |  |
| PTGER3 | MIR31 |  |
| PTPN6 | MIR18A |  |
| KMT5A | MIR423 |  |
| PTGDR | MIR486-1 |  |
| NR1H3 | MIR15A |  |
| ROS1 | FAM3D-AS1 |  |
| SLC5A2 | EGF |  |
| UPP1 | AKR1A1 |  |
| ADK | DSP |  |
| PDE5A | PGR |  |
| ADH5 | TNC |  |
| SLC5A4 | CASP1 |  |
| SLC28A2 | CCL2 |  |
| SLC29A1 | SERPINA3 |  |
| HK2 | ADH1A |  |
| HK1 | CNTNAP2 |  |
| HSPA5 | SMARCA4 |  |
| HSPA8 | NFE2L2 |  |
| SLC5A11 | MIR330 |  |
| MAN1B1 | JUP |  |
| MAPK11 | ZEB2 |  |
| EPHA5 | LAMC3 |  |
| GAK | PPL |  |
| CDC7 | GPN1 |  |
| CA14 | LHPP |  |
| CA4 | MIR1269B |  |
| CA13 | NPY |  |
| TOP2A | ACHE |  |
| PTAFR | HSPA4 |  |
| BRD4 | BLK |  |
| CREBBP | FCGR3A |  |
| CDK5R1 | SLC11A1 |  |
| ABCC9 | MPO |  |
| BRD2 | GZMB |  |
| FKBP1A | FYN |  |
| CA5B | FZD4 |  |
| CA5A | LRP5 |  |
| PYGL | DROSHA |  |
| KCNA5 | TBX3 |  |
| F2RL3 | TSSK2 |  |
| ADAM17 | GTF3A |  |
| MMP14 | TBX10 |  |
| MMP7 | DGCR11 |  |
| CA3 | DVL1P1 |  |
| CA6 | ENSG00000278817 |  |
| CA9 | LOC108449888 |  |
| MME | CYP26A1 |  |
| CASP1 | PLAUR |  |
| MPO | SELP |  |
| ICMT | CASP3 |  |
| HSP90AB1 | GATA4 |  |
| HMOX1 | STAT2 |  |
| COMT | GAL |  |
| ASAH1 | PPARA |  |
| APP | TWIST1 |  |
| NUDT1 | MSN |  |
| PER2 | CPA6 |  |
| MAP3K8 | TGFBR2 |  |
| PSMB5 | XRCC3 |  |
| AVPR1A | SLURP1 |  |
| KCNK3 | ACVR1 |  |
| KCNK9 | CDKN1A |  |
| NOS1 | EYA1 |  |
| PDE11A | FCGR2A |  |
| TLR7 | CREBBP |  |
| AHR | NAGLU |  |
| TNKS | TAFAZZIN |  |
| TACR3 | USB1 |  |
| NPY5R | CSF1 |  |
| TRPA1 | HCRT |  |
| DUT | EMP1 |  |
| YES1 | SERPINB13 |  |
| BLK | KRT5 |  |
| BAD | CYP2C9 |  |
| MAP3K20 | ALDH1A2 |  |
| AURKC | KAT6A |  |
| FGFR4 | TGFBR1 |  |
| STK10 | ERBB2 |  |
| ABL2 | LCK |  |
| SLK | MAPT |  |
| FRK | TAOK3 |  |
| FGR | GGT1 |  |
| PIM2 | NLRC4 |  |
| EPHA6 | MMP2 |  |
| RIPK2 | AQP1 |  |
| TNNI3K | CTSD |  |
| DDR1 | NRG1 |  |
| EIF2AK4 | P2RX7 |  |
| CIT | CS |  |
| STK35 | EPX |  |
| MAP3K7 | APEH |  |
| MAP3K2 | NKX2-3 |  |
| MAP3K3 | SERPINB1 |  |
| MAP4K4 | KRT24 |  |
| MAP3K19 | RNU4ATAC |  |
| DBF4 | TTR |  |
| CTSL | CTNNB1 |  |
| IDH1 | CYP2C8 |  |
| AGTR1 | PTCH1 |  |
| ALPL | MASP2 |  |
| TOP1 | FCN2 |  |
| CASP6 | NPPB |  |
| CASP8 | NCAM1 |  |
| IGFBP3 | KNG1 |  |
| ADORA2B | IL1R1 |  |
|  | AKT1 |  |
|  | MMP3 |  |
|  | F2 |  |
|  | GAA |  |
|  | MYOD1 |  |
|  | F3 |  |
|  | MMP7 |  |
|  | EPCAM |  |
|  | SLC6A4 |  |
|  | SERPINC1 |  |
|  | UGT1A6 |  |
|  | HOXA |  |
|  | IL1RN |  |
|  | HSPD1 |  |
|  | TGM2 |  |
|  | AP3B1 |  |
|  | TNFRSF10A |  |
|  | CXCR3 |  |
|  | FMR1 |  |
|  | HAX1 |  |
|  | IL13 |  |
|  | IL17A |  |
|  | LAMP1 |  |
|  | SBDS |  |
|  | OGG1 |  |
|  | CD79A |  |
|  | XRCC2 |  |
|  | FBXW11 |  |
|  | SRC |  |
|  | RHOA |  |
|  | CHD7 |  |
|  | NGF |  |
|  | GHRL |  |
|  | YAP1 |  |
|  | RPL4 |  |
|  | UGT1A |  |
|  | CREB1 |  |
|  | CYP2B6 |  |

# Table S1C PPI network

| **Target A** | **Target B** |
| --- | --- |
| ACVR1 | BMP4 |
| ADH5 | CYP26A1 |
| ADH5 | ALDH2 |
| AKT1 | IL2 |
| AKT1 | CREBBP |
| AKT1 | PIK3CA |
| AKT1 | STAT3 |
| AKT1 | CASP3 |
| AKT1 | LCK |
| AKT1 | XIAP |
| AKT1 | SRC |
| ALDH2 | MAOB |
| ALDH2 | COMT |
| ALDH2 | MAOA |
| BLK | SRC |
| BLK | LCK |
| BLK | STAT3 |
| BLK | EGFR |
| BMP4 | XIAP |
| CASP1 | CASP3 |
| CASP3 | XIAP |
| CCND1 | CREBBP |
| CCND1 | SRC |
| CCND1 | STAT3 |
| CCND1 | CDK4 |
| CDK4 | SRC |
| COMT | MAOA |
| COMT | DRD2 |
| COMT | CYP1A1 |
| COMT | MAOB |
| CREBBP | SRC |
| CREBBP | CYP1A1 |
| CREBBP | TNF |
| CREBBP | PPARA |
| CREBBP | STAT3 |
| CYP1A1 | CYP26A1 |
| CYP1A1 | PPARA |
| DRD2 | SLC6A3 |
| EGFR | PDGFRA |
| EGFR | PIK3CA |
| EGFR | STAT3 |
| EGFR | ERBB2 |
| EGFR | LCK |
| EGFR | SRC |
| ELANE | MPO |
| ELANE | MMP7 |
| ERBB2 | PIK3CA |
| ERBB2 | STAT3 |
| ERBB2 | PGR |
| ERBB2 | IL6 |
| ERBB2 | SRC |
| F2 | PPARA |
| F2 | SRC |
| F2 | F3 |
| F3 | PLG |
| HTR2A | SLC6A4 |
| IL2 | IL6 |
| IL2 | PIK3CA |
| IL2 | TNF |
| IL2 | STAT3 |
| IL2 | LCK |
| IL6 | MMP2 |
| IL6 | STAT3 |
| IL6 | MMP3 |
| IL6 | PTGS2 |
| IL6 | MMP9 |
| IL6 | TYK2 |
| IL6 | TNF |
| LCK | PIK3CA |
| LCK | STAT3 |
| LCK | SRC |
| LCK | TYK2 |
| MMP2 | MMP9 |
| MMP2 | SRC |
| MMP2 | STAT3 |
| MMP3 | STAT3 |
| MMP3 | MMP9 |
| MMP3 | PLG |
| MMP9 | STAT3 |
| MMP9 | PLG |
| MMP9 | SRC |
| PDGFRA | STAT3 |
| PDGFRA | PIK3CA |
| PGR | PIK3CA |
| PGR | STAT3 |
| PGR | SRC |
| PIK3CA | TYK2 |
| PIK3CA | STAT3 |
| PIK3CA | SRC |
| PPARA | TNF |
| PTGS2 | STAT3 |
| SRC | STAT3 |
| STAT3 | TNF |
| STAT3 | TYK2 |
| TGFBR1 | TGFBR2 |
| TGFBR1 | XIAP |
| TGFBR2 | XIAP |

# Table S1D GO enrichment analysis

| **Category** | **Term** | **Genes** | **Count** | **%** | **P-Value** | **Benjamini** |
| --- | --- | --- | --- | --- | --- | --- |
| BP | response to xenobiotic stimulus | 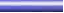 | 16 | 30.2 | 9.80E-17 | 1.40E-13 |
| BP | positive regulation of smooth muscle cell proliferation | 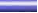 | 9 | 17 | 2.70E-12 | 1.90E-09 |
| BP | negative regulation of apoptotic process | 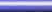 | 13 | 24.5 | 9.70E-09 | 3.70E-06 |
| BP | response to hypoxia | 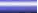 | 9 | 17 | 1.00E-08 | 3.70E-06 |
| BP | positive regulation of MAP kinase activity | 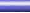 | 7 | 13.2 | 5.70E-08 | 1.70E-05 |
| BP | peptidyl-tyrosine phosphorylation | 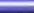 | 8 | 15.1 | 8.60E-08 | 2.10E-05 |
| BP | response to lipopolysaccharide | 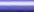 | 8 | 15.1 | 1.00E-07 | 2.10E-05 |
| BP | extracellular matrix disassembly | 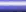 | 6 | 11.3 | 1.40E-07 | 2.50E-05 |
| BP | positive regulation of cell proliferation | 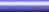 | 12 | 22.6 | 1.60E-07 | 2.50E-05 |
| BP | positive regulation of cell migration | 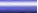 | 9 | 17 | 4.60E-07 | 6.60E-05 |
| BP | positive regulation of interleukin-8 production | 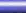 | 6 | 11.3 | 8.40E-07 | 1.10E-04 |
| BP | positive regulation of gene expression | 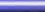 | 11 | 20.8 | 9.10E-07 | 1.10E-04 |
| BP | positive regulation of protein phosphorylation | 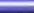 | 8 | 15.1 | 1.50E-06 | 1.60E-04 |
| BP | proteolysis | 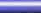 | 10 | 18.9 | 1.50E-06 | 1.60E-04 |
| BP | dopamine catabolic process | 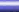 | 4 | 7.5 | 2.20E-06 | 2.10E-04 |
| BP | positive regulation of apoptotic process | 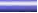 | 9 | 17 | 2.40E-06 | 2.20E-04 |
| BP | aging | 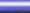 | 7 | 13.2 | 3.20E-06 | 2.70E-04 |
| BP | cellular response to reactive oxygen species | 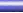 | 5 | 9.4 | 4.40E-06 | 3.40E-04 |
| BP | positive regulation of peptidyl-serine phosphorylation | 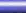 | 6 | 11.3 | 4.50E-06 | 3.40E-04 |
| BP | positive regulation of cell growth | 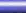 | 6 | 11.3 | 4.70E-06 | 3.40E-04 |
| BP | wound healing | 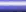 | 6 | 11.3 | 5.00E-06 | 3.40E-04 |
| BP | protein phosphorylation | 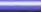 | 10 | 18.9 | 5.50E-06 | 3.60E-04 |
| BP | response to ethanol | 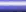 | 6 | 11.3 | 9.90E-06 | 6.30E-04 |
| BP | response to mechanical stimulus | 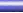 | 5 | 9.4 | 1.50E-05 | 9.30E-04 |
| BP | positive regulation of transcription, DNA-templated | 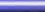 | 11 | 20.8 | 1.90E-05 | 1.10E-03 |
| BP | heart development | 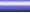 | 7 | 13.2 | 2.00E-05 | 1.10E-03 |
| BP | response to UV-A | 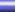 | 3 | 5.7 | 2.10E-05 | 1.10E-03 |
| BP | positive regulation of protein kinase B signaling | 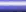 | 6 | 11.3 | 2.50E-05 | 1.30E-03 |
| BP | positive regulation of interleukin-1 beta production | 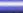 | 5 | 9.4 | 2.60E-05 | 1.30E-03 |
| BP | transmembrane receptor protein tyrosine kinase signaling pathway | 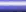 | 6 | 11.3 | 2.80E-05 | 1.40E-03 |
| BP | positive regulation of ERK1 and ERK2 cascade | 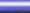 | 7 | 13.2 | 3.10E-05 | 1.40E-03 |
| BP | negative regulation of cysteine-type endopeptidase activity involved in apoptotic process | 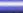 | 5 | 9.4 | 3.10E-05 | 1.40E-03 |
| BP | platelet activation | 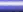 | 5 | 9.4 | 3.30E-05 | 1.50E-03 |
| BP | protein catabolic process | 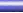 | 5 | 9.4 | 4.60E-05 | 2.00E-03 |
| BP | positive regulation of transcription from RNA polymerase II promoter | 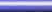 | 13 | 24.5 | 6.40E-05 | 2.70E-03 |
| BP | positive regulation of epithelial to mesenchymal transition involved in endocardial cushion formation | 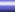 | 3 | 5.7 | 7.00E-05 | 2.80E-03 |
| BP | embryonic cranial skeleton morphogenesis | 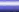 | 4 | 7.5 | 7.80E-05 | 3.10E-03 |
| BP | regulation of cell proliferation | 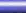 | 6 | 11.3 | 9.40E-05 | 3.60E-03 |
| BP | response to estradiol | 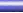 | 5 | 9.4 | 1.00E-04 | 3.90E-03 |
| BP | extracellular matrix organization | 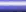 | 6 | 11.3 | 1.30E-04 | 4.60E-03 |
| BP | response to nicotine | 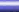 | 4 | 7.5 | 1.30E-04 | 4.60E-03 |
| BP | negative regulation of extrinsic apoptotic signaling pathway | 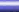 | 4 | 7.5 | 1.30E-04 | 4.60E-03 |
| BP | transforming growth factor beta receptor signaling pathway | 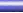 | 5 | 9.4 | 1.40E-04 | 4.70E-03 |
| BP | regulation of multicellular organismal process | 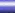 | 3 | 5.7 | 1.50E-04 | 4.70E-03 |
| BP | positive regulation of nitrogen compound metabolic process | 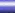 | 3 | 5.7 | 1.50E-04 | 4.70E-03 |
| BP | positive regulation of interleukin-6 production | 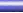 | 5 | 9.4 | 1.70E-04 | 5.10E-03 |
| BP | T cell costimulation | 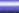 | 4 | 7.5 | 1.70E-04 | 5.10E-03 |
| BP | positive regulation of cyclin-dependent protein serine/threonine kinase activity | 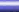 | 4 | 7.5 | 1.70E-04 | 5.10E-03 |
| BP | collagen catabolic process | 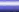 | 4 | 7.5 | 1.80E-04 | 5.40E-03 |
| BP | intracellular signal transduction | 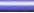 | 8 | 15.1 | 1.90E-04 | 5.50E-03 |
| BP | neurotransmitter catabolic process | 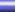 | 3 | 5.7 | 2.00E-04 | 5.60E-03 |
| BP | positive regulation of JAK-STAT cascade | 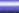 | 4 | 7.5 | 2.10E-04 | 5.60E-03 |
| BP | neurotransmitter transport | 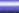 | 4 | 7.5 | 2.10E-04 | 5.60E-03 |
| BP | embryo implantation | 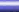 | 4 | 7.5 | 2.10E-04 | 5.60E-03 |
| BP | phosphatidylinositol 3-kinase signaling | 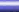 | 4 | 7.5 | 2.40E-04 | 6.30E-03 |
| BP | response to activity | 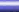 | 4 | 7.5 | 2.70E-04 | 7.10E-03 |
| BP | positive regulation of pathway-restricted SMAD protein phosphorylation | 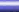 | 4 | 7.5 | 3.30E-04 | 8.30E-03 |
| BP | positive regulation of G1/S transition of mitotic cell cycle | 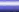 | 4 | 7.5 | 3.50E-04 | 8.30E-03 |
| BP | protein kinase B signaling | 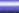 | 4 | 7.5 | 3.50E-04 | 8.30E-03 |
| BP | epidermal growth factor receptor signaling pathway | 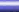 | 4 | 7.5 | 3.50E-04 | 8.30E-03 |
| BP | positive regulation of vascular smooth muscle cell proliferation | 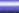 | 4 | 7.5 | 3.70E-04 | 8.30E-03 |
| BP | release of sequestered calcium ion into cytosol | 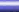 | 4 | 7.5 | 3.70E-04 | 8.30E-03 |
| BP | positive regulation of pri-miRNA transcription from RNA polymerase II promoter | 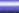 | 4 | 7.5 | 3.70E-04 | 8.30E-03 |
| BP | positive regulation of epithelial to mesenchymal transition | 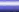 | 4 | 7.5 | 3.70E-04 | 8.30E-03 |
| BP | cellular response to UV-A | 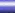 | 3 | 5.7 | 3.80E-04 | 8.50E-03 |
| BP | signal transduction | 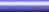 | 12 | 22.6 | 4.30E-04 | 9.40E-03 |
| BP | positive regulation of production of miRNAs involved in gene silencing by miRNA | 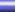 | 3 | 5.7 | 4.60E-04 | 9.90E-03 |
| BP | response to glucocorticoid | 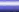 | 4 | 7.5 | 4.80E-04 | 1.00E-02 |
| BP | cellular response to growth factor stimulus | 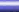 | 4 | 7.5 | 5.10E-04 | 1.10E-02 |
| BP | cellular response to amino acid stimulus | 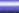 | 4 | 7.5 | 5.30E-04 | 1.10E-02 |
| BP | interleukin-6-mediated signaling pathway | 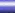 | 3 | 5.7 | 6.30E-04 | 1.30E-02 |
| BP | response to beta-amyloid | 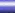 | 3 | 5.7 | 6.30E-04 | 1.30E-02 |
| BP | cellular response to fluid shear stress | 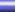 | 3 | 5.7 | 6.30E-04 | 1.30E-02 |
| BP | pathway-restricted SMAD protein phosphorylation | 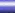 | 3 | 5.7 | 7.30E-04 | 1.40E-02 |
| BP | cytokine-mediated signaling pathway | 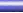 | 5 | 9.4 | 8.10E-04 | 1.60E-02 |
| BP | inflammatory response | 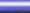 | 7 | 13.2 | 8.20E-04 | 1.60E-02 |
| BP | response to iron ion | 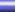 | 3 | 5.7 | 8.30E-04 | 1.60E-02 |
| BP | peptidyl-threonine phosphorylation | 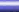 | 4 | 7.5 | 8.80E-04 | 1.60E-02 |
| BP | positive regulation of MAPK cascade | 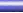 | 5 | 9.4 | 9.40E-04 | 1.70E-02 |
| BP | positive regulation of endothelial cell proliferation | 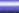 | 4 | 7.5 | 1.10E-03 | 1.90E-02 |
| BP | positive regulation of interferon-gamma production | 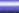 | 4 | 7.5 | 1.30E-03 | 2.20E-02 |
| BP | protein autophosphorylation | 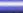 | 5 | 9.4 | 1.40E-03 | 2.40E-02 |
| BP | negative regulation of gene expression | 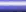 | 6 | 11.3 | 1.40E-03 | 2.50E-02 |
| BP | temperature homeostasis | 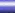 | 3 | 5.7 | 1.40E-03 | 2.50E-02 |
| BP | activin receptor signaling pathway | 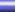 | 3 | 5.7 | 1.40E-03 | 2.50E-02 |
| BP | cellular response to lipopolysaccharide | 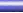 | 5 | 9.4 | 1.50E-03 | 2.60E-02 |
| BP | positive regulation of phosphatidylinositol 3-kinase signaling | 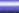 | 4 | 7.5 | 1.50E-03 | 2.60E-02 |
| BP | peptidyl-tyrosine autophosphorylation | 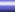 | 3 | 5.7 | 1.60E-03 | 2.60E-02 |
| BP | excitatory postsynaptic potential | 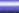 | 4 | 7.5 | 1.80E-03 | 2.90E-02 |
| BP | positive regulation of peptidyl-tyrosine phosphorylation | 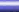 | 4 | 7.5 | 1.90E-03 | 3.00E-02 |
| BP | response to food | 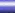 | 3 | 5.7 | 2.00E-03 | 3.20E-02 |
| BP | regulation of multicellular organism growth | 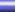 | 3 | 5.7 | 2.20E-03 | 3.40E-02 |
| BP | positive regulation of interleukin-17 production | 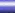 | 3 | 5.7 | 2.20E-03 | 3.40E-02 |
| BP | cell differentiation | 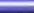 | 8 | 15.1 | 2.30E-03 | 3.50E-02 |
| BP | negative regulation of intrinsic apoptotic signaling pathway | 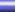 | 3 | 5.7 | 2.60E-03 | 3.90E-02 |
| BP | leukocyte migration | 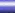 | 3 | 5.7 | 2.60E-03 | 3.90E-02 |
| BP | response to electrical stimulus | 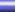 | 3 | 5.7 | 2.70E-03 | 4.10E-02 |
| BP | positive regulation of cytokine production involved in inflammatory response | 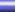 | 3 | 5.7 | 2.90E-03 | 4.30E-02 |
| BP | positive regulation of inflammatory response | 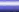 | 4 | 7.5 | 3.10E-03 | 4.50E-02 |
| BP | response to antibiotic | 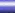 | 3 | 5.7 | 3.50E-03 | 5.10E-02 |
| BP | liver regeneration |  | 3 | 5.7 | 3.50E-03 | 5.10E-02 |
| BP | positive regulation of protein localization to nucleus |  | 3 | 5.7 | 3.80E-03 | 5.30E-02 |
| BP | cellular response to cadmium ion |  | 3 | 5.7 | 4.40E-03 | 6.20E-02 |
| BP | ventricular septum morphogenesis |  | 3 | 5.7 | 4.40E-03 | 6.20E-02 |
| BP | response to gold nanoparticle |  | 2 | 3.8 | 5.40E-03 | 7.30E-02 |
| BP | negative regulation of synaptic transmission, dopaminergic |  | 2 | 3.8 | 5.40E-03 | 7.30E-02 |
| BP | germ cell development |  | 3 | 5.7 | 5.70E-03 | 7.70E-02 |
| BP | positive regulation of bone mineralization |  | 3 | 5.7 | 6.00E-03 | 8.00E-02 |
| BP | cellular response to epidermal growth factor stimulus |  | 3 | 5.7 | 6.20E-03 | 8.30E-02 |
| BP | positive regulation of chemokine production |  | 3 | 5.7 | 6.50E-03 | 8.60E-02 |
| BP | positive regulation of nitric oxide biosynthetic process |  | 3 | 5.7 | 6.80E-03 | 8.90E-02 |
| BP | negative regulation of cell proliferation |  | 6 | 11.3 | 7.40E-03 | 9.60E-02 |
| BP | negative regulation of primary miRNA processing |  | 2 | 3.8 | 8.00E-03 | 1.00E-01 |
| BP | ephrin receptor signaling pathway |  | 3 | 5.7 | 8.30E-03 | 1.10E-01 |
| BP | positive regulation of protein localization to plasma membrane |  | 3 | 5.7 | 9.60E-03 | 1.20E-01 |
| BP | electron transport chain |  | 3 | 5.7 | 9.60E-03 | 1.20E-01 |
| BP | positive regulation of fibroblast proliferation |  | 3 | 5.7 | 9.60E-03 | 1.20E-01 |
| BP | neutrophil mediated killing of gram-negative bacterium |  | 2 | 3.8 | 1.10E-02 | 1.30E-01 |
| BP | response to estrogen |  | 3 | 5.7 | 1.10E-02 | 1.30E-01 |
| BP | cell surface receptor signaling pathway |  | 5 | 9.4 | 1.10E-02 | 1.30E-01 |
| BP | glucose metabolic process |  | 3 | 5.7 | 1.20E-02 | 1.50E-01 |
| BP | I-kappaB kinase/NF-kappaB signaling |  | 3 | 5.7 | 1.20E-02 | 1.50E-01 |
| BP | peptidyl-serine phosphorylation |  | 4 | 7.5 | 1.30E-02 | 1.50E-01 |
| BP | positive regulation of fever generation |  | 2 | 3.8 | 1.30E-02 | 1.50E-01 |
| BP | prostaglandin secretion |  | 2 | 3.8 | 1.30E-02 | 1.50E-01 |
| BP | positive regulation of protein transport |  | 2 | 3.8 | 1.30E-02 | 1.50E-01 |
| BP | response to fructose |  | 2 | 3.8 | 1.30E-02 | 1.50E-01 |
| BP | endocardial cushion fusion |  | 2 | 3.8 | 1.30E-02 | 1.50E-01 |
| BP | palate development |  | 3 | 5.7 | 1.40E-02 | 1.50E-01 |
| BP | learning or memory |  | 3 | 5.7 | 1.40E-02 | 1.50E-01 |
| BP | cell proliferation |  | 4 | 7.5 | 1.40E-02 | 1.50E-01 |
| BP | positive regulation of tyrosine phosphorylation of STAT protein |  | 3 | 5.7 | 1.40E-02 | 1.60E-01 |
| BP | response to wounding |  | 3 | 5.7 | 1.40E-02 | 1.60E-01 |
| BP | positive regulation of NIK/NF-kappaB signaling |  | 3 | 5.7 | 1.40E-02 | 1.60E-01 |
| BP | positive regulation of kinase activity |  | 3 | 5.7 | 1.60E-02 | 1.70E-01 |
| BP | dopamine uptake involved in synaptic transmission |  | 2 | 3.8 | 1.60E-02 | 1.70E-01 |
| BP | negative regulation of leukocyte cell-cell adhesion |  | 2 | 3.8 | 1.60E-02 | 1.70E-01 |
| BP | auditory behavior |  | 2 | 3.8 | 1.60E-02 | 1.70E-01 |
| BP | positive regulation of apoptotic DNA fragmentation |  | 2 | 3.8 | 1.60E-02 | 1.70E-01 |
| BP | negative regulation of immature T cell proliferation in thymus |  | 2 | 3.8 | 1.60E-02 | 1.70E-01 |
| BP | positive regulation of epithelial cell proliferation |  | 3 | 5.7 | 1.60E-02 | 1.70E-01 |
| BP | apoptotic signaling pathway |  | 3 | 5.7 | 1.80E-02 | 1.70E-01 |
| BP | cellular response to mechanical stimulus |  | 3 | 5.7 | 1.80E-02 | 1.70E-01 |
| BP | response to toxic substance |  | 3 | 5.7 | 1.80E-02 | 1.70E-01 |
| BP | positive regulation of osteoblast differentiation |  | 3 | 5.7 | 1.80E-02 | 1.70E-01 |
| BP | response to nematode |  | 2 | 3.8 | 1.90E-02 | 1.70E-01 |
| BP | hyaloid vascular plexus regression |  | 2 | 3.8 | 1.90E-02 | 1.70E-01 |
| BP | positive regulation of macromolecule metabolic process |  | 2 | 3.8 | 1.90E-02 | 1.70E-01 |
| BP | T-helper 17 cell lineage commitment |  | 2 | 3.8 | 1.90E-02 | 1.70E-01 |
| BP | trachea formation |  | 2 | 3.8 | 1.90E-02 | 1.70E-01 |
| BP | positive regulation of I-kappaB phosphorylation |  | 2 | 3.8 | 1.90E-02 | 1.70E-01 |
| BP | response to leptin |  | 2 | 3.8 | 1.90E-02 | 1.70E-01 |
| BP | adenohypophysis development |  | 2 | 3.8 | 1.90E-02 | 1.70E-01 |
| BP | common-partner SMAD protein phosphorylation |  | 2 | 3.8 | 1.90E-02 | 1.70E-01 |
| BP | BMP signaling pathway involved in heart development |  | 2 | 3.8 | 1.90E-02 | 1.70E-01 |
| BP | vascular endothelial growth factor production |  | 2 | 3.8 | 1.90E-02 | 1.70E-01 |
| BP | in utero embryonic development |  | 4 | 7.5 | 2.00E-02 | 1.80E-01 |
| BP | activation of cysteine-type endopeptidase activity involved in apoptotic process |  | 3 | 5.7 | 2.00E-02 | 1.90E-01 |
| BP | protein processing |  | 3 | 5.7 | 2.10E-02 | 1.90E-01 |
| BP | luteinization |  | 2 | 3.8 | 2.10E-02 | 1.90E-01 |
| BP | ERBB2-EGFR signaling pathway |  | 2 | 3.8 | 2.10E-02 | 1.90E-01 |
| BP | response to fluid shear stress |  | 2 | 3.8 | 2.10E-02 | 1.90E-01 |
| BP | membranous septum morphogenesis |  | 2 | 3.8 | 2.10E-02 | 1.90E-01 |
| BP | negative regulation of platelet activation |  | 2 | 3.8 | 2.10E-02 | 1.90E-01 |
| BP | cellular response to virus |  | 3 | 5.7 | 2.30E-02 | 2.00E-01 |
| BP | memory |  | 3 | 5.7 | 2.30E-02 | 2.00E-01 |
| BP | ovulation from ovarian follicle |  | 2 | 3.8 | 2.40E-02 | 2.00E-01 |
| BP | monoamine transport |  | 2 | 3.8 | 2.40E-02 | 2.00E-01 |
| BP | progesterone receptor signaling pathway |  | 2 | 3.8 | 2.40E-02 | 2.00E-01 |
| BP | negative regulation of lipid storage |  | 2 | 3.8 | 2.40E-02 | 2.00E-01 |
| BP | tissue remodeling |  | 2 | 3.8 | 2.40E-02 | 2.00E-01 |
| BP | blood coagulation |  | 3 | 5.7 | 2.50E-02 | 2.10E-01 |
| BP | negative regulation of synaptic transmission, glutamatergic |  | 2 | 3.8 | 2.70E-02 | 2.20E-01 |
| BP | response to cholesterol |  | 2 | 3.8 | 2.70E-02 | 2.20E-01 |
| BP | positive regulation of glucose metabolic process |  | 2 | 3.8 | 2.70E-02 | 2.20E-01 |
| BP | hydrogen peroxide biosynthetic process |  | 2 | 3.8 | 2.70E-02 | 2.20E-01 |
| BP | negative regulation of chemokine production |  | 2 | 3.8 | 2.90E-02 | 2.40E-01 |
| BP | positive regulation of podosome assembly |  | 2 | 3.8 | 2.90E-02 | 2.40E-01 |
| BP | cellular response to insulin stimulus |  | 3 | 5.7 | 3.00E-02 | 2.40E-01 |
| BP | chemical synaptic transmission |  | 4 | 7.5 | 3.10E-02 | 2.50E-01 |
| BP | regulation of inflammatory response |  | 3 | 5.7 | 3.10E-02 | 2.50E-01 |
| BP | negative regulation of neuron migration |  | 2 | 3.8 | 3.20E-02 | 2.50E-01 |
| BP | anoikis |  | 2 | 3.8 | 3.20E-02 | 2.50E-01 |
| BP | Leydig cell differentiation |  | 2 | 3.8 | 3.20E-02 | 2.50E-01 |
| BP | atrioventricular valve morphogenesis |  | 2 | 3.8 | 3.20E-02 | 2.50E-01 |
| BP | positive regulation of leukocyte adhesion to vascular endothelial cell |  | 2 | 3.8 | 3.40E-02 | 2.60E-01 |
| BP | negative regulation of fibrinolysis |  | 2 | 3.8 | 3.40E-02 | 2.60E-01 |
| BP | neurotrophin TRK receptor signaling pathway |  | 2 | 3.8 | 3.40E-02 | 2.60E-01 |
| BP | leukocyte migration involved in inflammatory response |  | 2 | 3.8 | 3.40E-02 | 2.60E-01 |
| BP | prepulse inhibition |  | 2 | 3.8 | 3.40E-02 | 2.60E-01 |
| BP | regulation of cell cycle |  | 4 | 7.5 | 3.50E-02 | 2.60E-01 |
| BP | response to virus |  | 3 | 5.7 | 3.50E-02 | 2.70E-01 |
| BP | response to yeast |  | 2 | 3.8 | 3.70E-02 | 2.70E-01 |
| BP | behavioral response to cocaine |  | 2 | 3.8 | 3.70E-02 | 2.70E-01 |
| BP | striated muscle cell differentiation |  | 2 | 3.8 | 3.70E-02 | 2.70E-01 |
| BP | negative regulation of glycolytic process |  | 2 | 3.8 | 3.70E-02 | 2.70E-01 |
| BP | cellular calcium ion homeostasis |  | 3 | 5.7 | 3.70E-02 | 2.70E-01 |
| BP | cellular response to DNA damage stimulus |  | 4 | 7.5 | 3.80E-02 | 2.80E-01 |
| BP | glucose homeostasis |  | 3 | 5.7 | 3.90E-02 | 2.80E-01 |
| BP | positive regulation of heterotypic cell-cell adhesion |  | 2 | 3.8 | 4.00E-02 | 2.80E-01 |
| BP | negative regulation of macroautophagy |  | 2 | 3.8 | 4.00E-02 | 2.80E-01 |
| BP | detection of mechanical stimulus involved in sensory perception of pain |  | 2 | 3.8 | 4.00E-02 | 2.80E-01 |
| BP | dopamine metabolic process |  | 2 | 3.8 | 4.00E-02 | 2.80E-01 |
| BP | response to hyperoxia |  | 2 | 3.8 | 4.00E-02 | 2.80E-01 |
| BP | positive regulation of sequence-specific DNA binding transcription factor activity |  | 3 | 5.7 | 4.00E-02 | 2.80E-01 |
| BP | kidney development |  | 3 | 5.7 | 4.00E-02 | 2.80E-01 |
| BP | response to oxidative stress |  | 3 | 5.7 | 4.10E-02 | 2.80E-01 |
| BP | regulation of primary metabolic process |  | 2 | 3.8 | 4.20E-02 | 2.90E-01 |
| BP | response to corticosterone |  | 2 | 3.8 | 4.50E-02 | 3.10E-01 |
| BP | response to light stimulus |  | 2 | 3.8 | 4.50E-02 | 3.10E-01 |
| BP | negative regulation of bone resorption |  | 2 | 3.8 | 4.50E-02 | 3.10E-01 |
| BP | phosphorylation |  | 3 | 5.7 | 4.60E-02 | 3.10E-01 |
| BP | negative regulation of transcription from RNA polymerase II promoter |  | 7 | 13.2 | 4.70E-02 | 3.20E-01 |
| BP | positive regulation of DNA biosynthetic process |  | 2 | 3.8 | 4.70E-02 | 3.20E-01 |
| BP | cellular response to ATP |  | 2 | 3.8 | 4.70E-02 | 3.20E-01 |
| BP | defense response to Gram-positive bacterium |  | 3 | 5.7 | 4.80E-02 | 3.20E-01 |
| BP | positive regulation of canonical Wnt signaling pathway |  | 3 | 5.7 | 5.00E-02 | 3.20E-01 |
| BP | negative regulation of inflammatory response |  | 3 | 5.7 | 5.00E-02 | 3.20E-01 |
| BP | negative regulation of anoikis |  | 2 | 3.8 | 5.00E-02 | 3.20E-01 |
| BP | fibrinolysis |  | 2 | 3.8 | 5.00E-02 | 3.20E-01 |
| BP | negative regulation of B cell proliferation |  | 2 | 3.8 | 5.00E-02 | 3.20E-01 |
| BP | response to X-ray |  | 2 | 3.8 | 5.00E-02 | 3.20E-01 |
| BP | positive regulation of glial cell proliferation |  | 2 | 3.8 | 5.00E-02 | 3.20E-01 |
| BP | pharyngeal system development |  | 2 | 3.8 | 5.00E-02 | 3.20E-01 |
| BP | regulation of sodium ion transport |  | 2 | 3.8 | 5.00E-02 | 3.20E-01 |
| BP | neuron fate commitment |  | 2 | 3.8 | 5.00E-02 | 3.20E-01 |
| BP | response to vitamin A |  | 2 | 3.8 | 5.00E-02 | 3.20E-01 |
| BP | positive regulation of synaptic transmission |  | 2 | 3.8 | 5.00E-02 | 3.20E-01 |
| BP | positive regulation of SMAD protein import into nucleus |  | 2 | 3.8 | 5.20E-02 | 3.30E-01 |
| BP | positive regulation of lipid biosynthetic process |  | 2 | 3.8 | 5.20E-02 | 3.30E-01 |
| BP | acute inflammatory response |  | 2 | 3.8 | 5.20E-02 | 3.30E-01 |
| BP | labyrinthine layer blood vessel development |  | 2 | 3.8 | 5.20E-02 | 3.30E-01 |
| BP | positive regulation of protein localization to cell surface |  | 2 | 3.8 | 5.50E-02 | 3.40E-01 |
| BP | steroid hormone mediated signaling pathway |  | 2 | 3.8 | 5.50E-02 | 3.40E-01 |
| BP | positive regulation of beta-amyloid formation |  | 2 | 3.8 | 5.50E-02 | 3.40E-01 |
| BP | multicellular organism development |  | 3 | 5.7 | 5.70E-02 | 3.50E-01 |
| BP | negative regulation of proteolysis |  | 2 | 3.8 | 5.70E-02 | 3.50E-01 |
| BP | negative regulation of chondrocyte differentiation |  | 2 | 3.8 | 6.00E-02 | 3.60E-01 |
| BP | execution phase of apoptosis |  | 2 | 3.8 | 6.00E-02 | 3.60E-01 |
| BP | membrane protein ectodomain proteolysis |  | 2 | 3.8 | 6.00E-02 | 3.60E-01 |
| BP | cellular response to organic substance |  | 2 | 3.8 | 6.00E-02 | 3.60E-01 |
| BP | positive regulation of glycolytic process |  | 2 | 3.8 | 6.00E-02 | 3.60E-01 |
| BP | negative regulation of neurogenesis |  | 2 | 3.8 | 6.30E-02 | 3.70E-01 |
| BP | negative regulation of pri-miRNA transcription from RNA polymerase II promoter |  | 2 | 3.8 | 6.30E-02 | 3.70E-01 |
| BP | positive regulation of cytosolic calcium ion concentration |  | 3 | 5.7 | 6.40E-02 | 3.80E-01 |
| BP | outflow tract septum morphogenesis |  | 2 | 3.8 | 6.50E-02 | 3.80E-01 |
| BP | regulation of protein binding |  | 2 | 3.8 | 6.50E-02 | 3.80E-01 |
| BP | positive regulation of macrophage cytokine production |  | 2 | 3.8 | 6.50E-02 | 3.80E-01 |
| BP | artery morphogenesis |  | 2 | 3.8 | 6.50E-02 | 3.80E-01 |
| BP | positive regulation of nitric-oxide synthase activity |  | 2 | 3.8 | 6.50E-02 | 3.80E-01 |
| BP | MAPK cascade |  | 3 | 5.7 | 6.50E-02 | 3.80E-01 |
| BP | negative regulation of myoblast differentiation |  | 2 | 3.8 | 7.00E-02 | 4.00E-01 |
| BP | positive regulation of immunoglobulin production |  | 2 | 3.8 | 7.00E-02 | 4.00E-01 |
| BP | regulation of postsynaptic membrane potential |  | 2 | 3.8 | 7.00E-02 | 4.00E-01 |
| BP | positive regulation of angiogenesis |  | 3 | 5.7 | 7.20E-02 | 4.00E-01 |
| BP | response to radiation |  | 2 | 3.8 | 7.30E-02 | 4.00E-01 |
| BP | positive regulation of G2/M transition of mitotic cell cycle |  | 2 | 3.8 | 7.30E-02 | 4.00E-01 |
| BP | pyroptosis |  | 2 | 3.8 | 7.30E-02 | 4.00E-01 |
| BP | digestive tract development |  | 2 | 3.8 | 7.30E-02 | 4.00E-01 |
| BP | regulation of ERK1 and ERK2 cascade |  | 2 | 3.8 | 7.30E-02 | 4.00E-01 |
| BP | positive regulation of collagen biosynthetic process |  | 2 | 3.8 | 7.30E-02 | 4.00E-01 |
| BP | negative regulation of blood pressure |  | 2 | 3.8 | 7.50E-02 | 4.10E-01 |
| BP | insulin-like growth factor receptor signaling pathway |  | 2 | 3.8 | 7.50E-02 | 4.10E-01 |
| BP | neuron differentiation |  | 3 | 5.7 | 7.60E-02 | 4.20E-01 |
| BP | activation of phospholipase C activity |  | 2 | 3.8 | 7.80E-02 | 4.20E-01 |
| BP | response to cocaine |  | 2 | 3.8 | 7.80E-02 | 4.20E-01 |
| BP | macrophage differentiation |  | 2 | 3.8 | 7.80E-02 | 4.20E-01 |
| BP | regulation of dopamine secretion |  | 2 | 3.8 | 7.80E-02 | 4.20E-01 |
| BP | regulation of presynaptic membrane potential |  | 2 | 3.8 | 7.80E-02 | 4.20E-01 |
| BP | positive regulation of vascular endothelial growth factor production |  | 2 | 3.8 | 7.80E-02 | 4.20E-01 |
| BP | apoptotic process |  | 5 | 9.4 | 7.90E-02 | 4.20E-01 |
| BP | endodermal cell differentiation |  | 2 | 3.8 | 8.00E-02 | 4.20E-01 |
| BP | positive regulation of vasoconstriction |  | 2 | 3.8 | 8.00E-02 | 4.20E-01 |
| BP | estrogen metabolic process |  | 2 | 3.8 | 8.00E-02 | 4.20E-01 |
| BP | maintenance of permeability of blood-brain barrier |  | 2 | 3.8 | 8.00E-02 | 4.20E-01 |
| BP | positive regulation of multicellular organism growth |  | 2 | 3.8 | 8.30E-02 | 4.30E-01 |
| BP | positive regulation of phosphatidylinositol 3-kinase activity |  | 2 | 3.8 | 8.30E-02 | 4.30E-01 |
| BP | face morphogenesis |  | 2 | 3.8 | 8.30E-02 | 4.30E-01 |
| BP | branching involved in blood vessel morphogenesis |  | 2 | 3.8 | 8.30E-02 | 4.30E-01 |
| BP | cellular response to BMP stimulus |  | 2 | 3.8 | 8.30E-02 | 4.30E-01 |
| BP | innate immune response |  | 5 | 9.4 | 8.30E-02 | 4.30E-01 |
| BP | positive regulation of reactive oxygen species metabolic process |  | 2 | 3.8 | 8.50E-02 | 4.30E-01 |
| BP | adipose tissue development |  | 2 | 3.8 | 8.50E-02 | 4.30E-01 |
| BP | skeletal system morphogenesis |  | 2 | 3.8 | 8.50E-02 | 4.30E-01 |
| BP | T cell homeostasis |  | 2 | 3.8 | 8.70E-02 | 4.40E-01 |
| BP | endothelial cell migration |  | 2 | 3.8 | 9.00E-02 | 4.50E-01 |
| BP | regulation of angiogenesis |  | 2 | 3.8 | 9.00E-02 | 4.50E-01 |
| BP | odontogenesis |  | 2 | 3.8 | 9.00E-02 | 4.50E-01 |
| BP | intracellular receptor signaling pathway |  | 2 | 3.8 | 9.00E-02 | 4.50E-01 |
| BP | response to tumor necrosis factor |  | 2 | 3.8 | 9.20E-02 | 4.60E-01 |
| BP | aortic valve morphogenesis |  | 2 | 3.8 | 9.20E-02 | 4.60E-01 |
| BP | negative regulation of extrinsic apoptotic signaling pathway in absence of ligand |  | 2 | 3.8 | 9.50E-02 | 4.70E-01 |
| BP | long-term memory |  | 2 | 3.8 | 9.50E-02 | 4.70E-01 |
| BP | Wnt signaling pathway |  | 3 | 5.7 | 9.70E-02 | 4.80E-01 |
| BP | activation of protein kinase activity |  | 2 | 3.8 | 9.70E-02 | 4.80E-01 |
| BP | positive regulation of epithelial cell migration |  | 2 | 3.8 | 9.70E-02 | 4.80E-01 |
| BP | acute-phase response |  | 2 | 3.8 | 1.00E-01 | 4.80E-01 |
| BP | negative regulation of endothelial cell proliferation |  | 2 | 3.8 | 1.00E-01 | 4.80E-01 |
| CC | membrane raft |  | 9 | 17 | 1.50E-07 | 2.80E-05 |
| CC | neuronal cell body |  | 9 | 17 | 3.60E-06 | 3.40E-04 |
| CC | receptor complex |  | 7 | 13.2 | 1.80E-05 | 1.10E-03 |
| CC | cell surface |  | 10 | 18.9 | 2.80E-05 | 1.30E-03 |
| CC | integral component of plasma membrane |  | 14 | 26.4 | 4.30E-05 | 1.60E-03 |
| CC | plasma membrane |  | 28 | 52.8 | 5.40E-05 | 1.70E-03 |
| CC | extracellular space |  | 16 | 30.2 | 6.20E-05 | 1.70E-03 |
| CC | postsynaptic membrane |  | 6 | 11.3 | 1.40E-04 | 3.20E-03 |
| CC | integral component of postsynaptic membrane |  | 4 | 7.5 | 2.00E-04 | 4.20E-03 |
| CC | presynaptic membrane |  | 5 | 9.4 | 2.80E-04 | 5.30E-03 |
| CC | integral component of presynaptic membrane |  | 4 | 7.5 | 4.00E-04 | 6.90E-03 |
| CC | extrinsic component of cytoplasmic side of plasma membrane |  | 4 | 7.5 | 6.20E-04 | 9.70E-03 |
| CC | caveola |  | 4 | 7.5 | 9.00E-04 | 1.30E-02 |
| CC | extracellular region |  | 14 | 26.4 | 1.90E-03 | 2.60E-02 |
| CC | neuron projection |  | 6 | 11.3 | 2.30E-03 | 2.80E-02 |
| CC | glutamatergic synapse |  | 6 | 11.3 | 3.70E-03 | 4.40E-02 |
| CC | cyclin D1-CDK4 complex |  | 2 | 3.8 | 5.00E-03 | 5.60E-02 |
| CC | external side of plasma membrane |  | 6 | 11.3 | 7.10E-03 | 7.40E-02 |
| CC | presynapse |  | 4 | 7.5 | 9.40E-03 | 9.30E-02 |
| CC | basal plasma membrane |  | 3 | 5.7 | 1.20E-02 | 1.10E-01 |
| CC | axon |  | 5 | 9.4 | 1.20E-02 | 1.10E-01 |
| CC | transforming growth factor beta1-type II receptor-type I receptor complex |  | 2 | 3.8 | 1.30E-02 | 1.10E-01 |
| CC | neuromuscular junction |  | 3 | 5.7 | 1.70E-02 | 1.30E-01 |
| CC | caspase complex |  | 2 | 3.8 | 1.80E-02 | 1.30E-01 |
| CC | activin receptor complex |  | 2 | 3.8 | 1.80E-02 | 1.30E-01 |
| CC | extracellular exosome |  | 12 | 22.6 | 2.10E-02 | 1.50E-01 |
| CC | plasma membrane region |  | 2 | 3.8 | 2.30E-02 | 1.60E-01 |
| CC | dendrite |  | 5 | 9.4 | 2.50E-02 | 1.70E-01 |
| CC | ruffle membrane |  | 3 | 5.7 | 2.70E-02 | 1.70E-01 |
| CC | endosome membrane |  | 4 | 7.5 | 2.70E-02 | 1.70E-01 |
| CC | macromolecular complex |  | 6 | 11.3 | 2.90E-02 | 1.70E-01 |
| CC | cytoplasm |  | 22 | 41.5 | 2.90E-02 | 1.70E-01 |
| CC | perinuclear region of cytoplasm |  | 6 | 11.3 | 3.70E-02 | 2.10E-01 |
| CC | synaptic vesicle membrane |  | 3 | 5.7 | 3.90E-02 | 2.10E-01 |
| CC | membrane |  | 16 | 30.2 | 4.00E-02 | 2.10E-01 |
| CC | endoplasmic reticulum lumen |  | 4 | 7.5 | 4.20E-02 | 2.10E-01 |
| CC | bicellular tight junction |  | 3 | 5.7 | 4.30E-02 | 2.10E-01 |
| CC | cytosol |  | 21 | 39.6 | 4.40E-02 | 2.10E-01 |
| CC | synapse |  | 5 | 9.4 | 4.50E-02 | 2.10E-01 |
| CC | postsynapse |  | 3 | 5.7 | 4.60E-02 | 2.10E-01 |
| CC | serine-type endopeptidase complex |  | 2 | 3.8 | 5.40E-02 | 2.50E-01 |
| CC | endoplasmic reticulum membrane |  | 7 | 13.2 | 5.90E-02 | 2.70E-01 |
| CC | mitochondrion |  | 8 | 15.1 | 6.90E-02 | 3.00E-01 |
| CC | mitochondrial outer membrane |  | 3 | 5.7 | 9.40E-02 | 3.90E-01 |
| CC | synaptic membrane |  | 2 | 3.8 | 9.40E-02 | 3.90E-01 |
| MF | endopeptidase activity |  | 7 | 13.2 | 6.60E-08 | 2.00E-05 |
| MF | serine-type endopeptidase activity |  | 8 | 15.1 | 7.90E-07 | 8.00E-05 |
| MF | protein kinase activity |  | 10 | 18.9 | 8.00E-07 | 8.00E-05 |
| MF | receptor binding |  | 10 | 18.9 | 1.50E-06 | 1.10E-04 |
| MF | protein binding |  | 50 | 94.3 | 2.20E-06 | 1.30E-04 |
| MF | protein serine/threonine/tyrosine kinase activity |  | 10 | 18.9 | 2.80E-06 | 1.40E-04 |
| MF | protein tyrosine kinase activity |  | 6 | 11.3 | 1.40E-05 | 5.80E-04 |
| MF | phospholipase activator activity |  | 4 | 7.5 | 1.60E-05 | 5.80E-04 |
| MF | identical protein binding |  | 16 | 30.2 | 3.40E-05 | 1.10E-03 |
| MF | enzyme binding |  | 8 | 15.1 | 7.70E-05 | 2.30E-03 |
| MF | ATP binding |  | 14 | 26.4 | 2.00E-04 | 5.60E-03 |
| MF | protease binding |  | 5 | 9.4 | 2.30E-04 | 5.70E-03 |
| MF | non-membrane spanning protein tyrosine kinase activity |  | 4 | 7.5 | 2.60E-04 | 5.90E-03 |
| MF | transmembrane receptor protein serine/threonine kinase activity |  | 3 | 5.7 | 3.30E-04 | 7.10E-03 |
| MF | macromolecular complex binding |  | 7 | 13.2 | 4.10E-04 | 8.10E-03 |
| MF | activin binding |  | 3 | 5.7 | 7.60E-04 | 1.40E-02 |
| MF | heme binding |  | 5 | 9.4 | 8.20E-04 | 1.50E-02 |
| MF | metallopeptidase activity |  | 4 | 7.5 | 1.00E-03 | 1.70E-02 |
| MF | ATPase binding |  | 4 | 7.5 | 1.70E-03 | 2.70E-02 |
| MF | protein phosphatase binding |  | 4 | 7.5 | 1.90E-03 | 2.80E-02 |
| MF | transforming growth factor beta binding |  | 3 | 5.7 | 2.00E-03 | 2.80E-02 |
| MF | kinase binding |  | 4 | 7.5 | 2.80E-03 | 3.80E-02 |
| MF | peptidase activity |  | 4 | 7.5 | 3.00E-03 | 3.90E-02 |
| MF | kinase activity |  | 5 | 9.4 | 3.20E-03 | 3.90E-02 |
| MF | oxidoreductase activity |  | 5 | 9.4 | 3.30E-03 | 3.90E-02 |
| MF | growth factor binding |  | 3 | 5.7 | 4.40E-03 | 5.10E-02 |
| MF | protein serine/threonine kinase activity |  | 6 | 11.3 | 4.60E-03 | 5.10E-02 |
| MF | metalloendopeptidase activity |  | 4 | 7.5 | 4.80E-03 | 5.20E-02 |
| MF | transcription coactivator binding |  | 3 | 5.7 | 7.10E-03 | 7.40E-02 |
| MF | monoamine oxidase activity |  | 2 | 3.8 | 8.20E-03 | 8.30E-02 |
| MF | SMAD binding |  | 3 | 5.7 | 8.70E-03 | 8.40E-02 |
| MF | transmembrane receptor protein tyrosine kinase activity |  | 3 | 5.7 | 9.30E-03 | 8.50E-02 |
| MF | zinc ion binding |  | 8 | 15.1 | 9.40E-03 | 8.50E-02 |
| MF | RNA polymerase II transcription factor activity, ligand-activated sequence-specific DNA binding |  | 3 | 5.7 | 9.70E-03 | 8.60E-02 |
| MF | growth factor activity |  | 4 | 7.5 | 1.10E-02 | 8.90E-02 |
| MF | aliphatic-amine oxidase activity |  | 2 | 3.8 | 1.10E-02 | 8.90E-02 |
| MF | phenethylamine:oxygen oxidoreductase (deaminating) activity |  | 2 | 3.8 | 1.10E-02 | 8.90E-02 |
| MF | heparin binding |  | 4 | 7.5 | 1.20E-02 | 9.80E-02 |
| MF | activin receptor activity, type I |  | 2 | 3.8 | 1.40E-02 | 1.00E-01 |
| MF | transforming growth factor beta receptor activity, type I |  | 2 | 3.8 | 1.40E-02 | 1.00E-01 |
| MF | electron carrier activity |  | 3 | 5.7 | 1.50E-02 | 1.10E-01 |
| MF | transmembrane signaling receptor activity |  | 4 | 7.5 | 1.50E-02 | 1.10E-01 |
| MF | cytokine activity |  | 4 | 7.5 | 1.60E-02 | 1.10E-01 |
| MF | primary amine oxidase activity |  | 2 | 3.8 | 1.60E-02 | 1.10E-01 |
| MF | dopamine binding |  | 2 | 3.8 | 1.90E-02 | 1.30E-01 |
| MF | nitric-oxide synthase regulator activity |  | 2 | 3.8 | 2.20E-02 | 1.40E-01 |
| MF | monoamine transmembrane transporter activity |  | 2 | 3.8 | 2.40E-02 | 1.60E-01 |
| MF | cysteine-type endopeptidase activity involved in apoptotic signaling pathway |  | 2 | 3.8 | 2.70E-02 | 1.60E-01 |
| MF | transforming growth factor beta-activated receptor activity |  | 2 | 3.8 | 2.70E-02 | 1.60E-01 |
| MF | heterocyclic compound binding |  | 2 | 3.8 | 2.70E-02 | 1.60E-01 |
| MF | serotonin binding |  | 2 | 3.8 | 3.50E-02 | 2.10E-01 |
| MF | BMP receptor binding |  | 2 | 3.8 | 3.80E-02 | 2.20E-01 |
| MF | ligand-gated ion channel activity involved in regulation of presynaptic membrane potential |  | 2 | 3.8 | 4.60E-02 | 2.60E-01 |
| MF | neurotransmitter transporter activity |  | 2 | 3.8 | 4.80E-02 | 2.70E-01 |
| MF | protein homodimerization activity |  | 6 | 11.3 | 4.90E-02 | 2.70E-01 |
| MF | phospholipase binding |  | 2 | 3.8 | 6.10E-02 | 3.20E-01 |
| MF | steroid hormone receptor activity |  | 2 | 3.8 | 6.10E-02 | 3.20E-01 |
| MF | cyclin-dependent protein serine/threonine kinase regulator activity |  | 2 | 3.8 | 8.20E-02 | 4.20E-01 |
| MF | RNA polymerase II sequence-specific DNA binding transcription factor binding |  | 3 | 5.7 | 8.90E-02 | 4.60E-01 |
| MF | oxygen binding |  | 2 | 3.8 | 9.40E-02 | 4.70E-01 |
| MF | peroxidase activity |  | 2 | 3.8 | 9.70E-02 | 4.70E-01 |
| MF | protein C-terminus binding |  | 3 | 5.7 | 9.70E-02 | 4.70E-01 |
| MF | lipopolysaccharide binding |  | 2 | 3.8 | 9.90E-02 | 4.80E-01 |

# Table S1E KEGG pathway analysis

| **ID** | **Description** | **GeneRatio** | **BgRatio** | **p.adjust** | **qvalue** | **geneID** | **Count** |
| --- | --- | --- | --- | --- | --- | --- | --- |
| hsa05418 | Fluid shear stress and atherosclerosis | 20/70 | 139/7466 | 9.08E-17 | 3.77E-17 | MMP2/MMP9 | 20 |
| hsa05167 | Kaposi sarcoma-associated herpesvirus infection | 18/70 | 186/7466 | 1.48E-12 | 6.15E-13 | PTGS2/IL6/CASP3 | 18 |
| hsa04933 | AGE-RAGE signaling pathway in diabetic complications | 17/70 | 99/7466 | 1.29E-15 | 5.33E-16 | MMP2/IL6/CASP3/F3 | 17 |
| hsa04657 | IL-17 signaling pathway | 16/70 | 93/7466 | 8.38E-15 | 3.47E-15 | PTGS2/MMP9/IL6/CASP3 | 16 |
| hsa04668 | TNF signaling pathway | 16/70 | 110/7466 | 1.02E-13 | 4.25E-14 | PTGS2/MMP9/IL6/CASP3 | 16 |
| hsa05161 | Hepatitis B | 16/70 | 144/7466 | 3.99E-12 | 1.65E-12 | MMP9/IL6/CASP3 | 16 |
| hsa05142 | Chagas disease (American trypanosomiasis) | 15/70 | 102/7466 | 5.77E-13 | 2.39E-13 | IL6/IL2 | 15 |
| hsa05163 | Human cytomegalovirus infection | 15/70 | 225/7466 | 1.98E-08 | 8.20E-09 | PTGS2/IL6/CASP3 | 15 |
| hsa05215 | Prostate cancer | 13/70 | 97/7466 | 7.50E-11 | 3.11E-11 | MMP9/ERBB2 | 13 |
| hsa05160 | Hepatitis C | 13/70 | 155/7466 | 1.94E-08 | 8.06E-09 | CASP3 | 13 |
| hsa05166 | Human T-cell leukemia virus 1 infection | 13/70 | 219/7466 | 6.16E-07 | 2.56E-07 | IL6/IL2 | 13 |
| hsa04010 | MAPK signaling pathway | 13/70 | 295/7466 | 1.21E-05 | 5.01E-06 | CASP3/ERBB2 | 13 |
| hsa04151 | PI3K-Akt signaling pathway | 13/70 | 354/7466 | 6.93E-05 | 2.88E-05 | AKT1/CCND1/CDK4/EGFR/ERBB2/IL2/IL6/PDGFRA/  PIK3CA | 13 |
| hsa05169 | Epstein-Barr virus infection | 12/70 | 201/7466 | 1.69E-06 | 7.02E-07 | IL6/CASP3 | 12 |
| hsa05205 | Proteoglycans in cancer | 12/70 | 201/7466 | 1.69E-06 | 7.02E-07 | MMP2/MMP9/CASP3/  ERBB2 | 12 |
| hsa05219 | Bladder cancer | 11/70 | 41/7466 | 1.56E-12 | 6.45E-13 | MMP2/MMP9/ERBB2 | 11 |
| hsa04064 | NF-kappa B signaling pathway | 11/70 | 95/7466 | 1.65E-08 | 6.84E-09 | PTGS2 | 11 |
| hsa04659 | Th17 cell differentiation | 11/70 | 107/7466 | 3.88E-08 | 1.61E-08 | IL6/IL2 | 11 |
| hsa05145 | Toxoplasmosis | 11/70 | 113/7466 | 5.92E-08 | 2.45E-08 | CASP3 | 11 |
| hsa04210 | Apoptosis | 11/70 | 136/7466 | 2.99E-07 | 1.24E-07 | CASP3 | 11 |
| hsa05164 | Influenza A | 11/70 | 171/7466 | 2.37E-06 | 9.82E-07 | IL6 | 11 |
| hsa05152 | Tuberculosis | 11/70 | 179/7466 | 3.63E-06 | 1.51E-06 | IL6/CASP3 | 11 |
| hsa05170 | Human immunodeficiency virus 1 infection | 11/70 | 212/7466 | 1.50E-05 | 6.22E-06 | CASP3 | 11 |
| hsa05206 | MicroRNAs in cancer | 11/70 | 299/7466 | 0.000291 | 0.000121 | PTGS2/MMP9/CASP3/  ERBB2 | 11 |
| hsa05144 | Malaria | 10/70 | 49/7466 | 3.29E-10 | 1.36E-10 | IL6 | 10 |
| hsa01524 | Platinum drug resistance | 10/70 | 73/7466 | 1.74E-08 | 7.20E-09 | CASP3/ERBB2 | 10 |
| hsa05140 | Leishmaniasis | 10/70 | 74/7466 | 1.84E-08 | 7.63E-09 | PTGS2 | 10 |
| hsa05133 | Pertussis | 10/70 | 76/7466 | 1.98E-08 | 8.20E-09 | IL6/CASP3 | 10 |
| hsa05132 | Salmonella infection | 10/70 | 86/7466 | 5.68E-08 | 2.35E-08 | IL6 | 10 |
| hsa05210 | Colorectal cancer | 10/70 | 86/7466 | 5.68E-08 | 2.35E-08 | CASP3 | 10 |
| hsa05323 | Rheumatoid arthritis | 10/70 | 90/7466 | 8.06E-08 | 3.34E-08 | IL6 | 10 |
| hsa05222 | Small cell lung cancer | 10/70 | 93/7466 | 1.06E-07 | 4.41E-08 | PTGS2/CASP3 | 10 |
| hsa04066 | HIF-1 signaling pathway | 10/70 | 100/7466 | 2.07E-07 | 8.57E-08 | IL6/ERBB2 | 10 |
| hsa04620 | Toll-like receptor signaling pathway | 10/70 | 104/7466 | 2.58E-07 | 1.07E-07 | IL6 | 10 |
| hsa04625 | C-type lectin receptor signaling pathway | 10/70 | 104/7466 | 2.58E-07 | 1.07E-07 | PTGS2/IL6/IL2 | 10 |
| hsa04218 | Cellular senescence | 10/70 | 160/7466 | 9.78E-06 | 4.05E-06 | IL6 | 10 |
| hsa04621 | NOD-like receptor signaling pathway | 10/70 | 168/7466 | 1.30E-05 | 5.39E-06 | IL6 | 10 |
| hsa05225 | Hepatocellular carcinoma | 10/70 | 168/7466 | 1.30E-05 | 5.39E-06 |  | 10 |
| hsa05168 | Herpes simplex infection | 10/70 | 185/7466 | 2.90E-05 | 1.20E-05 | IL6/CASP3 | 10 |
| hsa05202 | Transcriptional misregulation in cancer | 10/70 | 186/7466 | 2.99E-05 | 1.24E-05 | MMP9/IL6/MPO | 10 |
| hsa04060 | Cytokine-cytokine receptor interaction | 10/70 | 294/7466 | 0.001076 | 0.000446 | IL6/IL2 | 10 |
| hsa05165 | Human papillomavirus infection | 10/70 | 330/7466 | 0.002264 | 0.000939 | PTGS2/CASP3 | 10 |
| hsa01522 | Endocrine resistance | 9/70 | 98/7466 | 1.69E-06 | 7.02E-07 | MMP2/MMP9/ERBB2 | 9 |
| hsa04660 | T cell receptor signaling pathway | 9/70 | 101/7466 | 1.97E-06 | 8.16E-07 | IL2 | 9 |
| hsa04380 | Osteoclast differentiation | 9/70 | 128/7466 | 1.20E-05 | 4.96E-06 |  | 9 |
| hsa04926 | Relaxin signaling pathway | 9/70 | 130/7466 | 1.27E-05 | 5.28E-06 | MMP2/MMP9 | 9 |
| hsa05224 | Breast cancer | 9/70 | 147/7466 | 3.04E-05 | 1.26E-05 | ERBB2 | 9 |
| hsa05203 | Viral carcinogenesis | 9/70 | 201/7466 | 0.000301 | 0.000125 | CASP3 | 9 |
| hsa05134 | Legionellosis | 8/70 | 55/7466 | 2.58E-07 | 1.07E-07 | IL6/CASP3 | 8 |
| hsa05223 | Non-small cell lung cancer | 8/70 | 66/7466 | 9.75E-07 | 4.04E-07 | ERBB2 | 8 |
| hsa04115 | p53 signaling pathway | 8/70 | 72/7466 | 1.72E-06 | 7.11E-07 | CASP3 | 8 |
| hsa05212 | Pancreatic cancer | 8/70 | 75/7466 | 2.23E-06 | 9.26E-07 | ERBB2 | 8 |
| hsa05146 | Amoebiasis | 8/70 | 96/7466 | 1.21E-05 | 5.01E-06 | IL6/CASP3 | 8 |
| hsa04932 | Non-alcoholic fatty liver disease (NAFLD) | 8/70 | 149/7466 | 0.000224 | 9.30E-05 | IL6/CASP3 | 8 |
| hsa05143 | African trypanosomiasis | 7/70 | 35/7466 | 2.11E-07 | 8.76E-08 | IL6 | 7 |
| hsa05213 | Endometrial cancer | 7/70 | 58/7466 | 4.78E-06 | 1.98E-06 | ERBB2 | 7 |
| hsa05321 | Inflammatory bowel disease (IBD) | 7/70 | 65/7466 | 1.00E-05 | 4.15E-06 | IL6/IL2 | 7 |
| hsa05220 | Chronic myeloid leukemia | 7/70 | 76/7466 | 2.43E-05 | 1.01E-05 |  | 7 |
| hsa04012 | ErbB signaling pathway | 7/70 | 85/7466 | 4.67E-05 | 1.94E-05 | ERBB2 | 7 |
| hsa04658 | Th1 and Th2 cell differentiation | 7/70 | 92/7466 | 7.33E-05 | 3.04E-05 | IL2 | 7 |
| hsa04068 | FoxO signaling pathway | 7/70 | 132/7466 | 0.000636 | 0.000264 | IL6 | 7 |
| hsa05162 | Measles | 7/70 | 132/7466 | 0.000636 | 0.000264 | IL6/IL2 | 7 |
| hsa05226 | Gastric cancer | 7/70 | 149/7466 | 0.001261 | 0.000523 | ERBB2 | 7 |
| hsa04630 | JAK-STAT signaling pathway | 7/70 | 162/7466 | 0.001897 | 0.000786 | IL6/IL2 | 7 |
| hsa05020 | Prion diseases | 6/70 | 35/7466 | 3.63E-06 | 1.51E-06 | IL6 | 6 |
| hsa05416 | Viral myocarditis | 6/70 | 59/7466 | 5.89E-05 | 2.44E-05 | CASP3 | 6 |
| hsa01521 | EGFR tyrosine kinase inhibitor resistance | 6/70 | 79/7466 | 0.000283 | 0.000117 | IL6/ERBB2 | 6 |
| hsa05204 | Chemical carcinogenesis | 6/70 | 82/7466 | 0.000333 | 0.000138 | PTGS2/CYP1A1 | 6 |
| hsa04726 | Serotonergic synapse | 6/70 | 115/7466 | 0.001756 | 0.000728 | PTGS2/SLC6A4/CASP3 | 6 |
| hsa04371 | Apelin signaling pathway | 6/70 | 137/7466 | 0.003989 | 0.001654 |  | 6 |
| hsa04915 | Estrogen signaling pathway | 6/70 | 137/7466 | 0.003989 | 0.001654 | MMP2/MMP9 | 6 |
| hsa04921 | Oxytocin signaling pathway | 6/70 | 152/7466 | 0.006511 | 0.002700 | PTGS2 | 6 |
| hsa04217 | Necroptosis | 6/70 | 162/7466 | 0.008267 | 0.003428 |  | 6 |
| hsa04062 | Chemokine signaling pathway | 6/70 | 190/7466 | 0.016897 | 0.007007 |  | 6 |
| hsa05016 | Huntington disease | 6/70 | 193/7466 | 0.018003 | 0.007465 | CASP3 | 6 |
| hsa04510 | Focal adhesion | 6/70 | 199/7466 | 0.020545 | 0.008519 | ERBB2 | 6 |
| hsa04215 | Apoptosis - multiple species | 5/70 | 33/7466 | 4.45E-05 | 1.85E-05 | CASP3 | 5 |
| hsa05216 | Thyroid cancer | 5/70 | 37/7466 | 7.33E-05 | 3.04E-05 |  | 5 |
| hsa05332 | Graft-versus-host disease | 5/70 | 41/7466 | 0.000119 | 4.95E-05 | IL6/IL2 | 5 |
| hsa04370 | VEGF signaling pathway | 5/70 | 59/7466 | 0.000636 | 0.000264 | PTGS2 | 5 |
| hsa05120 | Epithelial cell signaling in Helicobacter pylori infection | 5/70 | 68/7466 | 0.001153 | 0.000478 | CASP3 | 5 |
| hsa04662 | B cell receptor signaling pathway | 5/70 | 71/7466 | 0.001371 | 0.000568 |  | 5 |
| hsa05218 | Melanoma | 5/70 | 72/7466 | 0.001443 | 0.000598 |  | 5 |
| hsa05214 | Glioma | 5/70 | 75/7466 | 0.001718 | 0.000713 |  | 5 |
| hsa00980 | Metabolism of xenobiotics by cytochrome P450 | 5/70 | 76/7466 | 0.001756 | 0.000728 | CYP1A1 | 5 |
| hsa04610 | Complement and coagulation cascades | 5/70 | 79/7466 | 0.002044 | 0.000847 | F3 | 5 |
| hsa05231 | Choline metabolism in cancer | 5/70 | 99/7466 | 0.005222 | 0.002166 |  | 5 |
| hsa04670 | Leukocyte transendothelial migration | 5/70 | 112/7466 | 0.008161 | 0.003384 | MMP2/MMP9 | 5 |
| hsa04110 | Cell cycle | 5/70 | 124/7466 | 0.012051 | 0.004997 |  | 5 |
| hsa04514 | Cell adhesion molecules (CAMs) | 5/70 | 144/7466 | 0.021019 | 0.008716 |  | 5 |
| hsa05010 | Alzheimer disease | 5/70 | 171/7466 | 0.038776 | 0.016080 | CASP3 | 5 |
| hsa05330 | Allograft rejection | 4/70 | 38/7466 | 0.001153 | 0.000478 | IL2 | 4 |
| hsa04940 | Type I diabetes mellitus | 4/70 | 43/7466 | 0.001729 | 0.000717 | IL2 | 4 |
| hsa04672 | Intestinal immune network for IgA production | 4/70 | 49/7466 | 0.002638 | 0.001094 | IL6/IL2 | 4 |
| hsa05014 | Amyotrophic lateral sclerosis (ALS) | 4/70 | 51/7466 | 0.003031 | 0.001257 | CASP3 | 4 |
| hsa04623 | Cytosolic DNA-sensing pathway | 4/70 | 63/7466 | 0.006335 | 0.002627 | IL6 | 4 |
| hsa05131 | Shigellosis | 4/70 | 65/7466 | 0.006788 | 0.002815 |  | 4 |
| hsa05230 | Central carbon metabolism in cancer | 4/70 | 65/7466 | 0.006788 | 0.002815 | ERBB2 | 4 |
| hsa05221 | Acute myeloid leukemia | 4/70 | 66/7466 | 0.007098 | 0.002943 | MPO | 4 |
| hsa05211 | Renal cell carcinoma | 4/70 | 69/7466 | 0.008161 | 0.003384 |  | 4 |
| hsa04622 | RIG-I-like receptor signaling pathway | 4/70 | 70/7466 | 0.008414 | 0.003489 |  | 4 |
| hsa00982 | Drug metabolism - cytochrome P450 | 4/70 | 72/7466 | 0.009214 | 0.003821 |  | 4 |
| hsa00983 | Drug metabolism - other enzymes | 4/70 | 79/7466 | 0.012554 | 0.005206 | MPO | 4 |
| hsa04919 | Thyroid hormone signaling pathway | 4/70 | 116/7466 | 0.040880 | 0.016952 |  | 4 |
| hsa04722 | Neurotrophin signaling pathway | 4/70 | 119/7466 | 0.043625 | 0.018090 |  | 4 |
| hsa01523 | Antifolate resistance | 3/70 | 31/7466 | 0.006540 | 0.002712 | IL6 | 3 |
| hsa04913 | Ovarian steroidogenesis | 3/70 | 49/7466 | 0.020545 | 0.008519 | PTGS2/CYP1A1 | 3 |
| hsa00330 | Arginine and proline metabolism | 3/70 | 50/7466 | 0.021298 | 0.008832 |  | 3 |
| hsa05320 | Autoimmune thyroid disease | 3/70 | 53/7466 | 0.024718 | 0.010250 | IL2 | 3 |
| hsa00480 | Glutathione metabolism | 3/70 | 56/7466 | 0.028405 | 0.011779 |  | 3 |
| hsa00140 | Steroid hormone biosynthesis | 3/70 | 60/7466 | 0.033523 | 0.013901 | CYP1A1 | 3 |
| hsa00590 | Arachidonic acid metabolism | 3/70 | 63/7466 | 0.037791 | 0.015671 | PTGS2 | 3 |
| hsa00830 | Retinol metabolism | 3/70 | 67/7466 | 0.043223 | 0.017923 | CYP1A1 | 3 |
| hsa00220 | Arginine biosynthesis | 2/70 | 21/7466 | 0.029739 | 0.012332 |  | 2 |

# Table S1F Compound-target-pathway network analysis

| **Name** | **Betweenness Centrality** | **Closeness Centrality** | **Clustering Coefficient** | **Degree** |
| --- | --- | --- | --- | --- |
| STAT3 | 0.07077499 | 0.544827586 | 0.261904762 | 28 |
| PIK3CA | 0.037134688 | 0.503184713 | 0.273684211 | 20 |
| SRC | 0.041200349 | 0.509677419 | 0.284210526 | 20 |
| IL6 | 0.02734225 | 0.484662577 | 0.228070175 | 19 |
| AKT1 | 0.020312756 | 0.496855346 | 0.338235294 | 17 |
| LCK | 0.071992358 | 0.503184713 | 0.191176471 | 17 |
| MMP9 | 0.043714566 | 0.516339869 | 0.220588235 | 17 |
| TNF | 0.051020254 | 0.481707317 | 0.166666667 | 16 |
| tetrahydropalmatine | 0.062742975 | 0.49068323 | 0.028571429 | 15 |
| columbamine | 0.078155026 | 0.481707317 | 0.028571429 | 15 |
| menisperine | 0.051868646 | 0.478787879 | 0.142857143 | 15 |
| magnoflorine | 0.029971431 | 0.464705882 | 0.152380952 | 15 |
| CASP3 | 0.034977125 | 0.459302326 | 0.120879121 | 14 |
| hsa05166 | 0.025536561 | 0.456647399 | 0.217948718 | 13 |
| fibleucin | 0.06330571 | 0.470238095 | 0.051282051 | 13 |
| palmatine | 0.035655378 | 0.467455621 | 0.102564103 | 13 |
| EGFR | 0.014050552 | 0.464705882 | 0.397435897 | 13 |
| PTGS2 | 0.02609932 | 0.481707317 | 0.102564103 | 13 |
| hsa05163 | 0.006073531 | 0.438888889 | 0.333333333 | 12 |
| hsa05161 | 0.010327957 | 0.456647399 | 0.318181818 | 12 |
| hsa04933 | 0.012214967 | 0.454022989 | 0.181818182 | 12 |
| CREBBP | 0.029321292 | 0.470238095 | 0.318181818 | 12 |
| jatrorrhizine | 0.048478217 | 0.446327684 | 0.03030303 | 12 |
| MMP3 | 0.024775259 | 0.507017573 | 0.242424242 | 12 |
| hsa05164 | 0.032483225 | 0.451428571 | 0.145454545 | 11 |
| hsa05167 | 0.004131223 | 0.443820225 | 0.381818182 | 11 |
| reticuline | 0.035132318 | 0.446327684 | 0.036363636 | 11 |
| CDK4 | 0.016166166 | 0.446327684 | 0.163636364 | 11 |
| MMP2 | 0.013211728 | 0.481707317 | 0.290909091 | 11 |
| CCND1 | 0.00413748 | 0.438888889 | 0.4 | 10 |
| F3 | 0.03274771 | 0.446327684 | 0 | 10 |
| PGR | 0.020001114 | 0.464705882 | 0.177777778 | 10 |
| hsa04151 | 0.002736196 | 0.411458333 | 0.305555556 | 9 |
| hsa04668 | 0.005054829 | 0.434065934 | 0.25 | 9 |
| IL2 | 0.004692365 | 0.441340782 | 0.5 | 9 |
| XIAP | 0.017731413 | 0.415789474 | 0.194444444 | 9 |
| ERBB2 | 0.010585203 | 0.434065934 | 0.388888889 | 9 |
| TYK2 | 0.005578763 | 0.448863636 | 0.388888889 | 9 |
| neoechinulin A | 0.024398728 | 0.427027027 | 0.285714286 | 8 |
| PDGFRA | 0.00616622 | 0.427027027 | 0.428571429 | 8 |
| DRD2 | 0.010338566 | 0.396984925 | 0.214285714 | 8 |
| ACHE | 0.010814157 | 0.424731183 | 0 | 8 |
| columbin | 0.009127125 | 0.424731183 | 0.071428571 | 8 |
| ALDH2 | 0.024606793 | 0.396984925 | 0.095238095 | 7 |
| ecdysterone | 0.00761662 | 0.417989418 | 0.095238095 | 7 |
| TGFBR1 | 0.00784286 | 0.413612565 | 0.238095238 | 7 |
| SLC6A3 | 0.004627719 | 0.389162562 | 0.285714286 | 7 |
| hsa04657 | 0.00137329 | 0.407216495 | 0.333333333 | 6 |
| COMT | 0.011322065 | 0.383495146 | 0.2 | 6 |
| PLG | 0.027963451 | 0.409326425 | 0.066666667 | 6 |
| PPARA | 0.022476088 | 0.393034826 | 0.133333333 | 6 |
| CYP1A1 | 0.023248979 | 0.401015228 | 0.066666667 | 6 |
| TGFBR2 | 0.003044747 | 0.389162562 | 0.333333333 | 6 |
| SLC6A4 | 0.002672799 | 0.383495146 | 0.266666667 | 6 |
| GABRA2 | 0.029834574 | 0.401015228 | 0 | 6 |
| tinoside | 0.013269776 | 0.396984925 | 0.1 | 5 |
| BLK | 2.96E-04 | 0.411458333 | 0.9 | 5 |
| 2-deoxy-20-hydroxyecdysone-3-O-glucopyranoside | 0.006072394 | 0.422459893 | 0.2 | 5 |
| MAOA | 0.006188545 | 0.395 | 0.2 | 5 |
| stearic acid | 0.016642367 | 0.35426009 | 0 | 5 |
| palmitic acid | 0.016642367 | 0.35426009 | 0 | 5 |
| HTR2A | 0.001078164 | 0.377990431 | 0.4 | 5 |
| hsa04064 | 0.001996205 | 0.398989899 | 0 | 4 |
| CASP1 | 0.00430682 | 0.395 | 0.333333333 | 4 |
| ADH5 | 0.010543419 | 0.346491228 | 0 | 4 |
| 2-deoxy-20-hydroxyecdysone | 0.0020789 | 0.381642512 | 0.166666667 | 4 |
| F2 | 0.004631469 | 0.395 | 0 | 4 |
| CYP26A1 | 0.007190481 | 0.316 | 0 | 4 |
| MPO | 0.003438117 | 0.349557522 | 0 | 3 |
| MMP7 | 0.00357468 | 0.362385321 | 0 | 3 |
| MAOB | 0.001340319 | 0.351111111 | 0.333333333 | 3 |
| SLC1A3 | 0.001034371 | 0.36744186 | 0 | 3 |
| BMP4 | 0.001403432 | 0.349557522 | 0.333333333 | 3 |
| ELANE | 0.004886042 | 0.339055794 | 0 | 3 |
| TLR7 | 6.66E-04 | 0.333333333 | 0 | 2 |
| tinophylloloside | 0.004573745 | 0.352678571 | 0 | 2 |
| CDC45 | 6.49E-05 | 0.264214047 | 0 | 2 |
| P2RX7 | 3.84E-04 | 0.340517241 | 0 | 2 |
| ACVR1 | 0 | 0.329166667 | 1 | 2 |
| GRIA1 | 0 | 0.309803922 | 0 | 1 |
| Average | 0.01069968 | 0.423595538 | 0.17979798 | 8 |

# Table S2A All differential metabolites

| **Index** | **VIP** | **P-value** | **Formula** | **Compounds** | **Type** | **CAS** |
| --- | --- | --- | --- | --- | --- | --- |
| MEDN1112*052 | 2.16E+00 | 4.31E-07 | C16H32O3 | 16-Hydroxyhexadecanoic acid | FFA | 506-13-8 |
| MEDN1148*052 | 2.16E+00 | 4.31E-07 | C16H32O3 | 2-hydroxyhexadecanoic acid | FFA | 764-67-0 |
| MEDP2837 | 1.97E+00 | 2.23E-05 | C27H54NO7P | LPC(19:1/0:0) | LPC | - |
| MEDN1643 | 1.90E+00 | 2.30E-05 | C21H41O7P | LPA(18:1/0:0) | LPA | - |
| MEDN2293 | 2.15E+00 | 5.99E-05 | C18H20O2 | Diethylstilbestrol | Hormones and hormone related compounds | 56-53-1 |
| MEDP2783 | 1.83E+00 | 1.00E-04 | C30H58NO7P | LPC(0:0/22:2) | LPC | - |
| MEDN0112 | 2.24E+00 | 2.00E-04 | C26H43NO6 | Glycocholic Acid | Bile acids | 475-31-0 |
| MEDP2633 | 1.74E+00 | 3.11E-04 | C19H18O3 | 2-Butyl-3-(4-hydroxybenzoyl)benzofuran | Benzene and substituted derivatives | 52490-15-0 |
| MEDP1346 | 1.76E+00 | 3.57E-04 | C24H50NO7P | LPC(0:0/16:0) | LPC | - |
| MEDP2770 | 1.76E+00 | 4.25E-04 | C18H30O3 | Colneleic acid | Organic acid and Its derivatives | 52761-34-9 |
| MEDN2394 | 1.91E+00 | 4.81E-04 | C23H39O7P | LPA(20:4) | LPA | - |
| MEDN2393 | 1.69E+00 | 4.86E-04 | C21H39O7P | LPA(0:0/18:2) | LPA | - |
| MEDP1326 | 1.69E+00 | 4.93E-04 | C28H56NO7P | LPC(20:1/0:0) | LPC | - |
| MEDP1333 | 1.80E+00 | 5.44E-04 | C28H50NO7P | LPC(0:0/20:4) | LPC | - |
| MEDP2697 | 1.77E+00 | 5.49E-04 | C26H54NO6PS | 2-Thio-PAF | Others | - |
| MEDP1337 | 1.70E+00 | 6.06E-04 | C26H54NO7P | LPC(18:0/0:0) | LPC | - |
| MEDN0179 | 1.45E+00 | 6.49E-04 | C15H11I4NO4 | L-Thyroxine | Hormones and hormone related compounds | 51-48-9 |
| MEDN1079 | 1.80E+00 | 6.58E-04 | C22H32O2 | FFA(22:6) | FFA | 25167-62-8 |
| MEDN0039 | 1.67E+00 | 6.92E-04 | C10H17N3O6S | Glutathione Reducedform | Small Peptide | 70-18-8 |
| MEDP2698 | 1.68E+00 | 7.30E-04 | C18H32O4 | Octadec-9-ene-1,18-dioic-acid | Organic acid and Its derivatives | 20701-68-2 |
| MEDP2312*134 | 1.78E+00 | 8.82E-04 | C8H17N3O3 | Gly-Lys | Small Peptide | 997-62-6 |
| MEDP2545*134 | 1.78E+00 | 8.82E-04 | C8H17N3O3 | Lys-Gly | Small Peptide | 2.07E+06 |
| MEDP1689 | 1.63E+00 | 9.49E-04 | C30H58NO7P | LPC(22:2/0:0) | LPC | - |
| MEDN1224 | 1.78E+00 | 9.57E-04 | C12H24O17P2 | Bis(1-inositol)-3,1'-phosphate 1-phosphate | Alcohols | - |
| MEDP0494 | 1.63E+00 | 1.04E-03 | C23H48NO7P | LPC(15:0/0:0) | LPC | - |
| MEDP2738 | 1.61E+00 | 1.20E-03 | C23H48NO5PS | 2-Hexadecanoylthio-1-ethylphosphorylcholine | Others | 60793-01-3 |
| MEDN1272 | 1.75E+00 | 1.29E-03 | C25H48NO7P | LPE(20:2/0:0) | LPE | - |
| MEDP1692 | 1.61E+00 | 1.36E-03 | C23H48NO7P | LPC(0:0/15:0) | LPC | 108273-89-8 |
| MEDP0069 | 1.69E+00 | 1.42E-03 | C6H14N2O | N-Acetylputrescine | Organic acid and Its derivatives | 18233-70-0 |
| MEDN1600 | 1.69E+00 | 1.46E-03 | C19H39O7P | LPA(16:0/0:0) | LPA | 22002-85-3 |
| MEDP0338 | 1.62E+00 | 1.58E-03 | C24H50NO7P | LPC(16:0/0:0) | LPC | 17364-16-8 |
| MEDP2754 | 1.91E+00 | 1.73E-03 | C17H37NO | 1-desoxymethylsphinganine | SM | 1219484-98-6 |
| MEDP2824 | 1.91E+00 | 1.73E-03 | C16H33NO2 | SPH(d16:1) | SPH | - |
| MEDN2361 | 1.85E+00 | 1.73E-03 | C22H34O2 | FFA(22:5) | FFA | 2234-74-4 |
| MEDN2395 | 1.63E+00 | 1.95E-03 | C25H39O7P | LPA(22:6) | LPA | - |
| MEDP1272 | 1.62E+00 | 1.97E-03 | C27H44O8 | 20,26-dihydroxyecdysone | Hormones and hormone related compounds | 19458-46-9 |
| MEDP0089 | 1.40E+00 | 2.02E-03 | C7H11N3O | N-Acetylhistamine | Amines | 673-49-4 |
| MEDN1904 | 2.14E+00 | 2.20E-03 | C26H43NO5 | Glycodeoxycholic acid | Bile acids | 360-65-6 |
| MEDP1171 | 1.66E+00 | 2.41E-03 | C28H56NO7P | Butenoyl-PAF | Others | - |
| MEDN0383 | 1.73E+00 | 2.44E-03 | C18H32O2 | FFA(18:2) | FFA | 60-33-3 |
| MEDN0049 | 1.57E+00 | 2.66E-03 | C11H20N2O6 | L-Saccharopine | Amino acids | 997-68-2 |
| MEDN2376 | 1.58E+00 | 2.67E-03 | C24H45O9P | LPG(0:0/18:2) | LPG | - |
| MEDN2325 | 1.61E+00 | 2.69E-03 | C8H10N4O4 | 5-Acetylamino-6-formylamino-3-methyluracil | Benzene and substituted derivatives | 85438-96-6 |
| MEDP2329 | 1.80E+00 | 2.70E-03 | C11H23N3O3 | Lys-Val | Small Peptide | 20556-11-0 |
| MEDP0443*167 | 1.76E+00 | 3.09E-03 | C20H23N7O7 | 10-Formyl-Thf | Pteridines and derivatives | 2800-34-2 |
| MEDP1231*167 | 1.76E+00 | 3.09E-03 | C20H23N7O7 | Folinic acid | CoEnzyme and vitamins | 2.13E+04 |
| MEDP2521 | 1.61E+00 | 3.90E-03 | C4H10N2 | Piperazine | Heterocyclic compounds | 110-85-0 |
| MEDN2392 | 1.56E+00 | 4.24E-03 | C21H41O7P | LPA(0:0/18:1) | LPA | - |
| MEDP1799 | 1.60E+00 | 4.38E-03 | C10H7NO2 | Quinoline-2-carboxylic acid | Pteridines and derivatives | 3.42E+04 |
| MEDN1566 | 1.95E+00 | 4.52E-03 | C18H34O4 | Octadecanedioic acid | Organic acid and Its derivatives | 871-70-5 |
| MEDP0880 | 1.43E+00 | 4.59E-03 | C6H10N2O4 | N-Alpha-Acetyl-L-Asparagine | Amino acid derivatives | 4033-40-3 |
| MEDP1701 | 1.51E+00 | 4.63E-03 | C26H52NO7P | LPC(0:0/18:1) | LPC | - |
| MEDN0370 | 1.59E+00 | 4.97E-03 | C21H43O7P | LPA(0:0/18:0) | LPA | - |
| MEDP1334 | 1.54E+00 | 5.47E-03 | C28H50NO7P | LPC(20:4/0:0) | LPC | - |
| MEDP1339 | 1.44E+00 | 5.52E-03 | C26H52NO7P | LPC(18:1/0:0) | LPC | 3542-29-8 |
| MEDP1644 | 1.48E+00 | 5.78E-03 | C6H7N | Aniline | Amines | 62-53-3 |
| MEDP1331 | 1.56E+00 | 6.68E-03 | C28H52NO7P | LPC(0:0/20:3) | LPC | - |
| MEDN1329*078 | 1.84E+00 | 6.88E-03 | C26H43NO5 | Glycohyodeoxycholic acid | Bile acids | 13042-33-6 |
| MEDN0110*078 | 1.84E+00 | 6.88E-03 | C26H43NO5 | Glycoursodeoxycholic Acid | Bile acids | 64480-66-6 |
| MEDN0381 | 1.60E+00 | 6.92E-03 | C16H32O2 | FFA(16:0) | FFA | 2.11E+04 |
| MEDN2009 | 1.97E+00 | 7.30E-03 | C5H10O3 | 2-Hydroxy-3-Methyl Butanoic Acid | Organic acid and Its derivatives | 4026-18-0 |
| MEDP1484 | 1.51E+00 | 7.48E-03 | C10H7NO2 | Quinoline-4-carboxylic acid | Pteridines and derivatives | 486-74-8 |
| MEDN1271 | 1.49E+00 | 8.11E-03 | C25H48NO7P | LPE(0:0/20:2) | LPE | - |
| MEDN2308 | 1.47E+00 | 8.32E-03 | C10H19NO3 | N-Acetylaminooctanoic acid | Organic acid and Its derivatives | 5440-37-9 |
| MEDP1472 | 1.43E+00 | 8.34E-03 | C7H8N2O2 | N'-Methyl-2-pyridone-5-carboxamide | Pteridines and derivatives | 701-44-0 |
| MEDN2374 | 1.54E+00 | 8.75E-03 | C24H49O9P | LPG(18:0) | LPG | - |
| MEDP2719 | 1.57E+00 | 9.02E-03 | C12H11NO4 | (2E,4E)-6-(2-aminophenyl)-2-hydroxy-6-oxohexa-2,4-dienoic acid | Organic acid and Its derivatives | - |
| MEDN0007 | 1.31E+00 | 9.19E-03 | C6H14N4O2 | L-Arginine | Amino acids | 74-79-3 |
| MEDP0495 | 1.37E+00 | 9.43E-03 | C26H50NO7P | LPC(0:0/18:2) | LPC | - |
| MEDP1341 | 1.37E+00 | 9.55E-03 | C26H50NO7P | LPC(18:2/0:0) | LPC | - |
| MEDN1478*053 | 1.48E+00 | 9.94E-03 | C10H17N3O6 | Glu-Gln | Small Peptide | - |
| MEDN1940*053 | 1.48E+00 | 9.94E-03 | C10H17N3O6 | γ-Glu-Gln | Small Peptide | 10148-81-9 |
| MEDN1490 | 1.67E+00 | 1.05E-02 | C16H28O2 | FFA(16:2) | FFA | 28290-73-5 |
| MEDN1320 | 1.92E+00 | 1.11E-02 | C5H10O3 | 2-Hydroxy-2-Methyl Butyric acid | Organic acid and Its derivatives | 3739-30-8 |
| MEDP2792 | 1.43E+00 | 1.12E-02 | C25H54NO6P | PC(O-1:0/O-16:0) | PC-O | - |
| MEDP2745 | 1.31E+00 | 1.30E-02 | C27H52NO7P | LPC(19:2) | LPC | - |
| MEDP2555 | 1.61E+00 | 1.31E-02 | C9H15N3O4 | Pro-Asn | Small Peptide | 107856-82-6 |
| MEDP2479*143 | 1.51E+00 | 1.34E-02 | C14H16N2O2 | Cyclo(Phe-Pro) | Small Peptide | 14705-60-3 |
| MEDP1926*143 | 1.51E+00 | 1.34E-02 | C14H16N2O2 | Cyclo(Pro-Phe) | Small Peptide | 3705-26-8 |
| MEDP1336 | 1.79E+00 | 1.35E-02 | C28H48NO7P | LPC(0:0/20:5) | LPC | - |
| MEDN2368 | 1.36E+00 | 1.45E-02 | C25H50NO7P | LPE(20:1/0:0) | LPE | - |
| MEDP1335 | 1.71E+00 | 1.46E-02 | C28H48NO7P | LPC(20:5/0:0) | LPC | - |
| MEDN0322 | 1.38E+00 | 1.47E-02 | C10H12N2O3 | L-kynurenine | Amino acid derivatives | 343-65-7 |
| MEDN1589 | 1.36E+00 | 1.48E-02 | C12H20O4 | Traumatic acid | Organic acid and Its derivatives | 6402-36-4 |
| MEDP0296 | 1.48E+00 | 1.60E-02 | C5H11N3O2 | 4-Guanidinobutyric Acid | Organic acid and Its derivatives | 463-00-3 |
| MEDN0198*036 | 1.39E+00 | 1.71E-02 | C6H8O7 | Citric Acid | Organic acid and Its derivatives | 77-92-9 |
| MEDN1621*036 | 1.39E+00 | 1.71E-02 | C6H8O7 | Isocitric acid | Organic acid and Its derivatives | 320-77-4 |
| MEDN0388 | 1.51E+00 | 1.75E-02 | C18H34O2 | FFA(18:1) | FFA | 112-80-1 |
| MEDN0378*054 | 1.49E+00 | 1.81E-02 | C18H30O2 | FFA(18:3) | FFA | 506-26-3 |
| MEDN1840*054 | 1.49E+00 | 1.81E-02 | C18H30O2 | Pinolenic acid | FFA | 16833-54-8 |
| MEDN1554 | 1.64E+00 | 1.83E-02 | C2H6O4S | 2-Hydroxyethanesulfonate | Sulfonic acids | 1562-00-1 |
| MEDN0827 | 1.21E+00 | 1.93E-02 | C7H14N2O3 | N-acetylornithine | Amino acid derivatives | 1.57E+06 |
| MEDP2709 | 1.42E+00 | 1.97E-02 | C6H8O7 | 2,5-Didehydro-D-gluconate | Sugar acids | 2595-33-7 |
| MEDP2356 | 1.34E+00 | 2.00E-02 | C13H16N2O5 | Phe-Asp | Small Peptide | 22828-05-3 |
| MEDN1278 | 1.69E+00 | 2.06E-02 | C25H42NO7P | LPE(20:5/0:0) | LPE | - |
| MEDP0274 | 1.30E+00 | 2.11E-02 | C9H7NO2 | Indole-2-Carboxylic Acid | Indole and Its derivatives | 1477-50-5 |
| MEDN0579 | 1.65E+00 | 2.34E-02 | C12H15NO4 | N-lactoyl-phenylalanine | Amino acid derivatives | - |
| MEDN0394 | 1.49E+00 | 2.36E-02 | C20H36O2 | FFA(20:2) | FFA | 2091-39-6 |
| MEDN1277 | 1.75E+00 | 2.44E-02 | C25H42NO7P | LPE(0:0/20:5) | LPE | - |
| MEDN2402 | 1.31E+00 | 2.45E-02 | C21H43O9P | LPG(15:0) | LPG | - |
| MEDP0281 | 1.31E+00 | 2.51E-02 | C3H10N2 | 1,3-Diaminopropane | Amines | 109-76-2 |
| MEDP0075 | 1.25E+00 | 2.73E-02 | C8H16N4O3 | Nα-Acetyl-L-Arginine | Amino acid derivatives | 155-84-0 |
| MEDP0401 | 1.39E+00 | 2.77E-02 | C11H15N5O3S | 5'-Deoxy-5'-(Methylthio) Adenosine | Nucleotide and Its metabolites | 2457-80-9 |
| MEDN1608 | 1.40E+00 | 2.88E-02 | C22H36O2 | FFA(22:4) | FFA | 28874-58-0 |
| MEDN1069 | 1.28E+00 | 2.95E-02 | C18H28O2 | FFA(18:4) | FFA | 20290-75-9 |
| MEDN0760 | 1.31E+00 | 3.26E-02 | C20H32O3 | (±)5-HETE | Oxidized lipids | 70608-72-9 |
| MEDN1052*019 | 1.39E+00 | 3.26E-02 | C5H8O5 | 2-Hydroxyglutaric acid | Organic acid and Its derivatives | 13095-48-2 |
| MEDN0820*019 | 1.39E+00 | 3.26E-02 | C5H8O5 | 3-Hydroxyglutaric acid | Organic acid and Its derivatives | 638-18-6 |
| MEDN0206*019 | 1.39E+00 | 3.26E-02 | C5H8O5 | Citramalic Acid | Organic acid and Its derivatives | 597-44-4 |
| MEDP1343 | 1.11E+00 | 3.37E-02 | C26H48NO7P | LPC(0:0/18:3) | LPC | - |
| MEDN0333 | 1.51E+00 | 3.44E-02 | C3H4O4 | Malonic acid | Organic acid and Its derivatives | 141-82-2 |
| MEDN2178 | 1.17E+00 | 3.48E-02 | C9H8N2O2 | 4-(Hydroxyamino)quinoline 1-oxide | Benzene and substituted derivatives | 4637-56-3 |
| MEDP1056*116 | 1.37E+00 | 3.52E-02 | C4H7NO4 | Iminodiacetic acid | Organic acid and Its derivatives | 142-73-4 |
| MEDP0014*116 | 1.37E+00 | 3.52E-02 | C4H7NO4 | L-Aspartic Acid | Amino acids | 56-84-8 |
| MEDN0283*065 | 1.51E+00 | 3.57E-02 | C4H8O3 | 2-Hydroxybutanoic Acid | Organic acid and Its derivatives | 600-15-7 |
| MEDN1493*065 | 1.51E+00 | 3.57E-02 | C4H8O3 | 3-Hydroxybutanoic acid | Organic acid and Its derivatives | 300-85-6 |
| MEDP2846 | 1.24E+00 | 3.59E-02 | C28H56NO6P | LPC(O-20:2) | LPC-O | - |
| MEDN1284 | 1.35E+00 | 3.60E-02 | C23H42NO7P | LPE(18:3/0:0) | LPE | - |
| MEDP1511 | 1.04E+00 | 4.00E-02 | C10H18N2O5S | Met-Glu | Small Peptide | 14517-44-3 |
| MEDN0138 | 1.48E+00 | 4.10E-02 | C7H11N3O2 | 1-Methylhistidine | Amino acid derivatives | 332-80-9 |
| MEDP0759 | 1.25E+00 | 4.23E-02 | C30H46O4 | 18β-Glycyrrhetinic acid | Hydrocarbon derivatives | 471-53-4 |
| MEDP1476 | 1.13E+00 | 4.28E-02 | C11H14N2O | 5-Methoxytryptamine | Amines | 608-07-1 |
| MEDN1597 | 1.18E+00 | 4.34E-02 | C21H44NO6P | Phosphatidylethanolamine lyso alkenyl 16:0 | LPE | - |
| MEDP1380 | 1.22E+00 | 4.41E-02 | C27H51NO4 | Carnitine C20:1 | CAR | - |
| MEDP1218 | 1.17E+00 | 4.41E-02 | C11H9NO2 | 3-Amino-2-naphthoic acid | Phenolic acids | 5959-52-4 |
| MEDN1283 | 1.36E+00 | 4.46E-02 | C23H42NO7P | LPE(0:0/18:3) | LPE | - |

# Table S2B The information of 25 key metabolites

| **Index** | **Compounds** | **RT(min)** | **Formula** | **Q1 (Da)** | **Molecular weight (Da)** | **Ionization model** |
| --- | --- | --- | --- | --- | --- | --- |
| MEDN1224 | Bis(1-inositol)-3,1'-phosphate 1-phosphate | 0.61 | C12H24O17P2 | 501 | 502.04879 | [M-H]- |
| MEDP2312*134 | Gly-Lys | 0.57 | C8H17N3O3 | 204.1 | 203.12644 | [M+H]+ |
| MEDP2545*134 | Lys-Gly | 0.57 | C8H17N3O3 | 204.1 | 203.12644 | [M+H]+ |
| MEDN2394 | LPA(20:4) | 8.26 | C23H39O7P | 457.2 | 458.243343 | [M-H]- |
| MEDP1333 | LPC(0:0/20:4) | 8.12 | C28H50NO7P | 544.3 | 543.332491 | [M+H]+ |
| MEDP1337 | LPC(18:0/0:0) | 9.71 | C26H54NO7P | 524.4 | 523.363791 | [M+H]+ |
| MEDN2293 | Diethylstilbestrol | 5.93 | C18H20O2 | 267.1 | 268.14633 | [M-H]- |
| MEDN1079 | FFA(22:6) | 10.68 | C22H32O2 | 327.2 | 328.2402 | [M-H]- |
| MEDN1112*052 | 16-Hydroxyhexadecanoic acid | 10.49 | C16H32O3 | 271.2 | 272.2351449 | [M-H]- |
| MEDN1148*052 | 2-hydroxyhexadecanoic acid | 10.49 | C16H32O3 | 271.2 | 272.2351 | [M-H]- |
| MEDN0112 | Glycocholic Acid | 5.65 | C26H43NO6 | 464.3 | 465.309 | [M-H]- |
| MEDN0039 | Glutathione Reducedform | 1.11 | C10H17N3O6S | 306.1 | 307.084 | [M-H]- |
| MEDP2783 | LPC(0:0/22:2) | 10.29 | C30H58NO7P | 576.4 | 575.395092 | [M+H]+ |
| MEDP1689 | LPC(22:2/0:0) | 10.14 | C30H58NO7P | 576.4 | 575.395092 | [M+H]+ |
| MEDP2837 | LPC(19:1/0:0) | 9.38 | C27H54NO7P | 536.4 | 535.363791 | [M+H]+ |
| MEDP1326 | LPC(20:1/0:0) | 10.1 | C28H56NO7P | 550.4 | 549.379441 | [M+H]+ |
| MEDP2770 | Colneleic acid | 8.6 | C18H30O3 | 295.2 | 294.219495 | [M+H]+ |
| MEDP2633 | 2-Butyl-3-(4-hydroxybenzoyl)benzofuran | 8.78 | C19H18O3 | 295.1 | 294.125595 | [M+H]+ |
| MEDP2698 | Octadec-9-ene-1,18-dioic-acid | 9.09 | C18H32O4 | 295.2 | 312.23006 | [M+H-H2O]+ |
| MEDP0494 | LPC(15:0/0:0) | 8.11 | C23H48NO7P | 482.3 | 481.316841 | [M+H]+ |
| MEDN1643 | LPA(18:1/0:0) | 8.89 | C21H41O7P | 435.2 | 436.2589903 | [M-H]- |
| MEDP1346 | LPC(0:0/16:0) | 8.41 | C24H50NO7P | 496.3 | 495.332491 | [M+H]+ |
| MEDN0179 | L-Thyroxine | 5.63 | C15H11I4NO4 | 775.7 | 776.687 | [M-H]- |
| MEDN2393 | LPA(0:0/18:2) | 8.05 | C21H39O7P | 433.2 | 434.243343 | [M-H]- |
| MEDP2697 | 2-Thio-PAF | 8.87 | C26H54NO6PS | 522.3 | 539.340946 | [M+H-H2O]+ |

# Table S2C KEGG pathway analysis

| **Kegg_pathway** | **ko_ID** | **Compound A** | **Compound B** | **Compound C** | **Compound D** | **The Index of differential metabolites** |
| --- | --- | --- | --- | --- | --- | --- |
| Metabolic pathways | ko01100 | 29 | 234 | 126 | 294 | MEDP2719;MEDP0281;MEDN0138;MEDP0443*167;MEDN1112*052;MEDN1493*065;MEDP0296;MEDP0401;MEDN1224;MEDN0198*036;MEDN0381;MEDN0383;MEDN0378*054;MEDP1231*167;MEDN0039;MEDN0112;MEDN1621*036;MEDN0007;MEDN0049;MEDN0179;MEDN0322;MEDN2392;MEDN2393;MEDN2394;MEDN2395;MEDN0333;MEDP1472;MEDP0069;MEDN0827 |
| Glycine, serine and threonine metabolism | ko00260 | 1 | 11 | 126 | 294 | MEDP0281 |
| Arginine and proline metabolism | ko00330 | 4 | 14 | 126 | 294 | MEDP0281;MEDP0296;MEDN0007;MEDP0069 |
| beta-Alanine metabolism | ko00410 | 2 | 11 | 126 | 294 | MEDP0281;MEDN0333 |
| Histidine metabolism | ko00340 | 2 | 13 | 126 | 294 | MEDN0138;MEDP0089 |
| PI3K-Akt signaling pathway | ko04151 | 6 | 10 | 126 | 294 | MEDN1643;MEDP1346;MEDN2394;MEDP1333;MEDP1337;MEDP1339 |
| One carbon pool by folate | ko00670 | 2 | 2 | 126 | 294 | MEDP0443*167;MEDP1231*167 |
| Aminoacyl-tRNA biosynthesis | ko00970 | 2 | 16 | 126 | 294 | MEDP0443*167;MEDN0007 |
| Carbon metabolism | ko01200 | 3 | 18 | 126 | 294 | MEDP0443*167;MEDN0198*036;MEDN1621*036 |
| Biosynthesis of cofactors | ko01240 | 5 | 40 | 126 | 294 | MEDP0443*167;MEDN0198*036;MEDN0039;MEDN1621*036;MEDN0322 |
| Antifolate resistance | ko01523 | 1 | 2 | 126 | 294 | MEDP0443*167 |
| Propanoate metabolism | ko00640 | 1 | 4 | 126 | 294 | MEDN0283*065 |
| Insect hormone biosynthesis | ko00981 | 1 | 2 | 126 | 294 | MEDP1272 |
| Butanoate metabolism | ko00650 | 1 | 5 | 126 | 294 | MEDN1493*065 |
| cAMP signaling pathway | ko04024 | 1 | 6 | 126 | 294 | MEDN1493*065 |
| Cysteine and methionine metabolism | ko00270 | 2 | 11 | 126 | 294 | MEDP0401;MEDN0039 |
| Tryptophan metabolism | ko00380 | 2 | 16 | 126 | 294 | MEDP1476;MEDN0322 |
| Inositol phosphate metabolism | ko00562 | 1 | 3 | 126 | 294 | MEDN1224 |
| Thermogenesis | ko04714 | 1 | 9 | 126 | 294 | MEDP2867 |
| Citrate cycle (TCA cycle) | ko00020 | 2 | 4 | 126 | 294 | MEDN0198*036;MEDN1621*036 |
| Alanine, aspartate and glutamate metabolism | ko00250 | 1 | 12 | 126 | 294 | MEDN0198*036 |
| Glyoxylate and dicarboxylate metabolism | ko00630 | 2 | 10 | 126 | 294 | MEDN0198*036;MEDN1621*036 |
| 2-Oxocarboxylic acid metabolism | ko01210 | 3 | 13 | 126 | 294 | MEDN0198*036;MEDN1621*036;MEDN0827 |
| Biosynthesis of amino acids | ko01230 | 5 | 35 | 126 | 294 | MEDN0198*036;MEDN1621*036;MEDN0007;  MEDN0049;MEDN0827 |
| Taste transduction | ko04742 | 1 | 5 | 126 | 294 | MEDN0198*036 |
| Glucagon signaling pathway | ko04922 | 2 | 6 | 126 | 294 | MEDN0198*036;MEDN1621*036 |
| Central carbon metabolism in cancer | ko05230 | 3 | 22 | 126 | 294 | MEDN0198*036;MEDN1621*036;MEDN0007 |
| Fatty acid biosynthesis | ko00061 | 2 | 7 | 126 | 294 | MEDN0381;MEDN0333 |
| Fatty acid elongation | ko00062 | 1 | 1 | 126 | 294 | MEDN0381 |
| Fatty acid degradation | ko00071 | 1 | 2 | 126 | 294 | MEDN0381 |
| Biosynthesis of unsaturated fatty acids | ko01040 | 4 | 8 | 126 | 294 | MEDN0381;MEDN0383;MEDN0378*054;  MEDN0394 |
| Fatty acid metabolism | ko01212 | 2 | 2 | 126 | 294 | MEDN0381;MEDN0333 |
| Linoleic acid metabolism | ko00591 | 2 | 6 | 126 | 294 | MEDN0383;MEDN0378*054 |
| Glutathione metabolism | ko00480 | 1 | 11 | 126 | 294 | MEDN0039 |
| ABC transporters | ko02010 | 2 | 35 | 126 | 294 | MEDN0039;MEDN0007 |
| Ferroptosis | ko04216 | 1 | 7 | 126 | 294 | MEDN0039 |
| Thyroid hormone synthesis | ko04918 | 2 | 5 | 126 | 294 | MEDN0039;MEDN0179 |
| Bile secretion | ko04976 | 3 | 14 | 126 | 294 | MEDN0039;MEDN0112;MEDN0179 |
| Chemical carcinogenesis - reactive oxygen species | ko05208 | 1 | 3 | 126 | 294 | MEDN0039 |
| Diabetic cardiomyopathy | ko05415 | 1 | 5 | 126 | 294 | MEDN0039 |
| Primary bile acid biosynthesis | ko00120 | 1 | 6 | 126 | 294 | MEDN0112 |
| Cholesterol metabolism | ko04979 | 1 | 2 | 126 | 294 | MEDN0112 |
| Arginine biosynthesis | ko00220 | 2 | 9 | 126 | 294 | MEDN0007;MEDN0827 |
| Monobactam biosynthesis | ko00261 | 1 | 5 | 126 | 294 | MEDN0007 |
| D-Amino acid metabolism | ko00470 | 1 | 20 | 126 | 294 | MEDN0007 |
| mTOR signaling pathway | ko04150 | 1 | 3 | 126 | 294 | MEDN0007 |
| Protein digestion and absorption | ko04974 | 1 | 19 | 126 | 294 | MEDN0007 |
| Amyotrophic lateral sclerosis | ko05014 | 1 | 2 | 126 | 294 | MEDN0007 |
| Pathways of neurodegeneration - multiple diseases | ko05022 | 1 | 3 | 126 | 294 | MEDN0007 |
| Chagas disease | ko05142 | 1 | 1 | 126 | 294 | MEDN0007 |
| Amoebiasis | ko05146 | 1 | 2 | 126 | 294 | MEDN0007 |
| Lysine biosynthesis | ko00300 | 1 | 5 | 126 | 294 | MEDN0049 |
| Lysine degradation | ko00310 | 1 | 9 | 126 | 294 | MEDN0049 |
| Tyrosine metabolism | ko00350 | 1 | 8 | 126 | 294 | MEDN0179 |
| Neuroactive ligand-receptor interaction | ko04080 | 5 | 17 | 126 | 294 | MEDN0179;MEDN2392;MEDN2393;MEDN2394;MEDN2395 |
| Thyroid hormone signaling pathway | ko04919 | 1 | 1 | 126 | 294 | MEDN0179 |
| Autoimmune thyroid disease | ko05320 | 1 | 1 | 126 | 294 | MEDN0179 |
| African trypanosomiasis | ko05143 | 1 | 2 | 126 | 294 | MEDN0322 |
| Glycerolipid metabolism | ko00561 | 4 | 8 | 126 | 294 | MEDN2392;MEDN2393;MEDN2394;MEDN2395 |
| Glycerophospholipid metabolism | ko00564 | 7 | 22 | 126 | 294 | MEDN2392;MEDN2393;MEDN2394;MEDN2395;MEDP0338;MEDP2837;MEDP2846 |
| Phospholipase D signaling pathway | ko04072 | 4 | 9 | 126 | 294 | MEDN2392;MEDN2393;MEDN2394;MEDN2395 |
| Gap junction | ko04540 | 4 | 7 | 126 | 294 | MEDN2392;MEDN2393;MEDN2394;MEDN2395 |
| Regulation of actin cytoskeleton | ko04810 | 4 | 6 | 126 | 294 | MEDN2392;MEDN2393;MEDN2394;MEDN2395 |
| Fat digestion and absorption | ko04975 | 4 | 8 | 126 | 294 | MEDN2392;MEDN2393;MEDN2394;MEDN2395 |
| Vitamin digestion and absorption | ko04977 | 4 | 12 | 126 | 294 | MEDN2392;MEDN2393;MEDN2394;MEDN2395 |
| Pathogenic Escherichia coli infection | ko05130 | 4 | 6 | 126 | 294 | MEDN2392;MEDN2393;MEDN2394;MEDN2395 |
| Pathways in cancer | ko05200 | 4 | 10 | 126 | 294 | MEDN2392;MEDN2393;MEDN2394;MEDN2395 |
| Choline metabolism in cancer | ko05231 | 3 | 13 | 126 | 294 | MEDP0338;MEDP2837;MEDP2846 |
| Pyrimidine metabolism | ko00240 | 1 | 15 | 126 | 294 | MEDN0333 |
| Nicotinate and nicotinamide metabolism | ko00760 | 1 | 13 | 126 | 294 | MEDP1472 |
| alpha-Linolenic acid metabolism | ko00592 | 1 | 2 | 126 | 294 | MEDN1589 |

Notes: Compound A refers to the significant differential metabolites in this pathway; Compound B refers to all metabolites in this pathway;Compound C refers to all significant differential metabolites discovered in this detection; Compound D refers to all metabolites discovered in this detection.

# Table S2D The enrichment level of Metabolic pathways

| **Kegg_pathway** | **ko_ID** | **Cluster_frequency** | **Metabolome_frequency** | **P-value** | **Rich factor** | **Count** |
| --- | --- | --- | --- | --- | --- | --- |
| Neuroactive ligand-receptor interaction | ko04080 | 5 out of 126 3.96825397% | 17 out of 294 5.78231292517007% | 0.005954414 | 0.094339623 | 5 |
| Glycerophospholipid metabolism | ko00564 | 7 out of 126 5.55555556% | 22 out of 294 7.48299319727891% | 0.007209273 | 0.125167274 | 7 |
| Vitamin digestion and absorption | ko04977 | 4 out of 126 3.17460317% | 12 out of 294 4.08163265306122% | 0.010428236 | 0.102564103 | 4 |
| Biosynthesis of unsaturated fatty acids | ko01040 | 4 out of 126 3.17460317% | 8 out of 294 2.72108843537415% | 0.011910388 | 0.054054054 | 4 |
| PI3K-Akt signaling pathway | ko04151 | 6 out of 126 4.76190476% | 10 out of 294 3.40136054421769% | 0.013073574 | 0.619967995 | 6 |
| Fatty acid metabolism | ko01212 | 2 out of 126 1.58730159% | 2 out of 294 0.680272108843537% | 0.013381007 | 0.222222222 | 2 |
| One carbon pool by folate | ko00670 | 2 out of 126 1.58730159% | 2 out of 294 0.680272108843537% | 0.013381007 | 0.222222222 | 2 |
| Pathways in cancer | ko05200 | 4 out of 126 3.17460317% | 10 out of 294 3.40136054421769% | 0.014479009 | 0.129032258 | 4 |
| Phospholipase D signaling pathway | ko04072 | 4 out of 126 3.17460317% | 9 out of 294 3.06122448979592% | 0.016243121 | 0.363636364 | 4 |
| Glycerolipid metabolism | ko00561 | 4 out of 126 3.17460317% | 8 out of 294 2.72108843537415% | 0.018807824 | 0.105263158 | 4 |
| Fat digestion and absorption | ko04975 | 4 out of 126 3.17460317% | 8 out of 294 2.72108843537415% | 0.018807824 | 0.307692308 | 4 |
| Gap junction | ko04540 | 4 out of 126 3.17460317% | 7 out of 294 2.38095238095238% | 0.021765506 | 0.090909091 | 4 |
| Pathogenic Escherichia coli infection | ko05130 | 4 out of 126 3.17460317% | 6 out of 294 2.04081632653061% | 0.025174561 | 0.333333333 | 4 |
| Regulation of actin cytoskeleton | ko04810 | 4 out of 126 3.17460317% | 6 out of 294 2.04081632653061% | 0.025174561 | 0.333333333 | 4 |
| Citrate cycle (TCA cycle) | ko00020 | 2 out of 126 1.58730159% | 4 out of 294 1.36054421768707% | 0.077934732 | 0.121088435 | 2 |
| Arginine and proline metabolism | ko00330 | 4 out of 126 3.17460317% | 14 out of 294 4.76190476190476% | 0.093049835 | 0.057971014 | 4 |
| Autoimmune thyroid disease | ko05320 | 1 out of 126 0.79365079% | 1 out of 294 0.340136054421769% | 0.099489796 | 0.251360544 | 1 |
| Chagas disease | ko05142 | 1 out of 126 0.79365079% | 1 out of 294 0.340136054421769% | 0.110544218 | 0.166666667 | 1 |
| Thyroid hormone synthesis | ko04918 | 2 out of 126 1.58730159% | 5 out of 294 1.70068027210884% | 0.119751641 | 0.095238095 | 2 |
| Fatty acid elongation | ko00062 | 1 out of 126 0.79365079% | 1 out of 294 0.340136054421769% | 0.132653061 | 0.025027210 | 1 |
| Thyroid hormone signaling pathway | ko04919 | 1 out of 126 0.79365079% | 1 out of 294 0.340136054421769% | 0.132653061 | 0.090909091 | 1 |
| Linoleic acid metabolism | ko00591 | 2 out of 126 1.58730159% | 6 out of 294 2.04081632653061% | 0.182237088 | 0.071428571 | 2 |
| Glucagon signaling pathway | ko04922 | 2 out of 126 1.58730159% | 6 out of 294 2.04081632653061% | 0.182237088 | 0.076923077 | 2 |
| Fatty acid biosynthesis | ko00061 | 2 out of 126 1.58730159% | 7 out of 294 2.38095238095238% | 0.234403265 | 0.034482759 | 2 |
| 2-Oxocarboxylic acid metabolism | ko01210 | 3 out of 126 2.38095238% | 13 out of 294 4.42176870748299% | 0.24069536 | 0.272727273 | 3 |
| Choline metabolism in cancer | ko05231 | 3 out of 126 2.38095238% | 13 out of 294 4.42176870748299% | 0.24069536 | 0.272727273 | 3 |
| Fatty acid degradation | ko00071 | 1 out of 126 0.79365079% | 2 out of 294 0.680272108843537% | 0.248101971 | 0.020476190 | 1 |
| alpha-Linolenic acid metabolism | ko00592 | 1 out of 126 0.79365079% | 2 out of 294 0.680272108843537% | 0.248101971 | 0.022727273 | 1 |
| Antifolate resistance | ko01523 | 1 out of 126 0.79365079% | 2 out of 294 0.680272108843537% | 0.248101971 | 0.027027027 | 1 |
| Insect hormone biosynthesis | ko00981 | 1 out of 126 0.79365079% | 2 out of 294 0.680272108843537% | 0.248101971 | 0.041904761 | 1 |
| Amyotrophic lateral sclerosis | ko05014 | 1 out of 126 0.79365079% | 2 out of 294 0.680272108843537% | 0.248101971 | 0.071428571 | 1 |
| Amoebiasis | ko05146 | 1 out of 126 0.79365079% | 2 out of 294 0.680272108843537% | 0.248101971 | 0.076923077 | 1 |
| Cholesterol metabolism | ko04979 | 1 out of 126 0.79365079% | 2 out of 294 0.680272108843537% | 0.248101971 | 0.104190419 | 1 |
| African trypanosomiasis | ko05143 | 1 out of 126 0.79365079% | 2 out of 294 0.680272108843537% | 0.248101971 | 0.125104769 | 1 |
| Bile secretion | ko04976 | 3 out of 126 2.38095238% | 14 out of 294 4.76190476190476% | 0.279400645 | 0.024193548 | 3 |
| Arginine biosynthesis | ko00220 | 2 out of 126 1.58730159% | 9 out of 294 3.06122448979592% | 0.340167274 | 0.086956522 | 2 |
| Chemical carcinogenesis - reactive oxygen species | ko05208 | 1 out of 126 0.79365079% | 3 out of 294 1.02040816326531% | 0.348526708 | 0.01754386 | 1 |
| Inositol phosphate metabolism | ko00562 | 1 out of 126 0.79365079% | 3 out of 294 1.02040816326531% | 0.348526708 | 0.021276596 | 1 |
| Pathways of neurodegeneration - multiple diseases | ko05022 | 1 out of 126 0.79365079% | 3 out of 294 1.02040816326531% | 0.348526708 | 0.031250476 | 1 |
| mTOR signaling pathway | ko04150 | 1 out of 126 0.79365079% | 3 out of 294 1.02040816326531% | 0.348526708 | 0.514965986 | 1 |
| Glyoxylate and dicarboxylate metabolism | ko00630 | 2 out of 126 1.58730159% | 10 out of 294 3.40136054421769% | 0.391806357 | 0.031253946 | 2 |
| Propanoate metabolism | ko00640 | 1 out of 126 0.79365079% | 4 out of 294 1.36054421768707% | 0.435837561 | 0.024390244 | 1 |
| Carbon metabolism | ko01200 | 3 out of 126 2.38095238% | 18 out of 294 6.12244897959184% | 0.436432388 | 0.063829787 | 3 |
| Cysteine and methionine metabolism | ko00270 | 2 out of 126 1.58730159% | 11 out of 294 3.74149659863946% | 0.441707975 | 0.03030303 | 2 |
| beta-Alanine metabolism | ko00410 | 2 out of 126 1.58730159% | 11 out of 294 3.74149659863946% | 0.441707975 | 0.062559863 | 2 |
| Biosynthesis of amino acids | ko01230 | 5 out of 126 3.96825397% | 35 out of 294 11.9047619047619% | 0.509814672 | 0.131578947 | 5 |
| Butanoate metabolism | ko00650 | 1 out of 126 0.79365079% | 5 out of 294 1.70068027210884% | 0.511707683 | 0.021276596 | 1 |
| Diabetic cardiomyopathy | ko05415 | 1 out of 126 0.79365079% | 5 out of 294 1.70068027210884% | 0.511707683 | 0.025641026 | 1 |
| Monobactam biosynthesis | ko00261 | 1 out of 126 0.79365079% | 5 out of 294 1.70068027210884% | 0.511707683 | 0.025641026 | 1 |
| Lysine biosynthesis | ko00300 | 1 out of 126 0.79365079% | 5 out of 294 1.70068027210884% | 0.511707683 | 0.028571429 | 1 |
| Taste transduction | ko04742 | 1 out of 126 0.79365079% | 5 out of 294 1.70068027210884% | 0.511707683 | 0.03030303 | 1 |
| Histidine metabolism | ko00340 | 2 out of 126 1.58730159% | 13 out of 294 4.42176870748299% | 0.534646314 | 0.042553191 | 2 |
| Primary bile acid biosynthesis | ko00120 | 1 out of 126 0.79365079% | 6 out of 294 2.04081632653061% | 0.577601801 | 0.021276596 | 1 |
| cAMP signaling pathway | ko04024 | 1 out of 126 0.79365079% | 6 out of 294 2.04081632653061% | 0.577601801 | 0.041499866 | 1 |
| Central carbon metabolism in cancer | ko05230 | 3 out of 126 2.38095238% | 22 out of 294 7.48299319727891% | 0.580957386 | 0.081081081 | 3 |
| Ferroptosis | ko04216 | 1 out of 126 0.79365079% | 7 out of 294 2.38095238095238% | 0.634801558 | 0.032258065 | 1 |
| Biosynthesis of cofactors | ko01240 | 5 out of 126 3.96825397% | 40 out of 294 13.6054421768707% | 0.641996799 | 0.084745763 | 5 |
| Tryptophan metabolism | ko00380 | 2 out of 126 1.58730159% | 16 out of 294 5.4421768707483% | 0.65389668 | 0.024096386 | 2 |
| Aminoacyl-tRNA biosynthesis | ko00970 | 2 out of 126 1.58730159% | 16 out of 294 5.4421768707483% | 0.65389668 | 0.038461538 | 2 |
| Tyrosine metabolism | ko00350 | 1 out of 126 0.79365079% | 8 out of 294 2.72108843537415% | 0.684427827 | 0.012820513 | 1 |
| Lysine degradation | ko00310 | 1 out of 126 0.79365079% | 9 out of 294 3.06122448979592% | 0.727460396 | 0.019607843 | 1 |
| Thermogenesis | ko04714 | 1 out of 126 0.79365079% | 9 out of 294 3.06122448979592% | 0.727460396 | 0.043478261 | 1 |
| Glycine, serine and threonine metabolism | ko00260 | 1 out of 126 0.79365079% | 11 out of 294 3.74149659863946% | 0.79706002 | 0.020833333 | 1 |
| Glutathione metabolism | ko00480 | 1 out of 126 0.79365079% | 11 out of 294 3.74149659863946% | 0.79706002 | 0.026315789 | 1 |
| Alanine, aspartate and glutamate metabolism | ko00250 | 1 out of 126 0.79365079% | 12 out of 294 4.08163265306122% | 0.825027014 | 0.035714286 | 1 |
| Nicotinate and nicotinamide metabolism | ko00760 | 1 out of 126 0.79365079% | 13 out of 294 4.42176870748299% | 0.849225406 | 0.018181818 | 1 |
| Metabolic pathways | ko01100 | 2 out of 126 1.58730159% | 234 out of 294 79.5918367346939% | 0.860117026 | 0.029958678 | 29 |
| Pyrimidine metabolism | ko00240 | 1 out of 126 0.79365079% | 15 out of 294 5.10204081632653% | 0.88823747 | 0.015625598 | 1 |
| Protein digestion and absorption | ko04974 | 1 out of 126 0.79365079% | 19 out of 294 6.46258503401361% | 0.939019242 | 0.021276596 | 1 |
| D-Amino acid metabolism | ko00470 | 1 out of 126 0.79365079% | 20 out of 294 6.80272108843537% | 0.947667422 | 0.014492754 | 1 |
| ABC transporters | ko02010 | 2 out of 126 1.58730159% | 35 out of 294 11.9047619047619% | 0.964866621 | 0.014388489 | 2 |

# Table S3A Intersection pathways of network pharmacology analysis and metabolomics analysis

| **114 pathways from Network pharmacology analysis** | **71 pathways from bioinformatics analysis** | **11 pathways in the intersection** |
| --- | --- | --- |
| Fluid shear stress and atherosclerosis | Phospholipase D signaling pathway | African trypanosomiasis |
| AGE-RAGE signaling pathway in diabetic complications | Fat digestion and absorption | Amoebiasis |
| IL-17 signaling pathway | PI3K-Akt signaling pathway | PI3K-Akt signaling pathway |
| TNF signaling pathway | mTOR signaling pathway | Choline metabolism in cancer |
| Chagas disease (American trypanosomiasis) | Glycerophospholipid metabolism | Antifolate resistance |
| Kaposi sarcoma-associated herpesvirus infection | Metabolic pathways | Central carbon metabolism in cancer |
| Bladder cancer | 2-Oxocarboxylic acid metabolism | Arginine and proline metabolism |
| Hepatitis B | Choline metabolism in cancer | Autoimmune thyroid disease |
| Prostate cancer | Biosynthesis of amino acids | Glutathione metabolism |
| Malaria | Pathways in cancer | Arginine biosynthesis |
| NF-kappa B signaling pathway | Neuroactive ligand-receptor interaction | Thyroid hormone signaling pathway |
| Platinum drug resistance | One carbon pool by folate |  |
| Leishmaniasis | Fatty acid metabolism |  |
| Hepatitis C | Biosynthesis of cofactors |  |
| Pertussis | Glycerolipid metabolism |  |
| Human cytomegalovirus infection | Vitamin digestion and absorption |  |
| Th17 cell differentiation | Regulation of actin cytoskeleton |  |
| Salmonella infection | Pathogenic Escherichia coli infection |  |
| Colorectal cancer | Autoimmune thyroid disease |  |
| Toxoplasmosis | Central carbon metabolism in cancer |  |
| Rheumatoid arthritis | Arginine and proline metabolism |  |
| Small cell lung cancer | Biosynthesis of unsaturated fatty acids |  |
| HIF-1 signaling pathway | Citrate cycle (TCA cycle) |  |
| African trypanosomiasis | Carbon metabolism |  |
| Legionellosis | Thyroid hormone synthesis |  |
| Toll-like receptor signaling pathway | Arginine biosynthesis |  |
| C-type lectin receptor signaling pathway | Chagas disease |  |
| Apoptosis | Glucagon signaling pathway |  |
| Human T-cell leukemia virus 1 infection | Linoleic acid metabolism |  |
| Non-small cell lung cancer | African trypanosomiasis |  |
| Endocrine resistance | beta-Alanine metabolism |  |
| Epstein-Barr virus infection | Cholesterol metabolism |  |
| Proteoglycans in cancer | Thyroid hormone signaling pathway |  |
| p53 signaling pathway | Gap junction |  |
| T cell receptor signaling pathway | Histidine metabolism |  |
| Pancreatic cancer | Amoebiasis |  |
| Influenza A | Aminoacyl-tRNA biosynthesis |  |
| Tuberculosis | Bile secretion |  |
| Prion diseases | Amyotrophic lateral sclerosis |  |
| Endometrial cancer | Fatty acid biosynthesis |  |
| Cellular senescence | Glyoxylate and dicarboxylate metabolism |  |
| Inflammatory bowel disease (IBD) | Cysteine and methionine metabolism |  |
| Osteoclast differentiation | Tryptophan metabolism |  |
| Amoebiasis | Thermogenesis |  |
| MAPK signaling pathway | Insect hormone biosynthesis |  |
| Relaxin signaling pathway | cAMP signaling pathway |  |
| NOD-like receptor signaling pathway | Alanine, aspartate and glutamate metabolism |  |
| Hepatocellular carcinoma | Ferroptosis |  |
| Human immunodeficiency virus 1 infection | Pathways of neurodegeneration - multiple diseases |  |
| Chronic myeloid leukemia | Taste transduction |  |
| Herpes simplex infection | ABC transporters |  |
| Transcriptional misregulation in cancer | Lysine biosynthesis |  |
| Breast cancer | Antifolate resistance |  |
| Apoptosis - multiple species | Glutathione metabolism |  |
| ErbB signaling pathway | Diabetic cardiomyopathy |  |
| Viral myocarditis | Monobactam biosynthesis |  |
| PI3K-Akt signaling pathway | Fatty acid elongation |  |
| Thyroid cancer | Propanoate metabolism |  |
| Th1 and Th2 cell differentiation | alpha-Linolenic acid metabolism |  |
| Graft-versus-host disease | Inositol phosphate metabolism |  |
| Non-alcoholic fatty liver disease (NAFLD) | Butanoate metabolism |  |
| EGFR tyrosine kinase inhibitor resistance | Primary bile acid biosynthesis |  |
| MicroRNAs in cancer | Protein digestion and absorption |  |
| Viral carcinogenesis | Glycine, serine and threonine metabolism |  |
| Chemical carcinogenesis | Fatty acid degradation |  |
| VEGF signaling pathway | Lysine degradation |  |
| FoxO signaling pathway | Nicotinate and nicotinamide metabolism |  |
| Measles | Chemical carcinogenesis - reactive oxygen species |  |
| Cytokine-cytokine receptor interaction | Pyrimidine metabolism |  |
| Allograft rejection | D-Amino acid metabolism |  |
| Epithelial cell signaling in Helicobacter pylori infection | Tyrosine metabolism |  |
| Gastric cancer |  |  |
| B cell receptor signaling pathway |  |  |
| Melanoma |  |  |
| Glioma |  |  |
| Type I diabetes mellitus |  |  |
| Serotonergic synapse |  |  |
| Metabolism of xenobiotics by cytochrome P450 |  |  |
| JAK-STAT signaling pathway |  |  |
| Complement and coagulation cascades |  |  |
| Human papillomavirus infection |  |  |
| Intestinal immune network for IgA production |  |  |
| Amyotrophic lateral sclerosis (ALS) |  |  |
| Apelin signaling pathway |  |  |
| Estrogen signaling pathway |  |  |
| Choline metabolism in cancer |  |  |
| Cytosolic DNA-sensing pathway |  |  |
| Oxytocin signaling pathway |  |  |
| Antifolate resistance |  |  |
| Shigellosis |  |  |
| Central carbon metabolism in cancer |  |  |
| Acute myeloid leukemia |  |  |
| Renal cell carcinoma |  |  |
| Leukocyte transendothelial migration |  |  |
| Necroptosis |  |  |
| RIG-I-like receptor signaling pathway |  |  |
| Drug metabolism - cytochrome P450 |  |  |
| Cell cycle |  |  |
| Drug metabolism - other enzymes |  |  |
| Chemokine signaling pathway |  |  |
| Huntington disease |  |  |
| Focal adhesion |  |  |
| Ovarian steroidogenesis |  |  |
| Cell adhesion molecules (CAMs) |  |  |
| Arginine and proline metabolism |  |  |
| Autoimmune thyroid disease |  |  |
| Glutathione metabolism |  |  |
| Arginine biosynthesis |  |  |
| Steroid hormone biosynthesis |  |  |
| Arachidonic acid metabolism |  |  |
| Alzheimer disease |  |  |
| Thyroid hormone signaling pathway |  |  |
| Retinol metabolism |  |  |
| Neurotrophin signaling pathway |  |  |

# Table S3B The rank of 11 intersection pathways in network pharmacology

| **Description** | **pvalue** | **Count** |
| --- | --- | --- |
| African trypanosomiasis | 2.55996E-08 | 7 |
| Amoebiasis | 2.73044E-06 | 8 |
| PI3K-Akt signaling pathway | 1.99635E-05 | 13 |
| Choline metabolism in cancer | 0.00226825 | 5 |
| Antifolate resistance | 0.002939913 | 3 |
| Central carbon metabolism in cancer | 0.003119903 | 4 |
| Arginine and proline metabolism | 0.011294358 | 3 |
| Autoimmune thyroid disease | 0.013232949 | 3 |
| Glutathione metabolism | 0.015350236 | 3 |
| Arginine biosynthesis | 0.016221215 | 2 |
| Thyroid hormone signaling pathway | 0.023123989 | 4 |

# Table S3C The rank of 11 intersection pathways in metabolomics

| **Description** | **Rich factor** | **pvalue** | **count** |
| --- | --- | --- | --- |
| African trypanosomiasis | 0.125104769 | 0.248101971 | 1 |
| Amoebiasis | 0.076923077 | 0.248101971 | 1 |
| PI3K-Akt signaling pathway | 0.619967995 | 0.013073574 | 6 |
| Choline metabolism in cancer | 0.272727273 | 0.24069536 | 3 |
| Antifolate resistance | 0.027027027 | 0.248101971 | 1 |
| Central carbon metabolism in cancer | 0.081081081 | 0.580957386 | 3 |
| Arginine and proline metabolism | 0.057971014 | 0.093049835 | 4 |
| Autoimmune thyroid disease | 0.251360544 | 0.099489796 | 1 |
| Glutathione metabolism | 0.026315789 | 0.79706002 | 1 |
| Arginine biosynthesis | 0.086956522 | 0.340167274 | 2 |
| Thyroid hormone signaling pathway | 0.090909091 | 0.132653061 | 1 |
