# Supplementary material for: Tinosporae Radix attenuates acute pharyngitis by regulating glycerophospholipid metabolism and inflammatory responses through PI3K-Akt signaling pathway
Source: Front Pharmacol. 2024 Nov 6;15:1491321. doi: 10.3389/fphar.2024.1491321 (PMC11576305; doi:10.3389/fphar.2024.1491321)
Supplement: Supplementary file 2 [file DataSheet1.docx]

**The Content of Supplementary Figures**

**Fig.S1** Representative TIC of Control, Model, and TR group in positive and negative ion mode using UPLC-ESI-MS/MS

**Fig.S2** Four replicates of Western blotting in vivo

**Fig.S3** Four replicates of Western blotting in vitro


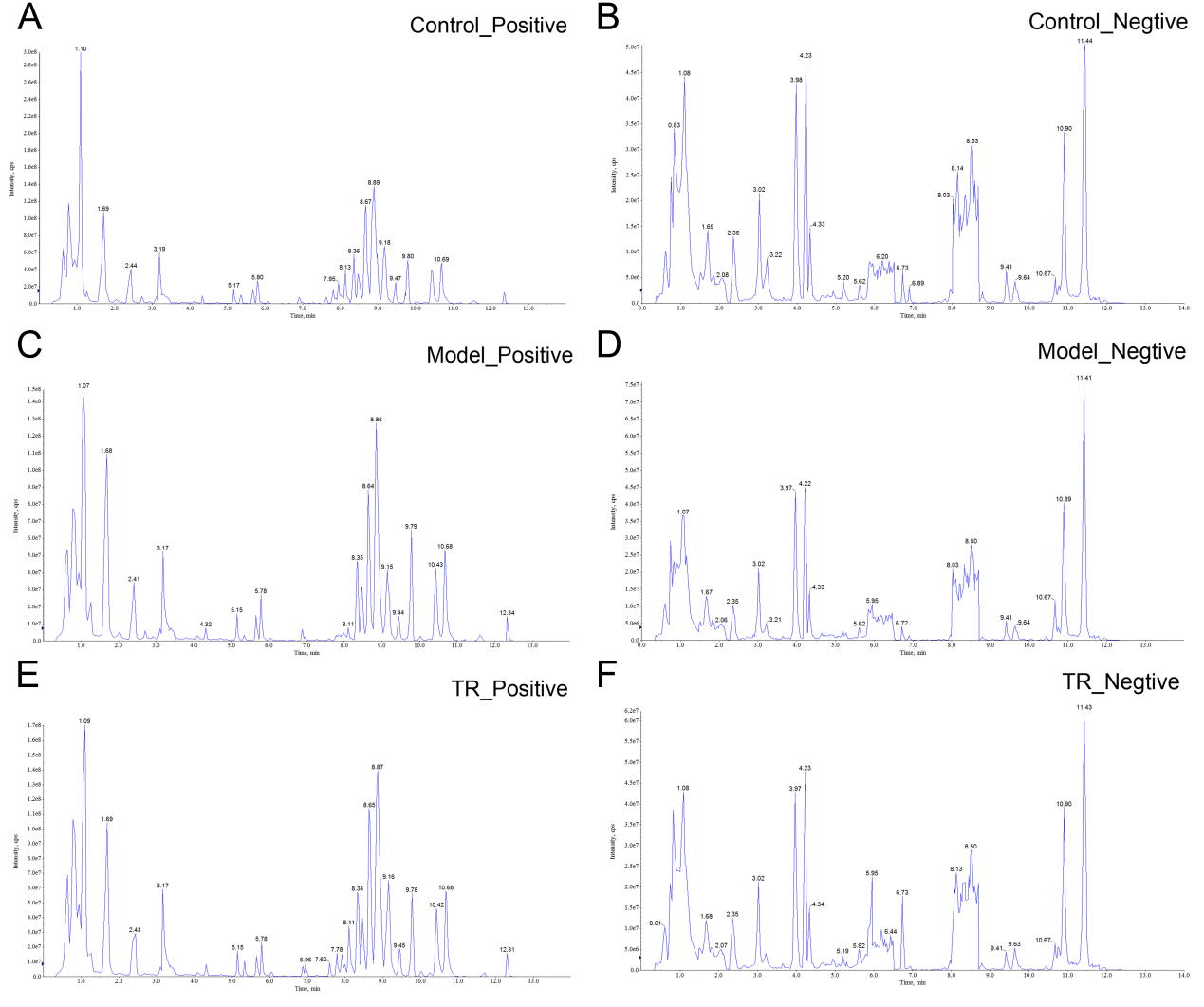


**Fig.S1** Representative TIC of Control, Model, and TR group in positive and negative ion mode using UPLC-ESI-MS/MS. (A) TIC of Control group in positive ion mode. (B) TIC of Control group in negative ion mode. (C) TIC of Model group in positive ion mode. (D) TIC of Model group in negative ion mode. (E) TIC of TR high-dose group in positive ion mode. (F) TIC of TR high-dose group in negative ion mode.


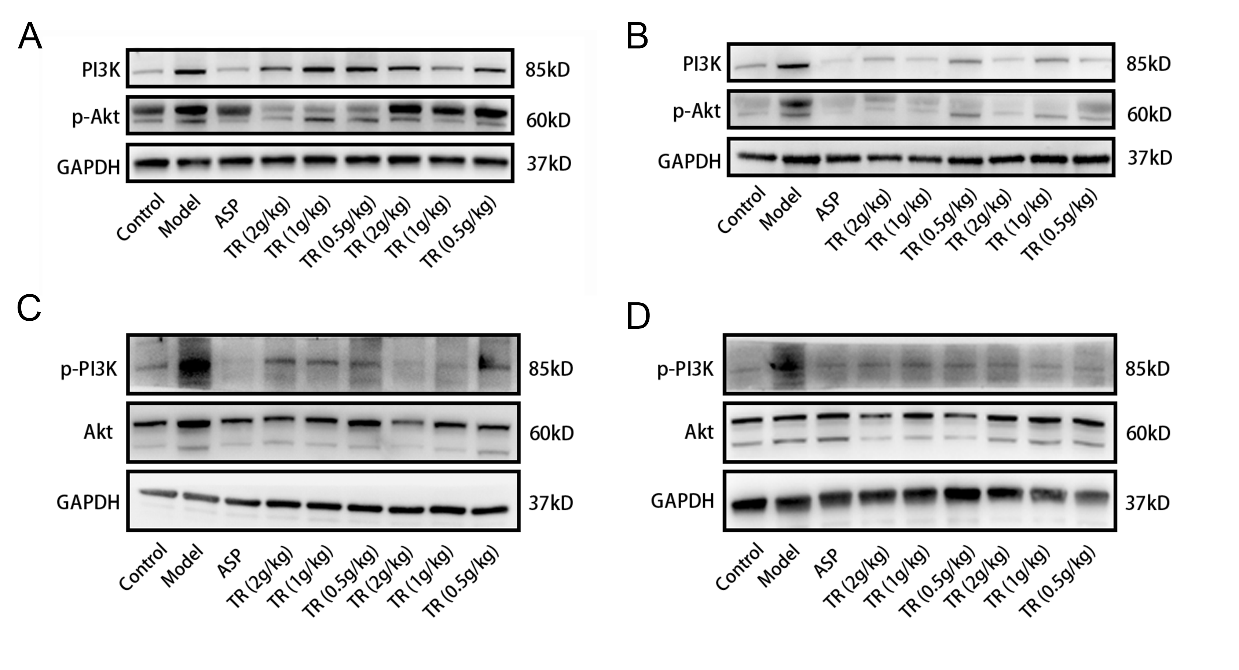


**Fig.S2** Four replicates of Western blotting in vivo. (A-B) PI3K and phosphorylated Akt protein levels in pharynx of rats in Control, Model, ASP, and low–dose/medium–dose/high–dose TR group. (C-D) Phosphorylated PI3K and Akt protein levels in pharynx of rats in Control, Model, ASP, and low–dose/medium–dose/high–dose TR group.


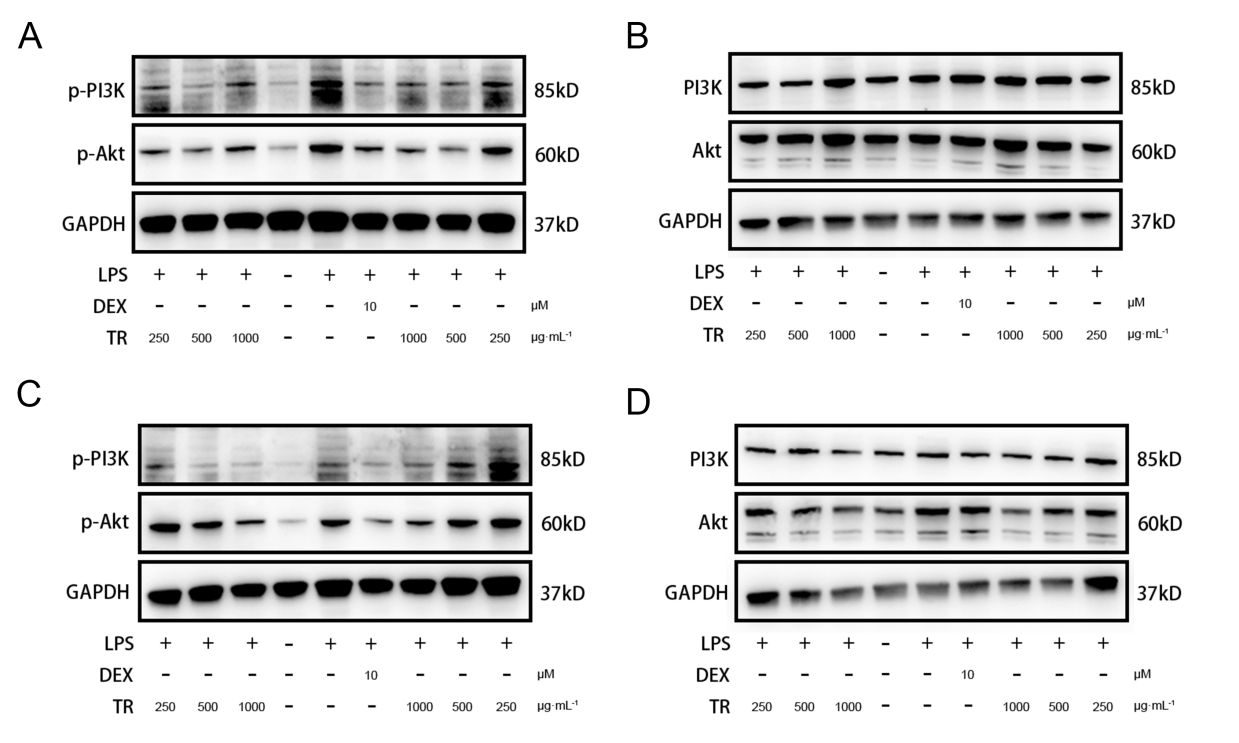


**Fig.S3** Four replicates of Western blotting in vitro. (A, C) Phosphorylated PI3K and phosphorylated Akt protein levels in RAW 264.7 macrophages of Blank, LPS, DEX, and low–dose/medium–dose/high–dose TR group. (B, D) PI3K and Akt protein levels in RAW 264.7 macrophages of Blank, LPS, DEX, and low–dose/medium–dose/high–dose TR group.
